# Synthesis, Docking Studies, and In Vitro Evaluation of Some Novel Thienopyridines and Fused Thienopyridine–Quinolines as Antibacterial Agents and DNA Gyrase Inhibitors

**DOI:** 10.3390/molecules24203650

**Published:** 2019-10-10

**Authors:** Eman M. Mohi El-Deen, Eman A. Abd El-Meguid, Sherifa Hasabelnaby, Eman A. Karam, Eman S. Nossier

**Affiliations:** 1 Department of Therapeutic Chemistry, National Research Centre, Dokki, Cairo 12622, Egypt; 2 Department of Chemistry of Natural and Microbial Products, National Research Centre, Dokki, Cairo 12622, Egypt; 3 Pharmaceutical Chemistry Department, Faculty of Pharmacy, Helwan University, Ein Helwan, Cairo 11795, Egypt; 4 Microbiology Chemistry Department, National Research Centre, Dokki, Cairo 12622, Egypt; 5 Department of Pharmaceutical Medicinal Chemistry, Faculty of Pharmacy (Girls), Al-Azhar University, Cairo 11754, Egypt

**Keywords:** thienopyridines, pyridothienoquinoline, antibacterial, MRSA, DNA gyrase, molecular docking

## Abstract

A series of novel thienopyridines and pyridothienoquinolines (**3a,b–14**) was synthesized, starting with 2-thioxo-1,2-dihydropyridine-3-carbonitriles **1a** and **1b**. All compounds were evaluated for their in vitro antimicrobial activity against six bacterial strains. Compounds **3a,b, 4a, 5b, 6a,b, 7a, 9b, 12b,** and **14** showed significant growth inhibition activity against both Gram-positive and Gram-negative bacteria compared with the reference drug. The most active compounds (**4a, 7a, 9b,** and **12b**) against *Staphylococcus aureus* were also tested for their in vitro inhibitory action on methicillin-resistant *Staphylococcus aureus* (MRSA). The tested compounds showed promising inhibition activity, with the performance of **12b** being equal to gentamicin and that of **7a** exceeding it. Moreover, the most promising compounds were also screened for their *Escherichia coli* DNA gyrase inhibitory activity, compared with novobiocin as a reference DNA gyrase inhibitor. The results revealed that compounds (**3a, 3b, 4a, 9b,** and **12b**) had the highest inhibitory capacity, with IC_50_ values of 2.26–5.87 µM (that of novobiocin is equal to 4.17 µM). Docking studies were performed to identify the mode of binding of the tested compounds to the active site of *E. coli* DNA gyrase B.

## 1. Introduction

Recently, infections of resistant bacteria have become very common [1,2] and many pathogens, mainly multi-drug resistant organisms (MDRO), have become resistant to different classes of antibiotics, such as methicillin-resistant *Staphylococcus aureus* (MRSA), which causes serious infections in hospitals [3,4]. This growing risk of antimicrobial resistance has led to the insistent need to discover new antimicrobial agents with structural features different from those of existing antibiotics [5].

The DNA gyrase enzyme has a crucial role in bacterial cell viability through its initiation of DNA replication and introduction of negative supercoils into DNA during replication [6,7]. The principal family of prokaryotic DNA-gyrase-targeted drugs is represented by fluoroquinolones, which result in the accumulation of double-strand DNA breaks, leading to bacterial cell death [8,9]. Although resistance to fluoroquinolones has appeared in the last few years, DNA gyrase remains an attractive target due to its presence across all microbes [10,11,12]. Targeting DNA gyrase with an inhibitor is a new strategy for dealing with antimicrobial resistance by disrupting DNA synthesis, leading to cell death and reducing the development of resistance [13,14].

In addition, thieno[2,3-b]pyridine derivatives have attracted great interest due to their various pharmacological activities, including anti-inflammatory [15], kinase inhibitory [16,17], anticancer [18,19,20], and antimicrobial activities [21,22,23,24,25]. Quinoline derivatives also possess important biological activities, such as anti-HIV [26,27], antimalarial [28], antihypertensive [29], and antimicrobial activities [30,31]. Furthermore, a quinolone moiety is one of the key building elements for many naturally occurring bioactive compounds [32]. Strongly effective quinolone antibiotics such as ciprofloxacin and ofloxacin selectively inhibit bacterial DNA gyrase [33], but these antibiotics are now being subjected to increasing incidences of microbial resistance, and need continuous development [34].

Therefore, as part of our research into the development of novel potential antimicrobial compounds, a series of thieno[2,3-b]pyridine derivatives was synthesized and cyclized to afford tetracyclic pyridothienoquinolines, which have both thieno[2,3-b]pyridine and quinoline bicyclic systems in the same framework, to provide effective antibacterial agents with novel structural features able to overcome bacterial resistance. The antimicrobial activity of these new compounds was evaluated by in vitro screening of their antibacterial and DNA gyrase inhibition activity. Docking studies of the most active compounds then gave insight to their binding styles at the active sites of DNA gyrase.

## 2. Results and Discussion

### 2.1. Chemistry

**Scheme 1:**
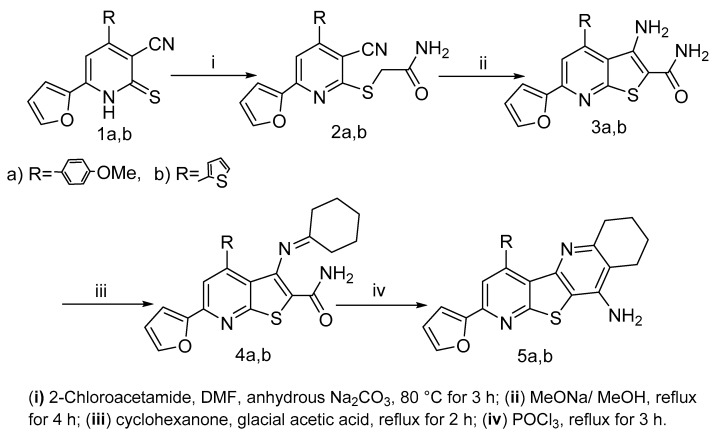
Synthesis of target compounds **3a,b**–**5a,b**.

**Scheme 2:**
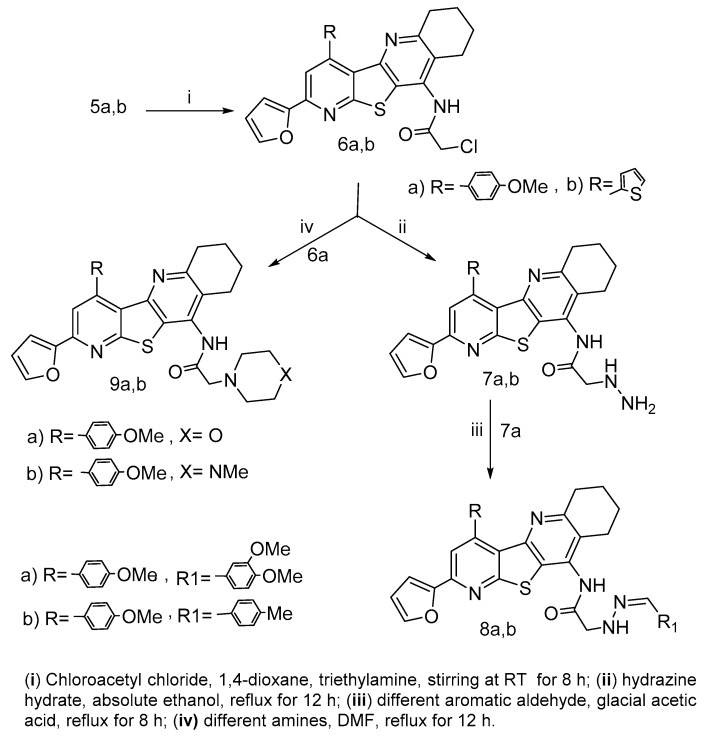
Synthesis of fused thienopyridine-quinolines **6a,b**–**9a,b**.

**Scheme 3:**
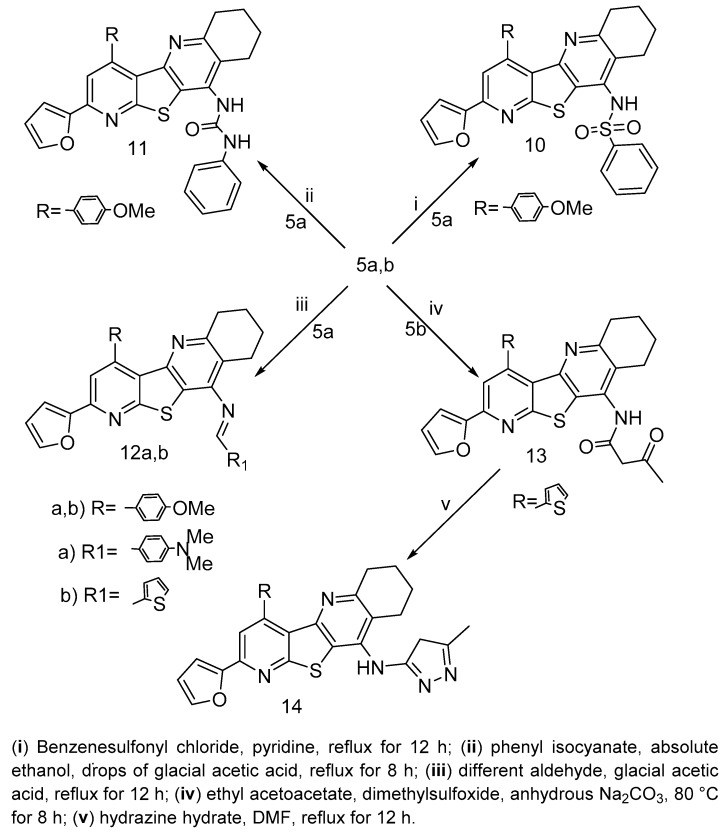
Synthesis of fused thienopyridine-quinolines 10–14.

The synthesis of new thienopyridines and fused thienopyridine–quinolines (**3a,b**–**14)** is outlined in Scheme 1, Scheme 2 and Scheme 3. The reaction of the starting compounds 2-thioxo-1,2-dihydropyridine-3-carbonitriles **1a,b** [35] with 2-chloroacetamide in *N,N*-dimethylformamide containing anhydrous sodium carbonate provided 2-((3-cyanopyridin-2-yl)thio)acetamides **2a,b,** which underwent a base-catalyzed intramolecular cyclization by refluxing with sodium methoxide in methanol to give 3-aminothieno[2,3-b]pyridine -2-carboxamides **3a,b**. IR and ^1^H NMR spectra of **3a,b** confirmed their structures by the disappearance of C≡N bands and SCH_2_ signals, which appeared in the IR and ^1^H NMR spectra of **2a,b** at 2210 and 2211 cm^−1^ and at 4.00 and 4.01 ppm, respectively. Moreover, the ^1^H NMR spectra of **3a** and **3b** showed additional signals at 5.97 and 6.20 ppm, respectively, corresponding to NH_2_ groups. Further condensation of **3a,b** with cyclohexanone in glacial acetic acid afforded 3-(cyclohexylideneamino)-thieno[2,3-b]pyridine-2-carboxamides **4a,b**. Upon refluxing of **4a,b** with phosphorous oxychloride, a cyclocondensation reaction was achieved to give 6,7,8,9- tetrahydropyrido [3′,2′:4,5]thieno[3,2-b]quinolin-10-amines **5a,b** (Scheme 1). The ^1^H NMR spectra of **4a,b** revealed three signals at 0.86–1.98 ppm, corresponding to the 5CH_2_ protons of cyclohexylidene moiety alongside the basic signals of amide–NH_2_ and aromatic H. The ^1^H NMR spectra of **5a,b** showed three signals at 1.82–2.76 ppm corresponding to the 4CH_2_ protons and NH_2_ signal at 5.53 and 6.21 ppm, respectively. In the ^13^C NMR spectra of **5a,b,** the 4CH_2_ carbons were verified by four signals at 22.75–33.20 ppm.

The tetracyclic amines **5a,b** acted as key intermediates for synthesis of a series of fused thienopyridine derivatives (**6a,b–14).** Treatment of the amines **5a,b** with chloroacetyl chloride in 1,4-dioxane containing drops of triethylamine gave chloroacetamide derivatives **6a,b**, which were reacted with hydrazine hydrate in boiling ethanol to afford hydrazinylacetamide derivatives **7a,b** The bands of acetamide C=O of **6a,b** and **7a,b** were revealed in their IR spectra in the 1659–1677 cm^−1^and 1654–1656 cm^−1^ regions, respectively. The signal of the CH_2_ protons of the chloroacetamide moiety appeared at 4.45 and 4.46 ppm in the ^1^H NMR spectra of **6a,b** and shifted upfield to 3.67 and 3.84 ppm in the ^1^H NMR spectra of **7a,b**, confirming the presence of CH_2_N from the acetohydrazinyl moiety. In addition, the ^13^C NMR spectrum of **6b** showed two signals at 43.13 ppm and 167.21 ppm, attributed to the two carbons of CH_2_Cl and C=O, respectively. Next, condensation reaction of **7a** with aromatic aldehydes, such as 3,4-dimethoxybenzaldehyde and 4-methylbenzaldehyde, in refluxing glacial acetic acid produced 3,4-dimethoxybenzylidene derivative **8a** and 4-methylbenzylidene derivative **8b**, respectively. The ^1^H NMR spectrum of **8a** confirmed arylidine formation, with signals at 3.85 ppm and 8.18 ppm corresponding to the protons of 3OCH_3_ and CH=N, respectively. Chloroacetamide derivative **6a** was reacted with morpholine and/or 1-methylpiperazine in *N,N*-dimethylformamide under reflux to give 2-morpholinoacetamide derivative **9a** and 2-(4-methylpiperazin-1-yl) acetamide derivative **9b** (Scheme 2). The presence of a morpholine moiety in the structure of **9a** was supported by its ^1^H NMR spectrum, which revealed two signals at 2.49–2.69 ppm and 3.64 ppm, corresponding to the protons of 2CH_2_N and 2CH_2_O, respectively. The ^13^ C NMR spectrum of **9a** also showed two signals at 62.12 ppm for the 2CH_2_N carbons, and at 67.11 ppm for 2CH_2_O carbons.

In addition, reaction of **5a** with benzenesulfonyl chloride in pyridine at reflux temperature gave benzenesulfonamide derivative **10**. The IR spectrum of **10** showed a band corresponding to sulfonamide NH at 3424 cm^−1^, and two bands at 1335 cm^−1^ and 1170 cm^−1^ due to sulfonamide SO_2_. Upon refluxing of amine **5a** with phenyl isocyanate in absolute ethanol, phenylurea derivative **11** was formed by nucleophilic addition reaction; the ^1^H NMR spectrum of **11** showed two signals at 9.08 ppm and 10.15 for the 2NH of the phenylurea moiety. Amine **5a** was also condensed with 4-(dimethylamino)benzaldehyde and/or thiophene-2-carboxaldehyde in boiling glacial acetic acid to produce the Schiff bases **12a** and **12b**, respectively. The structures of **12a,b** were supported by IR spectra which revealed the absence of the NH_2_ bands, which belong to the amino group of **5a.** Furthermore, the ^1^H NMR spectra of **12a** showed two signals at 2.93 ppm and 8.30 ppm besides the signals of the expected aromatic protons, attributed to N(CH_3_)_2_ and CH=N protons, respectively. Reaction of **5b** with ethyl acetoacetate in dimethylsulfoxide in the presence of anhydrous sodium carbonate gave oxobutanamide derivative **13**, which in turn was treated with hydrazine hydrate in boiling *N,N*-dimethyformamide to afford *N*-(5-methyl-4*H*-pyrazol-3-yl)-10-amine derivative **14** (Scheme 3). The chemical structures of the newly synthesized compounds (**2a,b**–**14**) were confirmed by ^1^H NMR, ^13^C NMR, and mass spectra (Supplementary Materials, Figures S1–S53), in addition to the IR spectra and correct elemental microanalyses.

### 2.2. Antimicrobial Activity Evaluation

#### 2.2.1. Antibacterial Activity

**Figure 1:**
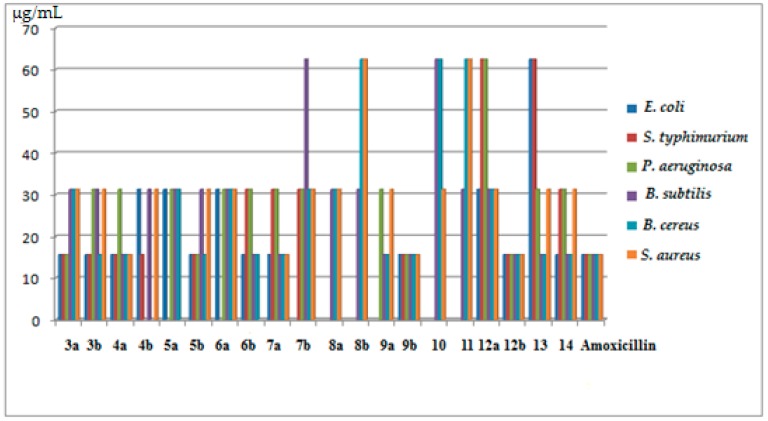
Comparison of antibacterial activity (MIC in µg/mL) of compounds **3a,b**–**14** and reference antibiotic (amoxicillin trihydrate).

The results of the in vitro antibacterial screening (inhibition zone in mm, MIC in µg/mL) of the novel thienopyridines (**3a,b**, and **4a,b**) and tetracyclic pyridothienoquinolines (**5a,b**–**14**) against Gram-negative bacteria (*Escherichia coli* 8739*, Salmonella typhimurium* 14028, *Pseudomonas aeruginosa* 27853) and Gram-positive bacteria (*Bacillus subtilis* 6633*, Bacillus cereus* 33018*, Staphylococcus aureus* 25923), represented in Table 1 and Figure 1, revealed that compounds **3a,b, 4a, 5b, 6b, 7a, 9b, 12b,** and **14** had significant activity compared with amoxicillin trihydrate. The most active of these compounds were the tetracyclic 2-(4-methylpiperazin-1-yl)acetamide derivative **9b** and 1-(thiophen-2-yl)methanimine derivative **12b**. They had an MIC = 15.63 µg/mL against the six bacterial strains, which equalled the effect of amoxicillin trihydrate. Moreover, 2-hydrazinylacetamide derivative **7a** revealed higher inhibition activity against Gram-positive strains, especially against *S*. *aureus*, with an inhibition zone of 19 mm and an MIC value of 15.63 µg/mL, the same as the reference drug. The 4-(4-methoxyphenyl)thieno[2,3-b]pyridine-2-carboxamide derivative **3a** and tetracyclic amine **5b** were mostly active against Gram-negative bacteria. In addition, amides **3b** and **4a**, chloroacetamide derivative **6b**, and pyrazolylamine derivative **14** showed variable activity against the different bacterial strains, ranging from potent to moderate. For example, **4a**, which had potent activity against *E. coli* and *S*. *aureus,* had only moderate activity against *P. aeruginosa*. Compounds **4b**, **5a**, **7a**, **6a, 9a, 12a,** and **13** possessed moderate inhibitory activity against some of the tested organisms. The remaining compounds (**8a,b, 10,** and **11**) were mostly inactive.

#### 2.2.2. Antimicrobial Resistance Activity against MRSA

The novel compounds which showed highest inhibitory activity against *Staphylococcus aureus* (**4a, 7a, 9b,** and **12b**) were selected to be screened for their inhibitory activity against methicillin-resistant *Staphylococcus aureus*, compared with gentamicin as a reference antibiotic. The results of inhibition zones in mm are presented in Table 2, revealed that Schiff base **12b** and hydrazide derivative **7a** were the most potent compounds against MRSA; their inhibition zones were 15 mm and 18 mm, respectively. The inhibition zone of gentamicin was 15 mm, so **12b** and **7a** had inhibition activity equipotent to gentamicin or exceeded it, respectively. Additionally, compounds **4a** and **9b** showed good activity against MRSA, with inhibition zones near to that of the reference.

#### 2.2.3. *Escherichia coli* DNA gyrase Inhibition Activity

Targeting of DNA gyrase with an inhibitor has been considered as new strategy for developing antimicrobial agents that can deal with antimicrobial resistance. Thus, the most active antibacterial compounds (**3a,b, 4a, 5b, 6a,b, 7a, 9b, 12b,** and **14**) were selected to evaluate their in vitro inhibition activity against DNA gyrase from *Escherichia coli*, compared with novobiocin as a reference inhibitor. The evaluation results, presented in Table 3, showed that thienopyridine carboxamides **3a** and **4a** were the most active among the tested compounds with IC_50_ = 2.26 and 3.69 µM, and were also more potent than novobiocin (IC_50_ = 4.17 µM). Moreover, carboxamide derivative **3b** and tetracyclic methanimine derivative **12b** showed promising inhibition activity (IC_50_ = 4.50 and 4.60 µM) near that of Novobiocin. Methylpiperazinyl derivative **9b** and chloroacetamide derivative **6b** showed good inhibition with IC_50_ = 5.78 and 5.95 µM, respectively. From the compounds’ IC_50_ values, it was clear that the presence of carboxamide, methanimine, methylpiperazinyl, and chloroacetamide moieties in their structure supported inhibition activity against DNA gyrase.

#### 2.2.4. Molecular Docking Studies

One of the most important strategies in structure-based drug design, is molecular docking, as it can give an idea of the binding modes of the novel molecules in the binding site of the suitable target, which is a key step in drug design [36,37]. In this research work, docking studies were performed to give insight into the mode of binding between the enzyme active binding site and the novel bioactive compound, in addition to possible interactions and the docking score.

To correlate the observed potencies and structure activity relationship (SAR) of our newly synthesized derivatives, which had thieno[2,3-b]pyridine or the fused tetrahydroquinoline system giving their possible binding modes and interactions within the active site of *E. coli* DNA gyrase B, docking simulation was carried out. The co-crystallized ligand novobiocin was re-docked in the active site of *E. coli* DNA gyrase B (PDB code: 1AJ6) [38,39] and exhibited and energy score of −6.30 kcal/mol, with a root-mean-square deviation (RMDS) value equal to 9.2. The most active 10 compounds were docked into the ATP-active site of *E. coli* DNA gyrase B using the 3D protein structure (PDB ID: 1AJ6). The docking results obtained for the investigated derivatives are recorded in Table 4.

**Figure 2:**
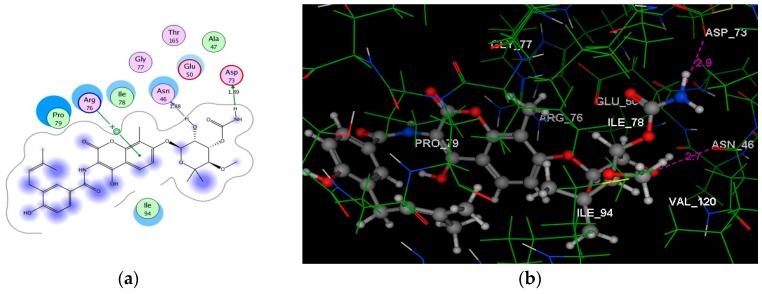
2D (**a**) and 3D (**b**) diagrams illustrating the binding patterns of the co-crystallized ligand novobiocin into the ATP-active pocket of *E. coli* DNA gyrase B (PDB code: 1AJ6).

Novobiocin consists of a coumarin core linked to an oxan-4-yl moiety. This ligand binds to *E. coli* DNA gyrase B kinase via formation of two hydrogen bonds, one between the hydroxyl proton of oxan-4yl moiety and the backbone of Asp46, and the other between the sidechain of Asp73 and the protons of NH_2_ of the carbamate group in novobiocin. Moreover, the coumarin scaffold establishes an arene–cation interaction with Arg76 in the ligand (Figure 2).

**Figure 3:**
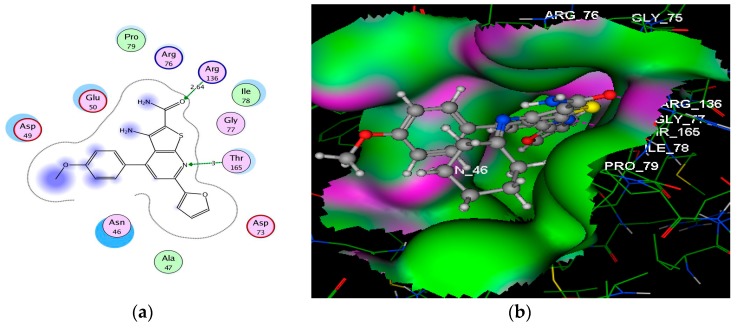
2D (**a**) and 3D (**b**) diagrams illustrating the binding patterns of compound **3a** into the ATP-active pocket of *E. coli* DNA gyrase B (PDB code: 1AJ6).

**Figure 4:**
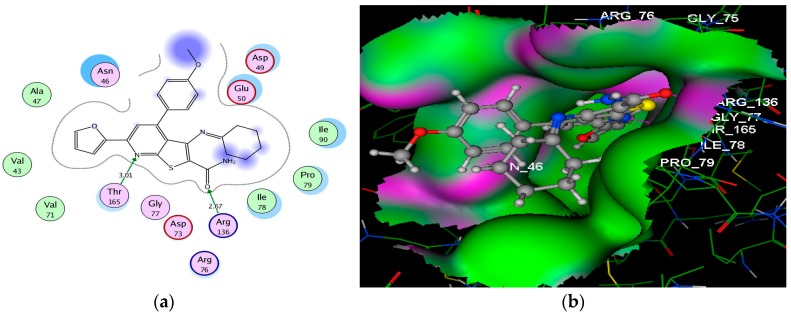
2D (**a**) and 3D (**b**) diagrams illustrating the binding patterns of compound **4a** into the ATP-active pocket of *E. coli* DNA gyrase B (PDB code: 1AJ6).

**Figure 5:**
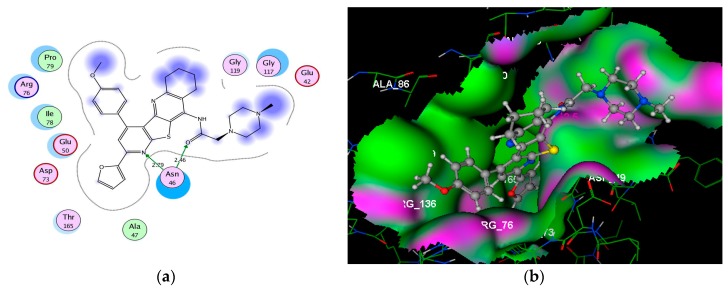
2D (**a**) and 3D (**b**) diagrams illustrating the binding patterns of compound **9b** into the ATP-active pocket of *E. coli* DNA gyrase B (PDB code: 1AJ6).

**Figure 6:**
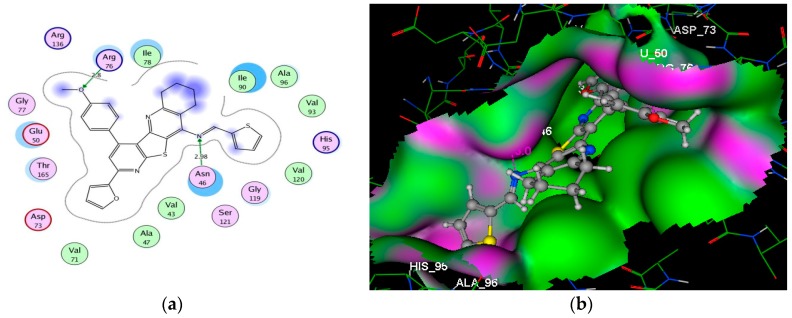
2D (**a**) and 3D (**b**) diagrams illustrating the binding patterns of compound **12b** into the ATP-active pocket of *E. coli* DNA gyrase B (PDB code: 1AJ6).

It was clear from the docking data that compounds **3a, 3b, 4a, 9b,** and **12b**, with the highest *E. coli* DNA gyrase B inhibitory activities, showed the best binding style. These thieno[2,3-b]pyridine derivatives exhibited hydrogen bonds between the nitrogen of the pyridine and the side chain of Thr165. Another hydrogen bond appeared between the oxygen of the carboxamide group and Arg136 (Figure 3, Figure 4, Figure 5 and Figure 6, respectively).

Fusion of the thieno[2,3-b]pyridine scaffold with a tetrahydroquinoline moiety maintained the improved potency in **9b** and **12b**, which could be attributed to the presence of the important Asn46 residue via two hydrogen bonds with the nitrogen of pyridine and oxygen of acetamide in 9b (distance: 2.79 and 2.46 Å) and one hydrogen bond with nitrogen of methanimine group in **12b** (distance: 2.98 Å). Additionally, the oxygen of the methoxy group in **12b** formed a favorable hydrogen bond with the side chain of Arg76 (distance: 2.80 Å). As show in Figure 5a,b and Figure 6a,b.

Finally, the docking results in Table 4, Figure 2, Figure 3, Figure 4, Figure 5 and Figure 6 of the screened compounds (**3a, 3b, 4a, 5b, 6a, 6b, 7a, 9b, 12b,** and **14**), and their *E. coli* DNA gyrase B inhibitory activities confirmed that the presence of carboxamide, acetamide, and methanimine groups led to enhancement of the inhibitory activities of these thieno[2,3-b]pyridine-based and tetrahydroquinoline-based compounds by forming significant H-bonds and other interactions inside the ATP-binding cavity.

## 3. Experimental

### 3.1. Chemistry

#### 3.1.1. General Information

Melting points were recorded in open glass capillary tubes using an Electro thermal IA9100 digital melting point apparatus and were uncorrected. Elemental microanalyses were carried out at the Microanalytical Unit in Cairo University, and were found to within ±0.5%. Infrared spectra were recorded on a Jasco FT/IR-6100, Fourier transform infrared spectrometer (Tokyo, Japan) at cm^−1^ scale using the KBr disc technique. ^1^H NMR and ^13^C NMR spectra were recorded on a Bruker High-Performance Digital FT-NMR Spectrometer Advance III (400/100 MHz, Billerica, MA, USA) in the presence of TMS as internal standard. The mass spectra were measured using a Model (ISQ LT) mass spectrometer using Thermo X-CALIBUR SOFTWARE. Follow ups of the reactions and checks of the purity of the compounds were made by TLC on silica gel aluminum sheets (Type 60, F 254, Merck, Darmstadt, Germany), and the spots were detected by exposure to a UV analysis lamp at λ 254/366 nm for a few seconds and by iodine vapor. The chemical names given for the prepared compounds were according to the IUPAC system. The starting 2-thioxo-1,2-dihydropyridine-3-carbonitriles **1a,b** were prepared by following the literature method [35].

#### 3.1.2. Synthesis of 2-((3-Cyanopyridin-2-yl)thio)acetamides 2a,b

A mixture of compounds **1a,b** (0.1 mol) and 2-chloroacetamide (9.35, 0.1 mol) in *N,N*-dimethylformamide (100 mL) containing anhydrous sodium carbonate (15 g) was heated at 80 °C with stirring for 3 h. The reaction mixture was poured into an ice–water mixture. The formed precipitate was collected by filtration, washed with water, dried, and recrystallized from DMF/H_2_O to produce compounds **2a,b**.

*2-((3-Cyano-6-(furan-2-yl)-4-(4-methoxyphenyl)pyridin-2-yl)thio)acetamide* (**2a**), pale yellow solid, (82% yield), m.p. 246 °C. IR (KBr, ν _max_ cm^−1^): 3407, 3195 (NH_2_), 2924, 2850 (CH), 2210 (CN), 1670 (C=O), 1601 (C=N); ^1^H-NMR (DMSO-d_6_, 400 MHz): δ = 3.85 (s, 3H,OCH_3_), 4.01 (s, 2H, SCH_2_), 6.78 (m, 1H, Ar-H), 7.14 (d, 2H, *J* = 8.7 Hz, Ar-H), 7.26 (s, 1H, Ar-H), 7.51 (s, 2H, NH_2_, D_2_O exchangeable), 7.69 (d, 2H, *J* = 8.7 Hz, Ar-H), 7.72 (d, 1H, *J* = 7.0 Hz, Ar-H), 7.99 (d, 1H, *J* = 7.0 Hz, Ar-H); MS *m*/*z* (%) 365 (M^+^, 39), 349 (9), 321 (100), 307 (11), 292 (10), 278(15), 100 (10). Anal. Calcd. For C_19_H_15_N_3_O_3_S (365.41): C, 62.45; H, 4.14; N, 11.50%. Found: C, 62.11; H, 4.51; N, 11.88%.

*2-((3-Cyano-6-(furan-2-yl)-4-(thiophen-2-yl)pyridin-2-yl)thio)acetamide* (**2b**), brown solid, (81% yield), m.p. 242 °C; IR (KBr, ν _max_ cm^−1^): 3378, 3196 (NH_2_), 2925 (CH), 2211 (CN), 1661 (C=O), 1601 (C=N); ^1^H-NMR (DMSO-d_6_, 400 MHz): δ = 4.00 (s, 2H, SCH_2_), 6.69-7.60 (m, 5H, Ar-H), 7.71 (s, 2H, NH_2_, D_2_O exchangeable), 7.93–8.03 (m, 2H, Ar-H); MS *m*/*z* (%) 341 (M^+^, 35), 325 (5), 299 (13), 297 (100), 269 (14), 251 (6), 108 (5), 58 (9), 44(64). Anal. Calcd. For C_16_H_11_N_3_O_2_S_2_ (341.40): C, 56.29; H, 3.25; N, 12.31%. Found: C, 56.58; H, 3.49; N, 12.52%.

#### 3.1.3. Synthesis of 3-Amino-thieno[2,3-b]pyridine-2-carboxamides 3a,b

Compounds **2a,b** (20 mmol) were added to a solution of 0.2 M MeONa in MeOH (40 mL), and the reaction mixture was refluxed for 4 h. The precipitate formed after cooling was isolated by filtration, washed with ethanol, dried, and recrystallized from ethanol to give products **3a,b**.

3-Amino-6-(furan-2-yl)-4-(4-methoxyphenyl)thieno[2,3-b]pyridine-2-carboxamide (3a), yellow solid, (74% yield), m.p. 220 °C; IR (KBr, ν _max_ cm^−1^): 3462, 3310, 3256 (NH), 2940 (CH), 1656 (C=O), 1592 (C=N); ^1^H-NMR (DMSO-d_6_, 400 MHz): δ = 3.86 (s, 3H, OCH_3_), 5.97 (s, 2H, NH_2_, D_2_O exchangeable), 6.71 (s, 1H, Ar-H), 7.14 (d, 2H, *J* = 7.6 Hz, Ar-H), 7.23 (m, 1H, Ar-H), 7.34 (d, 1H, *J* = 5.2 Hz, Ar-H), 7.50 (d, 2H, *J* = 8.4 Hz, Ar-H), 7.51 (s, 2H, NH_2_, D_2_O exchangeable), 7.91 (s, 1H, Ar-H); ^13^C-NMR (DMSO-d_6_, 100 MHz): δ = 55.80 (OCH_3_), 106.19, 111.35, 113.18, 114.71, 116.99, 129.65, 130.48, 131.35, 145.61, 146.20, 152.65, 154.18, 161.19, 162.20 (Ar–C), 167.30 (C=O); MS *m*/*z* (%) 365 (M^+^, 20), 321 (9), 323 (100), 296 (16), 266 (29), 238 (19), 191 (15), 44 (14). Anal. Calcd. For C_19_H_15_N_3_O_3_S (365.41): C, 62.45; H, 4.14; N, 11.50%. Found: C, 62.09; H, 4.47; N, 11.24%.

*3-Amino-6-(furan-2-yl)-4-(thiophen-2-yl)thieno[2,3-b]pyridine-2-carboxamide* (**3b**), yellow solid, (71% yield), m.p. 253°C; IR (KBr, ν _max_ cm^−1^): 3462, 3311, 3257 (NH), 2927 (CH), 1654 (C=O), 1594 (C=N); ^1^H-NMR (DMSO-d_6_, 400 MHz): δ = 6.20 (s, 2H, NH_2_, D_2_O exchangeable), 6.72 (m, 1H, Ar-H), 7.28-7.44 (m, 4H, Ar-H), 7.62 (s, 2H, NH_2_, D_2_O exchangeable), 7.89–7.93 (m, 2H, Ar-H); ^13^C-NMR (DMSO-d_6_, 100 MHz): δ = 107.04, 111.70, 113.27, 117.72, 121.22, 128.54, 129.63, 130.01, 136.68, 140.34, 145.84,146.02, 147.82, 152.30, 160.37 (Ar–C), 167.21 (C=O); MS *m*/*z* (%) 341 (M^+^, 91), 325 (14), 323 (100), 281 (20), 83 (4), 44 (12). Anal. Calcd. For C_16_H_11_N_3_O_2_S_2_ (341.40): C, 56.29; H, 3.25; N, 12.31%. Found: C, 55.96; H, 3.49; N, 12.66%.

#### 3.1.4. Synthesis of 3-(Cyclohexylideneamino)-thieno[2,3-b]pyridine-2-carboxamides 4a,b

A mixture of 3-amino-thieno[2,3-b]pyridine-2-carboxamides **3a,b** (10 mmol) and cyclohexanone (1.47 g, 15 mmol) in glacial acetic acid (20 mL) was refluxed for 2 h. The reaction mixture was concentrated, poured onto cold water, and the obtained solid was collected by filtration and recrystallized from acetone to give compounds **4a,b**.

*3-(Cyclohexylideneamino)-6-(furan-2-yl)-4-(4-methoxyphenyl)thieno[2,3-b]pyridine-2-carboxamide* (**4a**), yellow-green solid, (77% yield), m.p. 283–285 °C. IR (KBr, ν _max_ cm^−1^): 3418, 3264 (NH), 3045, 2928, 2856 (CH), 1645 (C=O), 1605 (C=N); ^1^H-NMR (DMSO-d_6_, 400 MHz): δ = 0.86–1.03 (m, 2H, CH_2_), 1.10–1.24 (m, 2H, CH_2_), 1.36–1.48 (m, 4H, 2CH_2_), 1.91–1.96 (m, 2H, CH_2_), 3.85 (s, 3H, OCH_3_), 6.73 (s, 1H, Ar-H), 7.16 (d, 2H, *J* = 10.4 Hz, Ar-H), 7.38 (d, 1H, *J* = 5.2 Hz, Ar-H),), 7.56 (d, 2H, *J* = 8.0 Hz, Ar-H), 7.62 (s, 2H, NH_2_, D_2_O exchangeable), 7.89–7.93 (m, 2H, Ar-H); ^13^C-NMR (DMSO-d_6_, 100 MHz): δ = 21.66 (CH_2_), 24.55 (2CH_2_), 36.15 (2CH_2_), 55.99 (OCH_3_), 105.42, 111.62, 113.24, 114.61, 116.40, 121.20, 128.73, 130.54, 142.76, 145.73, 147.62, 147.92, 152.64, 160.56, 161.33 (Ar–C, C=N), 162.65 (C=O); MS *m*/*z* (%) 445 (M^+^, 26), 416 (8), 403 (29), 402 (100), 389 (8), 349 (12), 319 (10), 277 (9), 54(7). Anal. Calcd. For C_25_H_23_N_3_O_3_S (445.54): C, 67.40; H, 5.20; N, 9.43%. Found: C, 67.72; H, 5.54; N, 9.77%.

*3-(Cyclohexylideneamino)-6-(furan-2-yl)-4-(thiophen-2-yl)thieno[2,3-b]pyridine-2-carboxamide* (**4b**), yellow-green solid, (73% yield), m.p. 320-322 °C. IR (KBr, ν _max_ cm^−1^): 3462, 3311 (NH), 2927, 2856 (CH), 1654 (C=O), 1594 (C=N); ^1^H-NMR (DMSO-d_6_, 400 MHz): δ = 0.96–1.11 (m, 4H, 2CH_2_), 1.42–1.53 (m, 4H, 2CH_2_), 1.98 (m, 2H, CH_2_), 6.72 (m, 1H, Ar-H), 7.32 -7.55 (m, 3H, Ar-H), 7.71 (s, 2H, NH_2_, D_2_O exchangeable), 7.92–7.97 (m, 3H, Ar-H);); MS *m*/*z* (%) 421 (M^+^, 100), 392 (5), 378 (43), 366 (4), 325 (30), 268 (11), 159 (13). Anal. Calcd. For C_22_H_19_N_3_O_2_S_2_ (421.53): C, 62.69; H, 4.54; N, 9.97%. Found: C, 62.36; H, 4.25; N, 10.25%.

#### 3.1.5. Synthesis of 6,7,8,9-Tetrahydropyrido[3′,2′:4,5]thieno[3,2-b]quinolin-10-amines 5a,b

A solution of compounds **4a,b** (10 mmol) in phosphorous oxychloride (25 mL) was refluxed for 3 h. After cooling, the reaction mixture was poured into an ice–water mixture and aqueous 10% NaOH solution was added to pH 7. The precipitate formed was isolated by filtration, washed with water, and recrystallized from ethanol to give free amines **5a,b**.

*2-(Furan-2-yl)-4-(4-methoxyphenyl)-6,7,8,9-tetrahydropyrido[3′,2′:4,5]thieno[3,2-b]quinolin-10-amine* (**5a**), beige solid, (68% yield), m.p. 231 °C. IR (KBr, ν _max_ cm^−1^): 3338, 3216 (NH), 2927(CH), 1601 (C=N); ^1^H-NMR (CDCl_3_, 400 MHz): δ = 1.83-1.89 (m, 4H, 2CH_2_), 2.60 (t, 2H, CH_2_), 2.74 (t, 2H, CH_2_), 3.93 (s, 3H, OCH_3_), 5.53 (br.s, 2H, NH_2_, D_2_O exchangeable), 6.59-7.73 (m, 8H, Ar-H); ^13^C-NMR (DMSO-d_6_, 100 MHz): δ = 22.78 (CH_2_), 22.98 (CH_2_), 23.86 (CH_2_), 33.20 (CH_2_), 55.67 (OCH_3_), 110.64, 112.97, 113.09, 117.21, 126.55, 129.54, 132.13, 137.70, 145.24, 147.40, 149.18, 152.70, 157.13, 160.15 (Ar–C); MS *m*/*z* (%) 427 (M^+^, 93), 426 (100), 412 (9), 498 (13), 484 (8), 482(18), 368 (4), 355 (5). Anal. Calcd. For C_25_H_21_N_3_O_2_S (427.52): C, 70.24; H, 4.95; N, 9.83%. Found: C, 70.56; H, 5.22; N, 9.56%.

*2-(Furan-2-yl)-4-(thiophen-2-yl)-6,7,8,9-tetrahydropyrido[3′,2′:4,5]thieno[3,2-b]quinolin-10-amine* (**5b**), beige solid, (65% yield), m.p. 248 °C. IR (KBr, ν _max_ cm^−1^): 3319, 3214 (NH), 2929, 2860 (CH), 1611 (C=N); ^1^H-NMR (DMSO-d_6_, 400 MHz): δ = 1.82 (s, 4H, 2CH_2_), 2.58 (t, 2H, CH_2_), 2.76 (t, 2H, CH_2_), 6.21 (s, 2H, NH_2_, D_2_O exchangeable), 6.74–8.26 (m, 7H, Ar-H); ^13^C-NMR (DMSO-d_6_, 100 MHz): δ = 22.75 (CH_2_), 22.99 (CH_2_), 23.86 (CH_2_), 33.09 (CH_2_), 110.81, 113.14, 113.23, 113.99, 116.60, 123.62, 127.59, 129.05, 132.53, 138.15, 140.54, 145.40, 147.25, 147.46, 152.67, 154.37, 163.17 (Ar–C); MS *m*/*z* (%) 403 (M^+^, 60), 402 (100), 387 (15), 374 (22), 360 (21), 347 (14), 253 (15), 225 (16), 198 (20). Anal. Calcd. For C_22_H_17_N_3_OS_2_ (403.52): C, 65.48; H, 4.25; N, 10.41%. Found: C, 65.14; H, 4.59; N, 10.12%.

#### 3.1.6. Synthesis of Chloroacetamide Derivatives 6a,b

Chloroacetyl chloride (2.26 g, 20 mmol) in 1,4-dioxane (10 mL) was added to a cold solution of the amines **5a,b** (10 mmol) in 1,4-dioxane (100 mL) containing a few drops of triethylamine at 5–10 °C, dropwise with stirring. After addition, the stirring was continued at room temperature for 8 h. The solvent was evaporated under vacuum and the residue was treated with boiled ethanol. The solid that formed was filtered off and recrystallized from DMF/H_2_O to give compounds **6a,b**.

*2-Chloro-N-(2-(furan-2-yl)-4-(4-methoxyphenyl)-6,7,8,9-tetrahydropyrido[3′,2′:4,5]thieno[3,2-b]quinolin-10-yl)acetamide* (**6a**), brown solid, (69% yield), m.p. 342–343 °C. IR (KBr, ν _max_ cm^−1^): 3260 (NH), 3007, 2934, 2857 (CH), 1667 (C=O), 1601 (C=N); ^1^H-NMR (DMSO-d_6_, 400 MHz): δ = 1.80 (m, 4H, 2CH_2_), 2.76 (t, 4H, 2CH_2_), 3.87 (s, 3H, OCH_3_), 4.45 (s, 2H, CH_2_Cl), 6.74–7.79 (m, 8H, Ar-H), 10.51 (s, 1H, NH, D_2_O exchangeable); MS *m*/*z* (%) 506 (M^+^+2, 16), 504 (M^+^, 50), 452 (9), 426 (100), 411 (18), 383 (17), 354 (49), 329 (11), 257 (10), 198 (33), 77 (54). Anal. Calcd. For C_27_H_22_ClN_3_O_3_S (504.00): C, 64.34; H, 4.40; N, 8.34%. Found: C, 64.11; H, 4.12; N, 8.58%.

*2-Chloro-N-(2-(furan-2-yl)-4-(thiophen-2-yl)-6,7,8,9-tetrahydropyrido[3′,2′:4,5]thieno[3,2-b]quinolin-10-yl)acetamide* (**6b**), brown solid, (71% yield), m.p. 338 °C. IR (KBr, ν _max_ cm^−1^): 3247 (NH), 3011, 2938, 2855 (CH), 1659 (C=O), 1589 (C=N); ^1^H-NMR (DMSO-d_6_, 400 MHz): δ = 1.80–1.85 (m, 4H, 2CH_2_), 2.77 (s, 2H, CH_2_), 2.92 (s, 2H, CH_2_), 4.46 (s, 2H, CH_2_Cl), 6.73–8.14 (m, 7H, Ar-H), 10.54 (s, 1H, NH, D_2_O exchangeable); ^13^C-NMR (DMSO-d_6_, 100 MHz): δ = 22.21 (CH_2_), 22.71 (CH_2_), 24.71 (CH_2_), 32.92 (CH_2_), 43.13 (CH_2_Cl), 111.60, 113.29, 114.90, 122.34, 127.01, 129.43, 130.01, 134.44, 140.54, 145.84, 146.02, 147.82, 152.34,160.37, 162.49, 163.42 (Ar–C), 167.21 (C=O); MS *m*/*z* (%) 482 (M^+^+2, 18), 480 (M^+^, 53), 482 (M^+^+2, 18), 479 (100), 444 (12), 430 (4), 402 (59), 387 (28), 77(38). Anal. Calcd. For C_24_H_18_ClN_3_O_2_S_2_ (480.00): C, 60.06; H, 3.78; N, 8.75%. Found: C, 60.38; H, 4.09; N, 8.99%.

#### 3.1.7. Synthesis of Hydrazinylacetamide Derivatives 7a,b

A mixture of chloroacetamide derivatives **6a,b** (5 mmol) and hydrazine hydrate 99% (2 mL, excess) in absolute ethanol (100 mL) was refluxed for 12 h. The reaction mixture was then evaporated to dryness under reduced pressure and the residue was treated with cold water. The obtained solid was collected by filtration and recrystallized from EtOH/H_2_O to give compounds **7a,b**.

*N-(2-(furan-2-yl)-4-(4-methoxyphenyl)-6,7,8,9-tetrahydropyrido[3′,2′:4,5]thieno[3,2-b]quinolin-10-yl)-2-hydrazinylacetamide* (**7a**), pale yellow, (78% yield), m.p. 278 °C. IR (KBr, ν _max_ cm^−1^): 3419, 3259 (NH), 2925, 2863 (CH), 1654 (C=O), 1599 (C=N); ^1^H-NMR (DMSO-d_6_, 400 MHz): δ = 1.74 (m, 4H, 2CH_2_), 2.19 (s, 2H, NH_2_, D_2_O exchangeable), 2.55–2.73 (m, 4H, 2CH_2_), 3.84 (s, 2H, CH_2_N), 3.87 (s, 3H, OCH_3_), 6.13 (s, 1H, NH, D_2_O exchangeable), 6.72–7.93 (m, 8H, Ar-H), 10.23 (s, 1H, NH, D_2_O exchangeable); MS *m*/*z* (%) 499 (M^+^, 24), 468 (14), 426 (100), 411 (16), 392 (17), 376 (15), 361 (17), 347 (14), 325 (11), 319 (10), 283 (13), 88 (10), 58 (12), 43 (11). Anal. Calcd. For C_27_H_25_N_5_O_3_S (499.59): C, 64.91; H, 5.04; N, 14.02%. Found: C, 64.67; H, 5.32; N, 13.78%.

*N-(2-(furan-2-yl)-4-(thiophen-2-yl)-6,7,8,9-tetrahydropyrido[3′,2′:4,5]thieno[3,2-b]quinolin-10-yl)-2-hydrazinylacetamide* (**7b**), pale yellow, (75% yield), m.p. 306 °C. IR (KBr, ν _max_ cm^−1^):): 3422, 3262 (NH), 2935 (CH), 1656 (C=O), 1591 (C=N); ^1^H-NMR (DMSO-d_6_, 400 MHz): δ = 1.85 (m, 4H, 2CH_2_), 2.20 (s, 2H, NH_2_, D_2_O exchangeable), 2.78 (t, 2H, CH_2_), 2.91 (t, 2H, CH_2_), 3.67 (s, 2H, CH_2_N), 5.32 (s, 1H, NH, D_2_O exchangeable), 6.75–8.16 (m, 7H, Ar-H), 10.22 (s, 1H, NH, D_2_O exchangeable); MS *m*/*z* (%) 475 (M^+^, 28), 461 (16), 446 (100), 374 (10), 348 (12), 319 (14), 291 (13), 73 (9), 43 (10). Anal. Calcd. For C_24_H_21_N_5_O_2_S_2_ (475.59): C, 60.61; H, 4.45; N, 14.73%. Found: C, 60.88; H, 4.69; N, 14.98%.

#### 3.1.8. Synthesis of Arylidene Derivatives 8a,b

A mixture of compounds **7a,b** (1 mmol) and the appropriate aldehyde (1 mmol) in glacial acetic acid (20 mL) was refluxed for 8 h. After reaction completion, the reaction mixture was concentrated and poured into cold water. The formed solid was collected by filtration, washed with water, and recrystallized from ethanol to give **8a,b**.

2-(2-(3,4-Dimethoxybenzylidene)hydrazinyl)-*N*-(2-(furan-2-yl)-4-(4-methoxyphenyl)-6,7,8,9-tetrahydropyrido[3′,2′:4,5]thieno[3,2-b]quinolin-10-yl)acetamide (**8a**), brown solid, (66% yield), m.p. 187 °C. IR (KBr, ν _max_ cm^−1^): 3429 (NH), 2922, 2856 (CH), 1647 (C=O), 1589 (C=N); ^1^H-NMR (DMSO-d_6_, 400 MHz): δ = 1.76–1.93 (m, 4H, 2CH_2_), 2.50–2.73 (m, 4H, 2CH_2_), 3.58 (s, 2H, NCH_2_), 3.85 (s, 9H, 3OCH_3_), 6.22 (br.s, 1H, NH, D_2_O exchangeable), 6.72–7.92 (m, 11H, Ar-H), 8.18 (s, 1H, CH=N), 10.11 (s, 1H, NH, D_2_O exchangeable); ^13^C-NMR (DMSO-d_6_, 100 MHz): δ = 22.32 (CH_2_), 22.73 (CH_2_), 23.80 (CH_2_), 32.92 (CH_2_), 47.54 (CH_2_N), 55.69, 55.95, 56.33 (3OCH_3_), 109.79, 111.70, 113.02, 113.29, 113.69, 114.20, 117.21, 117.37, 123.37, 126.63, 129.16, 129.51, 130.08, 132.32, 145.28, 145.58, 147.54, 147.84, 148.12, 149.61, 152.76, 152.94, 154.64, 160.19, 162.51 (Ar–C, CH=N), 168.86 (C=O); MS *m*/*z* (%) 647 (M^+^, 22), 591 (2), 574(100), 425(10), 355(2). Anal. Calcd. For C_36_H_33_N_5_O_5_S (647.75): C, 66.75; H, 5.14; N, 10.81%. Found: C, 66.45; H, 4.88; N, 11.12%.

*N*-(2-(furan-2-yl)-4-(4-methoxyphenyl)-6,7,8,9-tetrahydropyrido[3′,2′:4,5]thieno[3,2-b]quinolin-10-yl)-2-(2-(4-methylbenzylidene)hydrazinyl)acetamide (**8b**), brown solid, (64% yield), m.p. 122 °C. IR (KBr, ν _max_ cm^−^1): 3433 (NH), 2932, 2856 (CH), 1662 (C=O), 1608 (C=N); ^1^H-NMR (DMSO-d_6_, 400 MHz): δ = 1.78–1.94 (m, 4H, 2CH_2_), 2.37 (s, 3H, CH_3_), 2.51–2.75 (m, 4H, 2CH_2_), 3.64 (s, 2H, NCH_2_), 3.86 (s, 3H, OCH_3_), 4.45 (s, 1H, NH, D_2_O exchangeable), 6.78–8.04 (m, 12H, Ar-H), 8.67 (s, 1H, CH=N), 11.20 (s, 1H, NH, D_2_O exchangeable); MS *m*/*z* (%) 601 (M^+^, 8), 533 (12), 520 (12), 503 (41), 427 (17), 309 (100), 133 (10), 119(33), 68 (17). Anal. Calcd. For C_35_H_31_N_5_O_3_S (601.73): C, 69.86; H, 5.19; N, 11.64%. Found: C, 69.49; H, 4.88; N, 11.31%.

#### 3.1.9. Synthesis of Amine Acetamide Derivatives 9a,b

A mixture of chloroacetamide derivative **6a** (0.50 g, 1 mmol) and the appropriate amine (1 mmol) in *N,N*-dimethylformamide (10 mL) was refluxed for 12 h. After reaction completion, the reaction mixture was poured into an ice–water mixture. The obtained solid was collected by filtration and recrystallized from acetone to give compounds **9a,b**.

*N*-*(2-(furan-2-yl)-4-(4-methoxyphenyl)-6,7,8,9-tetrahydropyrido[3′,2′:4,5]thieno[3,2-b]quinolin-10-yl)-2-morpholinoacetamide* (**9a**), yellow solid, (69% yield), m.p. 143 °C. IR (KBr, ν _max_ cm^−1^):): 3432 (NH), 2924(CH), 1651 (C=O), 1601 (C=N); ^1^H-NMR (CDCl_3_, 400 MHz): δ = 1.73–1.75 (m, 4H, 2CH_2_), 2.47–2.69 (m, 4H, 2CH_2_N), 2.80 (s, 2H, CH_2_), 2.87 (s, 2H, CH_2_), 3.31 (s, 2H, CH_2_N), 3.64 (s, 4H, 2CH_2_O), 3.93 (s, 3H, OCH_3_), 6.48–7.93 (m, 8H, Ar-H), 9.08 (s, H, NH, D_2_O exchangeable); ^13^C-NMR (CDCl3, 100 MHz): δ = 22.53 (CH_2_), 22.67 (CH_2_), 23.27 (CH_2_), 32.95 (CH_2_), 49.38 (CH_2_N), 55.43 (OCH_3_), 62.12 (2CH_2_N), 67.11 (2CH_2_O), 110.34, 111.62, 112.46, 112.86, 113.25, 114.12, 117.48, 118.27, 124.54, 129.42, 130.12, 131.56, 143.96, 144.53, 148.64, 153.19, 160.17, 160.97 (Ar–C); MS *m*/*z* (%) 554 (M^+^, 25), 498 (10), 454 (11), 426 (100), 380 (9), 100 (12), 86(14). Anal. Calcd. For C_31_H_30_N_4_O_4_S (554.67): C, 67.13; H, 5.45; N, 10.10%. Found: C, 67.42; H, 5.73; N, 9.88%.

*N*-(2-(furan-2-yl)-4-(4-methoxyphenyl)-6,7,8,9-tetrahydropyrido[3′,2′:4,5]thieno[3,2-b]quinolin-10-yl)-2-(4-methylpiperazin-1-yl)acetamide (9b), yellow solid, (71% yield), m.p. 158-159°C. IR (KBr, ν max cm−1): 3422 (NH), 2923, 2851 (CH), 1654 (C=O), 1602 (C=N); 1H-NMR (DMSO-d6, 400 MHz): δ = 1.74 (s, 4H, 2CH2), 2.25 (s, 3H, NCH_3_), 2.51–2.56 (m, 8H, 4 NCH_2_), 2.73 (s, 2H, CH_2_), 2.88 (s, 2H, CH_2_), 3.28 (s, 2H, NCH_2_), 3.85 (s, 3H, OCH_3_), 6.71–7.95 (m, 8H, Ar-H), 9.65 (s, 1H, NH, D_2_O exchangeable); MS *m*/*z* (%) 567 (M^+^, 33), 430 (12), 427 (100), 393 (11), 379 (8), 365 (9). Anal. Calcd. For C_32_H_33_N_5_O_3_S (567.71): C, 67.70; H, 5.86; N, 12.34%. Found: C, 67.98; H, 5.54; N, 12.03%.

#### 3.1.10. Synthesis of *N*-(2-(Furan-2-yl)-4-(4-methoxyphenyl)-6,7,8,9-tetrahydropyrido[3′,2′:4,5]thieno [3,2-b]quinolin-10-yl)benzenesulfonamide 10

A mixture of the amine **5a** (0.43 g, 1 mmol) and benzenesulfonyl chloride (0.18 g, 1 mmol) in pyridine (10 mL) was refluxed for 12 h. After cooling, the reaction mixture was poured into an ice–water mixture. The precipitate formed was isolated by filtration, washed with water, dried, and recrystallized from acetone to give sulfonamide derivative **10**, a grey solid, (74% yield), m.p. 303–304 °C. IR (KBr, ν _max_ cm^−1^): 3424, (NH), 3088, 2927, 2852 (CH), 1600 (C=N), 1335, 1170 (SO_2_); ^1^H-NMR (DMSO-d_6_, 400 MHz): δ = 1.80 (s, 4H, 2CH_2_), 2.71 (s, 2H, CH_2_), 3.09 (s, 2H, CH_2_), 3.90 (s, 3H, OCH_3_), 6.77–7.99 (m, 13H, Ar-H), 11.04 (s, 1H, NH, D_2_O exchangeable); MS *m*/*z* (%) 567 (M^+^, 15), 490 (6), 474 (14), 488 (5), 474 (14), 460 (21), 446 (47), 310 (100), 77 (4). Anal. Calcd. For C_31_H_25_N_3_O_4_S_2_ (567.68): C, 65.59; H, 4.44; N, 7.40%. Found: C, 65.33; H, 4.12; N, 7.12%.

#### 3.1.11. Synthesis of 1-(2-(Furan-2-yl)-4-(4-methoxyphenyl)-6,7,8,9-tetrahydropyrido[3′,2′:4,5]thieno [3,2-b]quinolin-10-yl)-3-phenylurea 11

A mixture of amine **5a** (0.43 g, 1 mmol) and phenyl isocyanate (0.12 g, 1 mmol) in absolute ethanol (20 mL) containing a few drops of glacial acetic acid was refluxed for 8 h. The reaction mixture was then evaporated to dryness under reduced pressure and the residue was treated with cold water. The formed solid was collected by filtration and recrystallized from 2-propanol to give phenylurea derivative **11**, a pale yellow solid, (77% yield), m.p. 118–120 °C. IR (KBr, ν _max_ cm^−1^): 3427, 3294 (NH), 2924, 2857 (CH), 1636 (C=O); ^1^H-NMR (DMSO-d_6_, 400 MHz): δ = 1.78-1.79 (m, 4H, 2CH_2_), 2.55–2.62 (m, 4H, 2CH_2_), 3.86 (s, 3H, OCH_3_), 6.75–7.95 (m, 13H, Ar-H), 9.08, 10.15 (2s, 2H, 2NH, D_2_O exchangeable); MS *m*/*z* (%) 546 (M^+^, 31), 468 (12), 426 (45), 411 (12), 135 (10), 92 (40), 76 (40), 57 (100). Anal. Calcd. For C_32_H_26_N_4_O_3_S (546.65): C, 70.31; H, 4.79; N, 10.25%. Found: C, 70.60; H, 4.52; N, 10.69%.

#### 3.1.12. Synthesis of Schiff Bases 12a,b

A mixture of amine **5a** (0.43 g, 1 mmol) and the appropriate aldehyde (1 mmol) in glacial acetic acid (20 mL) was refluxed for 12 h. After reaction completion, the reaction mixture was poured into an ice–water mixture. The obtained solid was collected by filtration, washed with water, and recrystallized from ethanol to give compounds **12a,b**.

4-(((2-(furan-2-yl)-4-(4-methoxyphenyl)-6,7,8,9-tetrahydropyrido[3′,2′:4,5]thieno[3,2-b]quinolin-10-yl)imino)methyl)-N,N-dimethylaniline (**12a**), orange solid, (74% yield), m.p. 149°C. IR (KBr, ν _max_ cm^−1^): 2921 (CH), 1604 (C=N); ^1^H-NMR (DMSO-d_6_, 400 MHz): δ = 1.75 (s, 4H, 2CH_2_), 2.57–2.84 (m, 4H, 2CH_2_), 2.93 (s, 6H, N(CH_3_)_2_), 3.84 (s, 3H, OCH_3_), 6.67–7.90 (m, 12H, Ar-H), 8.30 (s, 1H, CH=N)); ^13^C-NMR (DMSO-d_6_, 100 MHz): δ = 22.68 (CH_2_), 22.82 (CH_2_), 23.68 (CH_2_), 33.40 (CH_2_), 40.72 (2NCH_3_) 55.45 (OCH_3_), 109.50, 111.51, 112.19, 112.96, 113.12, 113.55, 114.21, 117.23, 125.21, 129.61, 130.94, 131.30, 131.69, 132.03, 147.73, 147.95, 149.71, 154.65, 157.65, 162.49, 162.62 (Ar–C, CH=N); MS *m*/*z* (%) 558 (M^+^, 29), 437(28), 425 (40), 407(19) 208 (100), 134 (23). Anal. Calcd. For C_34_H_30_N_4_O_2_S (558.21): C, 73.09; H, 5.41; N, 10.03%. Found: C, 73.38; H, 5.79; N, 9.68%.

N-(2-(furan-2-yl)-4-(4-methoxyphenyl)-6,7,8,9-tetrahydropyrido[3′,2′:4,5]thieno[3,2-b]quinolin-10-yl)-1-(thiophen-2-yl)methanimine (**12b**), pale yellow solid, (76% yield), m.p. 114 °C. IR (KBr, ν _max_ cm^−1^): 3095, 2921, 2854 (CH), 1611 (C=N); ^1^H-NMR (DMSO-d_6_, 400 MHz): δ = 1.72–1.78 (m, 4H, 2CH_2_), 2.61-2.73 (m, 4H, 2CH_2_), 3.86 (s, 3H, OCH_3_), 6.70-7.99 (m, 11H, Ar-H), 8.84 (s, 1H, CH=N)); MS *m*/*z* (%) 521 (M^+^, 84), 520 (100), 438 (4), 426 (68), 425 (11), 412 (5), 411(7), 398 (7), 110 (4), 96(7), 83 (5). Anal. Calcd. For C_30_H_23_N_3_O_2_S_2_ (521.65): C, 69.07; H, 4.44; N, 8.06%. Found: C, 69.35; H, 4.73; N, 7.74%.

#### 3.1.13. Synthesis of Oxobutanamide Derivative 13

A mixture of amine **5b** (0.40 g, 1 mmol) and ethyl acetoacetate (0.13 g,1 mmol) in dimethylsulfoxide (20 mL) containing anhydrous sodium carbonate (0.4 g) was heated at 80 °C with stirring for 8 h. The reaction mixture was poured into an ice–water mixture and left in the refrigerator overnight. The obtained solid was collected by filtration, washed with water and recrystallized from ethanol to give *N*-(2-(furan-2-yl)-4-(thiophen-2-yl)-6,7,8,9-tetrahydropyrido[3′,2′:4,5]thieno[3,2-*b*]quinolin-10-yl)-3-oxobutanamide **13**, a pale yellow solid, (76% yield), m.p. 217–218 °C. IR (KBr, ν _max_ cm^−1^): 3427 (NH), 2921, 2852 (CH), 1716, 1643 (C=O); ^1^H-NMR (CDCl_3_, 400 MHz): δ = 1.90 (s, 7H, 2CH_2_, CH_3_), 2.62 (t, 2H, CH_2_), 2.91 (t, 2H, CH_2_), 4.27 (s, 2H, CH_2_C=O), 6.60 (s, 1H, Ar-H), 7.22 (s, 2H, Ar-H), 7.29 (s, 1H, NH, D_2_O exchangeable), 7.54 (s, d, 1H, *J* = 6 Hz, Ar-H), 7.62 (d, 1H, *J* =7.2 Hz, Ar-H), 7.90 (s, 1H, Ar-H), 8.19 (d, 1H, *J* = 3.6 Hz, Ar-H); MS *m*/*z* (%) 487 (M^+^, 22), 402 (100), 405 (13), 375 (25), 362 (8), 347 (4), 304 (11), 295 (4), 280 (5), 83 (4). Anal. Calcd. For C_26_H_21_N_3_O_3_S_2_ (487.59): C, 64.05; H, 4.34; N, 8.62%. Found: C, 64.33; H, 4.62; N, 8.94%.

#### 3.1.14. Synthesis of *N*-(5-methyl-4H-pyrazol-3-yl)-10-amine Derivative 14

A mixture of **13** (0.49 g, 1 mmol) and hydrazine hydrate 99% (1 mL, excess) in *N,N*-dimethylformamide (15 mL) was refluxed for 12 h. After reaction completion, the reaction mixture was poured into an ice–water mixture. The obtained solid was collected by filtration, washed with water, and recrystallized from acetone to give 2-(furan-2-yl)-*N*-(5-methyl-4*H*-pyrazol-3-yl) -4-(thiophen-2-yl)-6,7,8,9-tetrahydropyrido[3′,2′:4,5]thieno[3,2-*b*]quinolin-10-amine **14**, a brown solid, (68% yield), m.p. 274°C. IR (KBr, ν _max_ cm^−1^): 3326 (NH), 2925 (CH), 1637, 1597(C=N); ^1^H-NMR (DMSO-d_6_, 400 MHz): δ = 1.79–1.81 (m, 4H, 2CH_2_), 2.51–2.77 (m, 4H, 2CH_2_), 2.89 (s, 3H, CH_3_), 3.00 (s, 2H, pyrazol-CH_2_), 6.73–8.26 (m, 7H, Ar-H), 8.53 (s, 1H, NH, D_2_O exchangeable); MS *m/z* (%) 483 (M^+^, 26), 447 (22), 403 (100), 401(45), 386 (57), 292 (25), 278 (42), 263 (13), 180 (37), 83 (33). Anal. Calcd. For C_26_H_21_N_5_OS_2_ (483.61): C, 64.57; H, 4.38; N, 14.48%. Found: C, 64.86; H, 4.66; N, 14.20%.

### 3.2. Antimicrobial Activity

#### 3.2.1. Antibacterial Assay

The clinical control strains of Gram-negative bacteria (*Escherichia coli* 8739*, Salmonella typhimurium* 14028, *Pseudomonas aeruginosa* 27853) and Gram-positive bacteria (*Bacillus subtilis* 6633*, Bacillus cereus* 33018*, Staphylococcus aureus* 25923), were obtained from microbial inoculums by subculturing microorganisms into nutrient broth (NB) at 37 °C for 18 h, and were adjusted to 0.125 A° at 625 nm (equivalent to a McFarland 0.5) in 2-fold NB. A 100 μL measure of NB containing each test microorganism was added to the sterile Petri dishes. Antibacterial testing was carried out on all compounds (**3a,b**–**14**) using the agar disc-diffusion method [40]. Sterile nutrients were inoculated with 100 μL cell suspension of the chosen test microorganism and poured into Petri dishes (20 cm diameter). Filter paper discs (Whatman, No.3, diameter 5 mm) were loaded with 10 μL containing 100 μg of the tested compounds, as well as the standard drug. Composite compound disks were placed on the surface of inoculated agar plates and kept at low temperatures before incubation in order to support the swelling and diffusion of the microbial growth. The plates were incubated at 37 °C in the incubator. Experiments were performed in duplicate and the diameters of inhibition zones were measured after 24 h. A diameter of inhibition zone (DIZ) assay was performed to evaluate the antimicrobial potential of the compounds against the tested organisms compared with reference antibiotics. All measurements were done in DMSO as a solvent, which has zero inhibition activity [41], compared with amoxicillin trihydrate (inhibition zones against six strains = 20, 21, 18, 17, 19, and 19 mm, respectively) and gentamycin (inhibition zone against MRSA = 15 mm) as reference antibiotics. Minimum inhibition concentration (MIC) values of the active compounds and the standard drugs were determined using a 7-fold dilution (125, 62.5, 31.25, 15.63, 7.81, 3.91, and 1.95 μg/mL) procedure [42]. Tubes showing no growth of the test organism were counted and the minimum dilution of the sample which caused the inhibition of growth of the tested organism (MIC) was specified, compared with amoxicillin trihydrate as a positive control (MIC value for the six strains = 15.63 μg/mL), as shown in Table 1 and Table 2.

#### 3.2.2. Escherichia coli DNA Gyrase Supercoiling Inhibition Assay

The assay for determining IC_50_ values (TopoGEN) was performed on black streptavidin-coated 96 well micro-titer plates (Thermo Scientific Pierce). The plate was hydrated with the wash buffer supplied (137 mM NaCl, 20 mM Tris-HCl (pH 7.6), 0.05% (v/v) Tween 20, 0.01% (w/v) BSA). Biotinylated oligonucleotide in wash buffer was immobilized onto the wells. The excess oligonucleotide was washed off and the enzyme assay was carried out in the wells (5 min). The final reaction volume was 30 µL in buffer (24 mM KCl; 35 mM TrisHCl (pH 7.5); 2 mM DTT; 4 mM MgCl_2_; 1.8 mM spermidine; 1 mM ATP; 0.1 mg/mL albumin; and 6.5% (w/v) glycerol), in addition to 1.5 U of DNA gyrase from *E. coli*, 0.008% Tween 20, 0.75 µg of relaxed pBR322 plasmid, and 3 µL of inhibitor solution in 10% DMSO. Reactions were incubated for 30 min at 37 °C and, after addition of the TF buffer (50 mM NaOAc (pH 5.0), 50 mM MgCl_2_ and 50 mM NaCl), which terminated the enzymatic reaction, for another 30 min at room temperature to allow triplex formation (biotin–oligonucleotide–plasmid). The unbound plasmid was washed off using TF buffer, and a solution of ethidium bromide stain in T10 buffer (10 mM Tris HCl (pH 8.0) and 1 mM EDTA) was added. After mixing, the fluorescence (excitation, 485 nm; emission, 535 nm) was read using a BioTek’s Synergy H4 microplate reader. Screening was investigated at inhibitor concentrations of 100 µM and 10 µM. For the most effective 10 compounds, IC_50_ was determined with 7 concentrations of the inhibitors. Calculation of IC_50_ values by using GraphPad Prism software and the concentration of inhibitor have been represented where the residual activity of the enzyme was 50% in three independent measurements; the final result is given as their average value. Novobiocin (IC_50_ = 4.17 µM for *E. coli* DNA gyrase) was used as a reference drug [43,44]. *E. coli* DNA gyrase supercoiling inhibition (IC_50_ µM) of the tested compounds (**3a,b, 4a, 5b, 6a,b, 7a, 9b, 12b,** and **14**) and reference antibiotics (novobiocin) are listed in Table 3.

#### 3.2.3. Molecular Docking

The molecular docking simulation study was performed using Molecular Operating Environment (MOE^®^) 2008.10 software [45]. The crystal structures of *E. coli* topoisomerase II DNA gyrase B complexed with their ligands novobiocin (PDB code: 1AJ6) [37,38] was retrieved from the Protein Data Bank. Initially, the co-crystallized ligands were re-docked into the assigned active *E. coli* DNA gyrase B enzyme to evaluate a root-mean-square deviation value. The molecular docking procedure was done for the newly synthesized compounds (**3a,b, 4a, 5b, 6a,b, 7a, 9b, 12b,** and **14**) into the ATP-binding site of *E. coli* DNA gyrase B (PDB code: 1AJ6) following the previously reported method [45].

## 4. Conclusions

Novel bicyclic thieno[2,3-b]pyridines and tetracyclic pyridothienoquinolines (**3a,b–14**) were synthesized and evaluated for their in vitro antimicrobial activity against six bacterial strains. Whereas, compounds **3a,b**, **4a**, **5b**, **6b**, **7a**, **9b**, **12b,** and **14** all showed significant activity, compounds **9a** and **12b** were the most active of them, with MIC = 15.63 µg /mL, equal to that of the reference for all tested organisms. Furthermore, the active synthesized compounds (**4a**, **7a**, **9b,** and **12b**) which showed good in vitro growth inhibitory activity against *S. aureus* (MIC = 15.63 µg/mL) were selected to be evaluated for their inhibitory activity against the resistant bacteria (MRSA); the results of this preliminary test revealed that Schiff base **12b** and the hydrazide derivative **7a** had inhibition zones of 15 mm and 18 mm, while that of gentamicin was 15 mm. Subsequently, the most active compounds were also screened for their *E. coli* DNA gyrase inhibitory activity, with comparison with novobiocin as a reference DNA gyrase inhibitor. It was found that compounds **3a,b, 4a, 9b,** and **12b** displayed the highest inhibitory capacities (IC_50_ = 2.26–5.78 µM), with that of novobiocin being IC_50_ = 4.17 µM. In addition, docking studies were performed to estimate the mode of binding of the mostly active compounds to *E. coli* DNA gyrase B active binding site compared with binding mode of novobiocin. From the analysis of the docking data, compounds (**3a,b, 4a, 9b,** and **12b**) adopted the best binding style with docking score = −6.83–−8.43 Kcal/mol, while novobiocin had a docking score = −6.30 Kcal/mol.

This study showed the importance of fused thienopyridine–quinolines which, with further optimization through structure-based design, could provide potent antimicrobial agents and DNA gyrase inhibitors through their new structural features able to deal with antimicrobial resistance.

## Figures and Tables

**Table 1:** The diameter of inhibition zones (mm) and minimum inhibitory concentration (MIC) values (µg/mL).

	M.O	*E. coli*	*S.* t*yphimurium*	*P. aeruginosa*	*B. subtilis*	*B. cereus*	*S. aureus*
Compound							
**3a**		18 (15.63)	15 (15.63)	15 (15.63)	10 (31.25)	13 (31.25)	12 (31.25)

**3b**		19 (15.63)	18 (15.63)	13 (31.25)	12 (31.25)	18 (15.63)	13 (31.25)

**4a**		18 (15.63)	15 (15.63)	13 (31.25)	15 (15.63)	16 (15.63)	18 (15.63)

**4b**		12 (31.25)	15 (15.63)	NA	10 (31.25)	NA	12 (31.25)

**5a**		12 (31.25)	NA	12 (31.25)	10 (31.25)	13 (31.25)	NA

**5b**		15 (15.63)	17 (15.63)	14 (15.63)	12 (31.25)	16 (15.63)	13 (31.25)

**6a**		13 (31.25)	NA	13 (31.25)	10 (31.25)	13 (31.25)	12 (31.25)

**6b**		16 (15.63)	12 (31.25)	12 (31.25)	15 (15.63)	15 (15.63)	NA

**7a**		16 (15.63)	11 (31.25)	11 (31.25)	17 (15.63)	15 (15.63)	19 (15.63)

**7b**		NA	10 (31.25)	12 (31.25)	8 (62.5)	11 (31.25)	10 (31.25)

**8a**		NA	NA	NA	11 (31.25)	9 (31.25)	11 (31.25)

**8b**		NA	NA	NA	9 (31.25)	9 (62.5)	8 (62.5)

**9a**		NA	NA	11 (31.25)	14 (15.63)	16 (15.63)	12 (31.25)

**9b**		20 (15.63)	19 (15.63)	18 (15.63)	15 (15.63)	18 (15.63)	16 (15.63)

**10**		NA	NA	NA	8 (62.5)	9 (62.5)	9 (31.25)

**11**		NA	NA	NA	9 (31.25)	8 (62.5)	8 (62.5)

**12a**		11 (31.25)	8 (62.5)	9 (62.5)	13 (31.25)	11 (31.25)	10 (31.25)

**12b**		18 (15.63)	16 (15.63)	16 (15.63)	15 (15.63)	15 (15.63)	16 (15.63)

**13**		8 (62.5)	7 (62.5)	11 (31.25)	15 (15.63)	16 (15.63)	12 (31.25)

**14**		15 (15.63)	12 (31.25)	10 (31.25)	16 (15.63)	17 (15.63)	13 (31.25)

**Amoxicillin**		20 (15.63)	21 (15.63)	18 (15.63)	17 (15.63)	19 (15.63)	19 (15.63)

M.O: microorganism; NA: no activity; Clear zone: mm; MIC ( ): µg/mL							

**Table 2:** The zones of inhibition (mm) against methicillin-resistant *Staphylococcus aureus* (MRSA).

M.O.	4a	7a	9b	12b	Gentamicin
MRSA	13	18	14	15	15

M.O: microorganism.

**Table 3:** *E. coli* DNA gyrase supercoiling inhibition (IC_50_ µM) of compounds **3a,b, 4a, 5b, 6a,b, 7a, 9b, 12b,** and **14** and reference antibiotic (novobiocin).

	3a	3b	4a	5b	6a	6b	7a	9b	12b	14	Novobiocin
**IC_50_ µM**	2.26 ± 0.19	4.50 ± 0.32	3.69 ± 0.15	16.70 ± 0.88	13.74 ± 0.65	5.95 ± 0.34	8.66 ± 0.61	5.78 ± 0.46	4.60 ± 0.28	11.72 ± 0.93	4.17 ± 0.32

**Table 4:** Docking results of the compounds (**3a,b, 4a, 5b, 6a,b, 7a, 9b, 12b,** and **14**) with *E. coli* DNA gyrase B kinase using MOE software version 2008.10.

Compd. NO.	Docking Score (Kcal/mol)	Amino Acid Residues (Bond Length A°)	Atoms of Compound	Type of Bond
**Novobiocin**	−6.30	Asn46 (1.88); Asp73 (1.89); Arg76	H(OH)(oxan-4-yl); H(OCONH_2_); C_6_H_2_(coumarin)	H–don H–don Arene–cation
**3a**	−7.32	Arg136 (2.64); Thr165 (3.00)	O(CONH_2_); N(pyridine)	H–acc H–acc
**3b**	−6.83	Arg136 (2.52); Thr165 (3.10)	O(CONH_2_); N(pyridine)	H–acc H–acc
**4a**	−7.46	Arg136 (2.67); Thr165 (3.01)	O(CONH_2_); N(pyridine)	H–acc H–acc
**5b**	−6.22	Asn46 (3.29)	N(pyridine)	H–acc
**6a**	−6.24	Asn46 (3.00); Val120 (3.2)	N(pyridine); O(NHCOCH_2_)	H–acc H–acc
**6b**	−6.75	Asn46 (2.25); Val120 (2.72); Arg76	N(pyridine); O(NHCOCH_2_) thiophene	H–acc H–acc Arene–cation
**7a**	−6.25	Asn46 (3.28); Val120 (2.95)	N(pyridine); O(NHCOCH_2_)	H–acc H–acc
**9b**	−8.43	Asn46 (2.46); Asn46 (2.79)	O(NHCOCH_2_); N(pyridine)	H–acc H–acc
**12b**	−7.43	Asn46 (2.98); Arg76 (2.80)	N(N=CH); O(OCH_3_)	H–acc H–acc
**14**	−5.45	Asn46 (2.23); Arg76	N(pyridine); thiophene	H–acc Arene–cation

## Supplementary Materials

The following are available online, Figures S1–S53: NMR and MS spectra of compounds **2a**–**14**.
