# Supplementary material for: Synthesis, Docking Studies, and In Vitro Evaluation of Some Novel Thienopyridines and Fused Thienopyridine–Quinolines as Antibacterial Agents and DNA Gyrase Inhibitors
Source: Molecules. 2019 Oct 10;24(20):3650. doi: 10.3390/molecules24203650 (PMC6832920; doi:10.3390/molecules24203650)
Supplement: Supplementary file 1 [file molecules-24-03650-s001.pdf]

Supplementary Materials

Synthesis, Docking Studies and *In Vitro* Evaluation of Some Novel Thienopyridines and Fused Thienopyridine-Quinolines as Antibacterial Agents and DNA Gyrase Inhibitors

Eman M. Mohi El-Deen [1]*, Eman A. Abd El-Meguid [2], Sherifa Hasabelnaby[3], Eman A. Karam[4], and Eman S. Nossier[5],

[1]*Department of Therapeutic Chemistry, National Research Centre, Dokki, Cairo, 12622 Egypt;* *e.mohi.2010@live.com*
[2]*Department of Chemistry of Natural and Microbial Products, National Research Centre, Dokki, Cairo, 12622 Egypt.*
[3]*Pharmaceutical chemistry department, Faculty of Pharmacy, Helwan University, Ein Helwan, Cairo, 11795 Egypt.*
[4]*Microbiology chemistry department, National Research Centre, Dokki, Cairo, 12622 Egypt.*
[5]*Department of Pharmaceutical Medicinal Chemistry, Faculty of Pharmacy (Girls), Al-Azhar University, Cairo 11754, Egypt.*

Table of contents

	Page
Figure S1. ^{1}H-NMR (400 MHz, DMSO-d_6) spectrum of **2a**.	S3
Figure S2. Mass spectrum of **2a**.	S3
Figure S3. ^{1}H-NMR (400 MHz, DMSO-d_6) spectrum of **2b**.	S4
Figure S4. Mass spectrum of **2b**.	S4
Figure S5. ^{1}H-NMR (400 MHz, DMSO- d_6) spectrum of **3a**.	S5
Figure S6. ^{13}C-NMR (100 MHz, DMSO- d_6) spectrum of **3a**.	S6
Figure S7. Mass spectrum of **3a**.	S6
Figure S8. ^{1}H-NMR (400 MHz, DMSO- d_6) spectrum of **3b**.	S7
Figure S9. ^{13}C-NMR (100 MHz, DMSO- d_6) spectrum of **3b**.	S8
Figure S10. Mass spectrum of **3b**.	S8
Figure S11. ^{1}H-NMR (400 MHz, DMSO- d_6) spectrum of **4a**.	S9
Figure S12. ^{13}C-NMR (100 MHz, DMSO- d_6) spectrum of **4a**.	S10
Figure S13. Mass spectrum of **4a**.	S10
Figure S14. ^{1}H-NMR (400 MHz, DMSO- d_6) spectrum of **4b**.	S11
Figure S15. Mass spectrum of **4b**.	S12
Figure S16. ^{1}H-NMR (400 MHz, $CDCl_3$) spectrum of **5a**.	S13
Figure S17. ^{13}C-NMR (100 MHz, DMSO- d_6) spectrum of **5a**.	S14
Figure S18. Mass spectrum of **5a**.	S14
Figure S19. ^{1}H-NMR (400 MHz, DMSO- d_6) spectrum of **5b**.	S15
Figure S20. ^{13}C-NMR (100 MHz, DMSO- d_6) spectrum of **5b**.	S15
Figure S21. Mass spectrum of **5b**.	S16

Figure S22. ^{1}H-NMR (400 MHz, DMSO- d_6) spectrum of **6a**. S16
Figure S23. Mass spectrum of **6a**. S17
Figure S24. ^{1}H-NMR (400 MHz, DMSO- d_6) spectrum of **6b**. S17
Figure S25. ^{13}C-NMR (100 MHz, DMSO- d_6) spectrum of **6b**. S18
Figure S26. Mass spectrum of **6b**. S18
Figure S27. ^{1}H-NMR (400 MHz, DMSO- d_6) spectrum of **7a**. S19
Figure S28. Mass spectrum of **7a**. S19
Figure S29. ^{1}H-NMR (400 MHz, DMSO- d_6) spectrum of **7b**. S20
Figure S30. Mass spectrum of **7b**. S20
Figure S31. ^{1}H-NMR (400 MHz, DMSO- d_6) spectrum of **8a**. S21
Figure S32. ^{13}C-NMR (100 MHz, DMSO- d_6) spectrum of **8a**. S22
Figure S33. Mass spectrum of **8a**. S22
Figure S34. ^{1}H-NMR (400 MHz, DMSO- d_6) spectrum of **8b**. S23
Figure S35. Mass spectrum of **8b**. S23
Figure S36. ^{1}H-NMR (400 MHz, $CDCl_3$) spectrum of **9a**. S24
Figure S37. ^{13}C-NMR (100 MHz, $CDCl_3$) spectrum of **9a**. S24
Figure S38. Mass spectrum of **9a**. S25
Figure S39. ^{1}H-NMR (400 MHz, DMSO- d_6) spectrum of **9b**. S25
Figure S40. Mass spectrum of **9b**. S26
Figure S41. ^{1}H-NMR (400 MHz, DMSO- d_6) spectrum of **10**. S26
Figure S42. Mass spectrum of **10**. S27
Figure S43. ^{1}H-NMR (400 MHz, DMSO- d_6) spectrum of **11**. S27
Figure S44. Mass spectrum of **11**. S28
Figure S45. ^{1}H-NMR (400 MHz, DMSO- d_6) spectrum of **12a**. S28
Figure S46. ^{13}C-NMR (100 MHz, DMSO- d_6) spectrum of **12a**. S29
Figure S47. Mass spectrum of **12a**. S29
Figure S48. ^{1}H-NMR (400 MHz, DMSO- d_6) spectrum of **12b**. S30
Figure S49. Mass spectrum of **12b** S30
Figure S50. ^{1}H-NMR (400 MHz, $CDCl_3$) spectrum of **13**. S31
Figure S51. Mass spectrum of **13**. S32
Figure S52. ^{1}H-NMR (400 MHz, DMSO- d_6) spectrum of **14** S32
Figure S53. Mass spectrum of **14**. S33

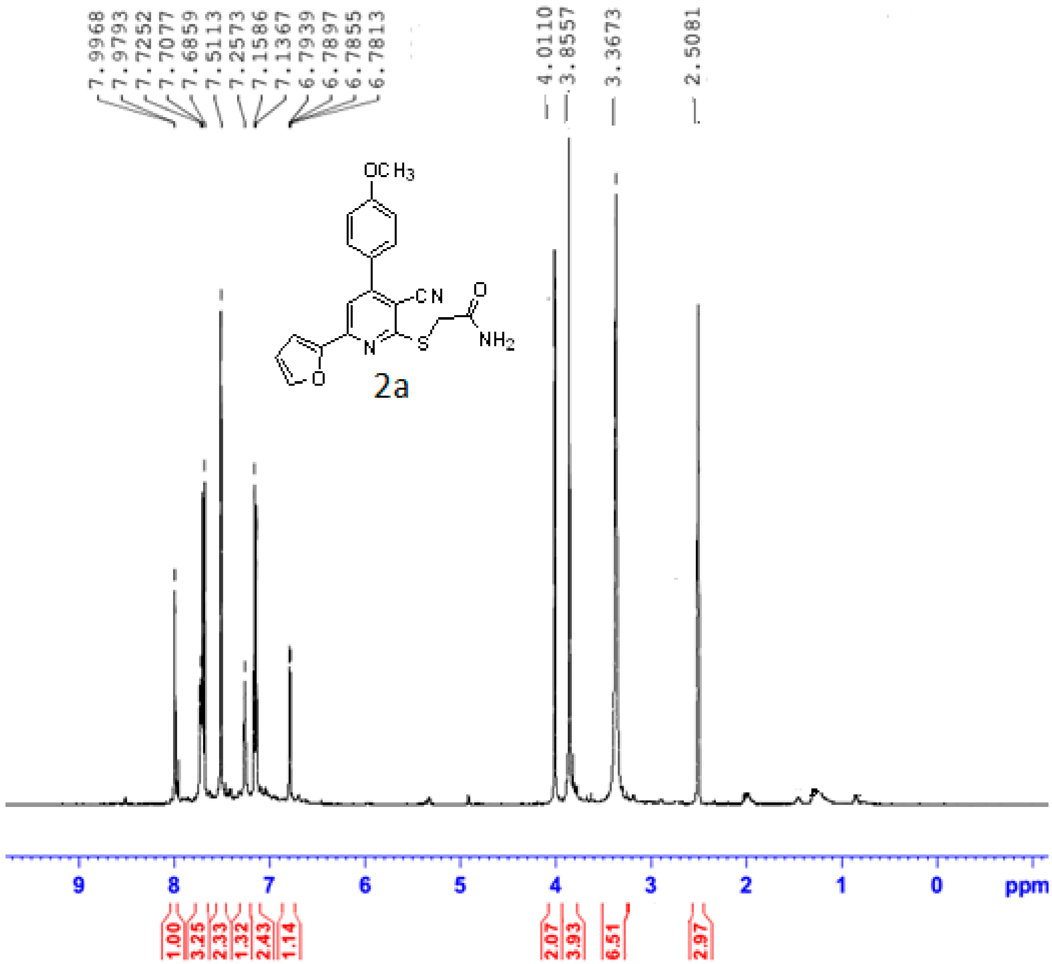

Fig. S1 ^{1}H NMR (400 MHz) in DMSO-d_6 of compound **2a**

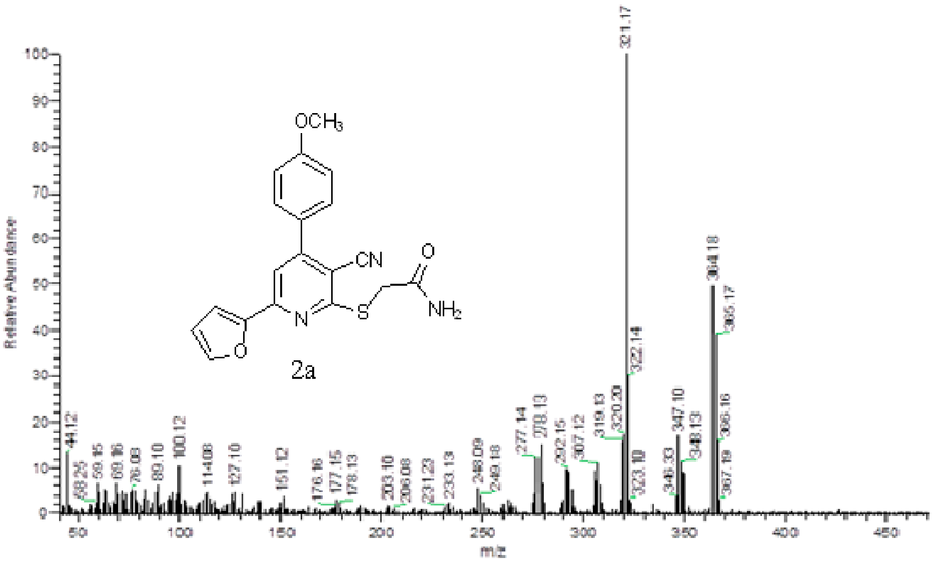

Fig. S2 Mass spectrum of compound **2a**

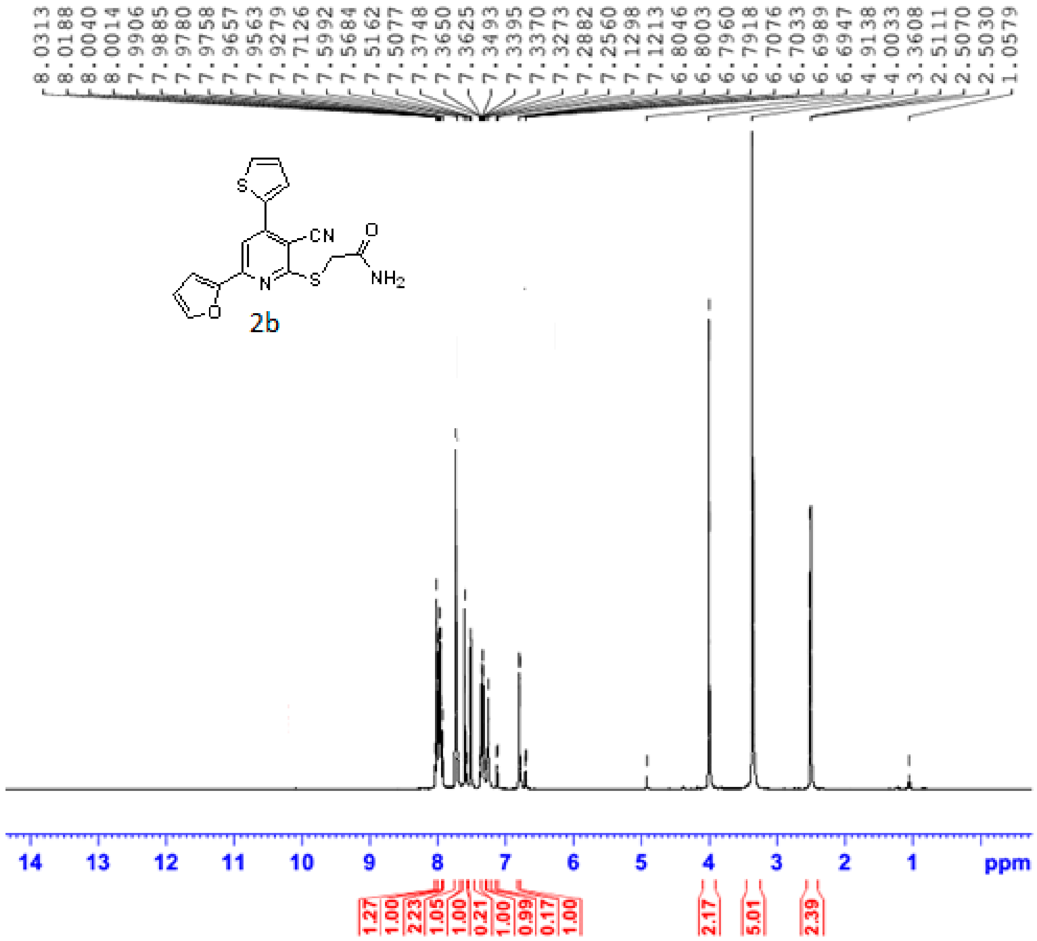

Fig. S3 ^{1}H NMR (400 MHz) in DMSO-d_6 of compound **2b**

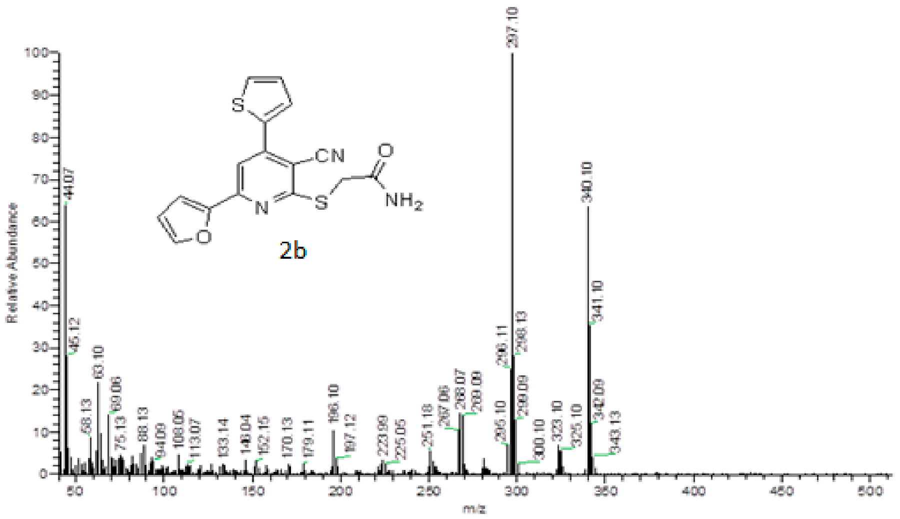

Fig. S4 Mass spectrum of compound **2b**

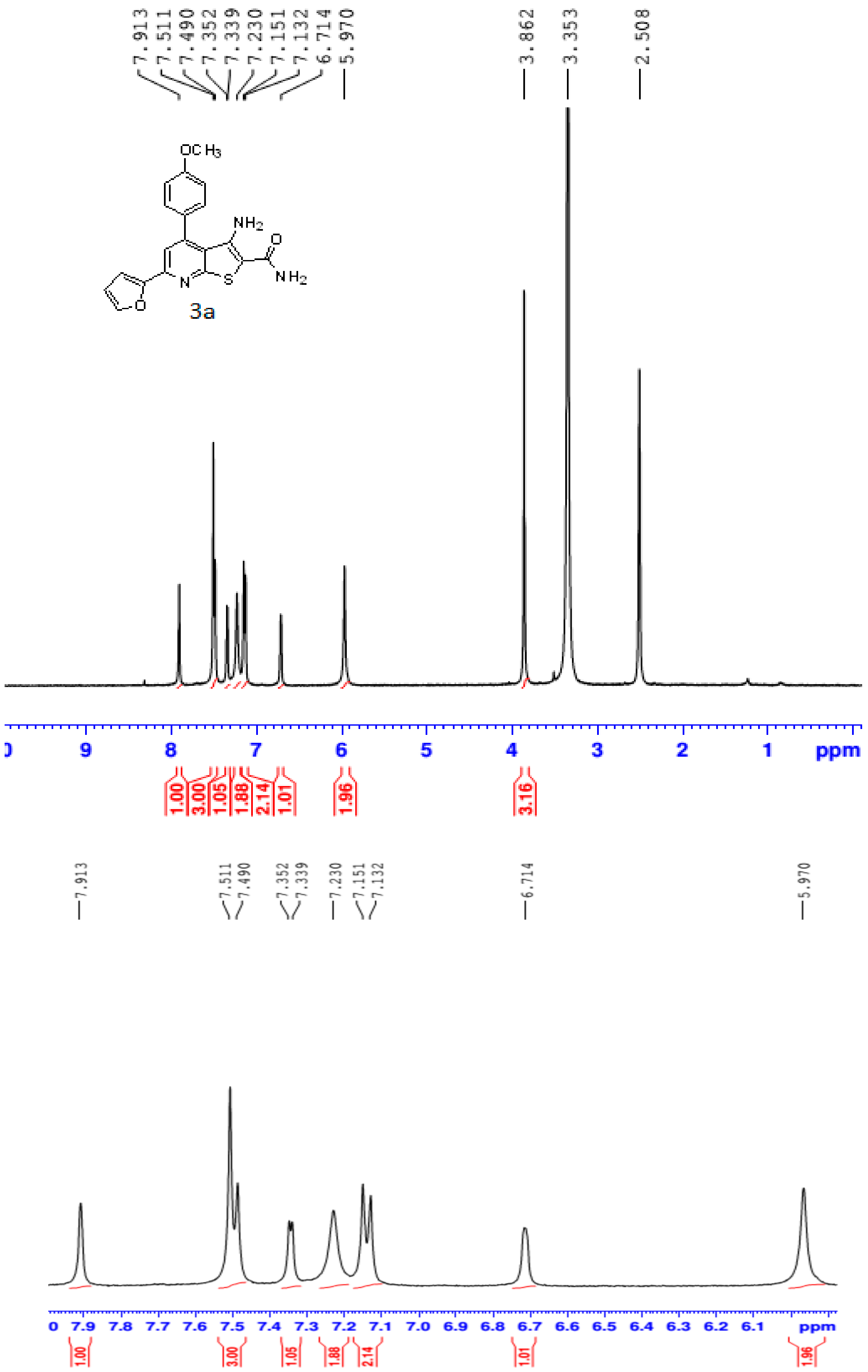

Fig. S5 ^{1}H NMR (400 MHz) in DMSO-d_6 of compound **3a**

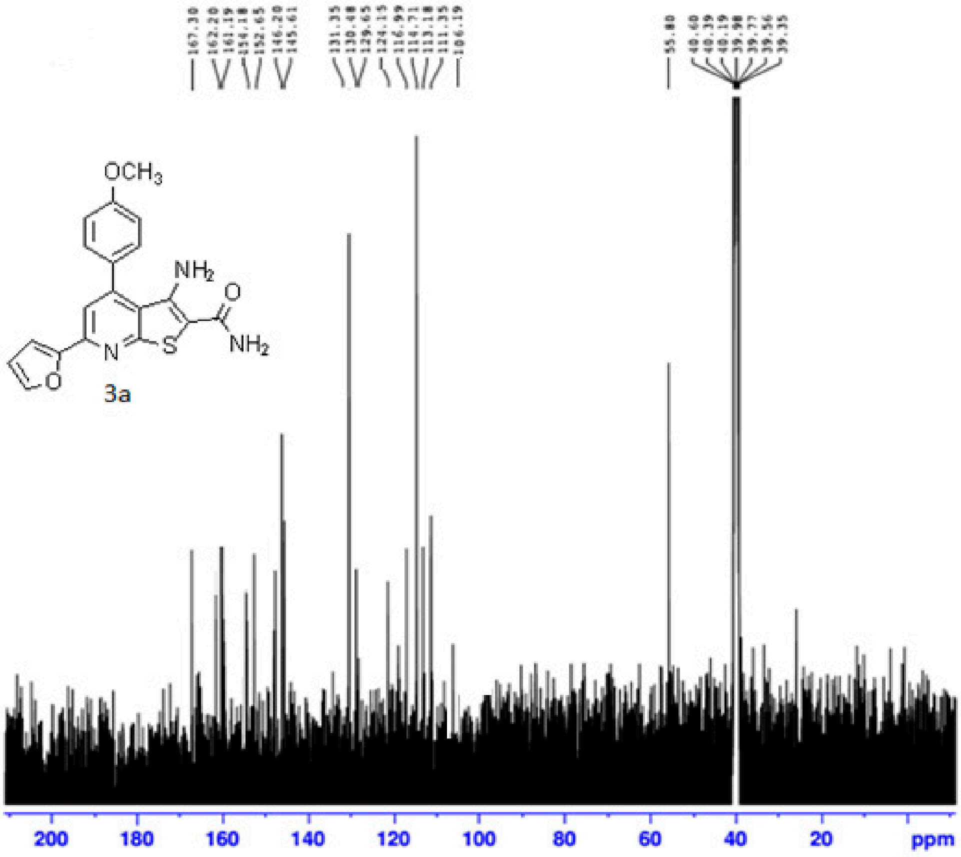

Fig. S6 ^{13}C NMR (100 MHz) in DMSO-d_6 of compound **3a**

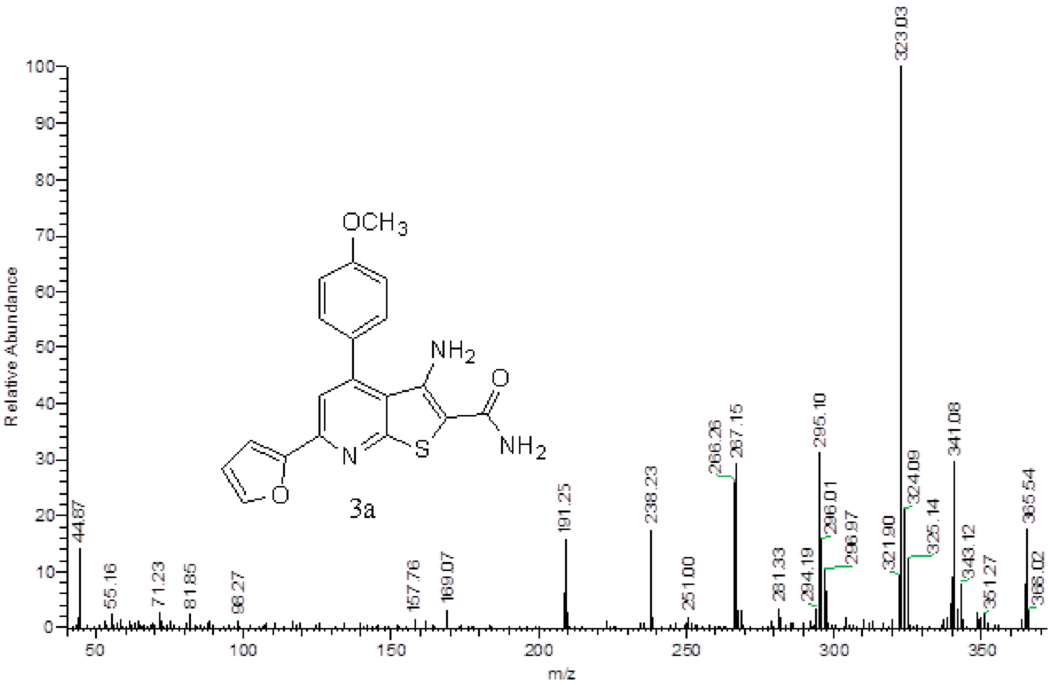

Fig. S7 Mass spectrum of compound **3a**

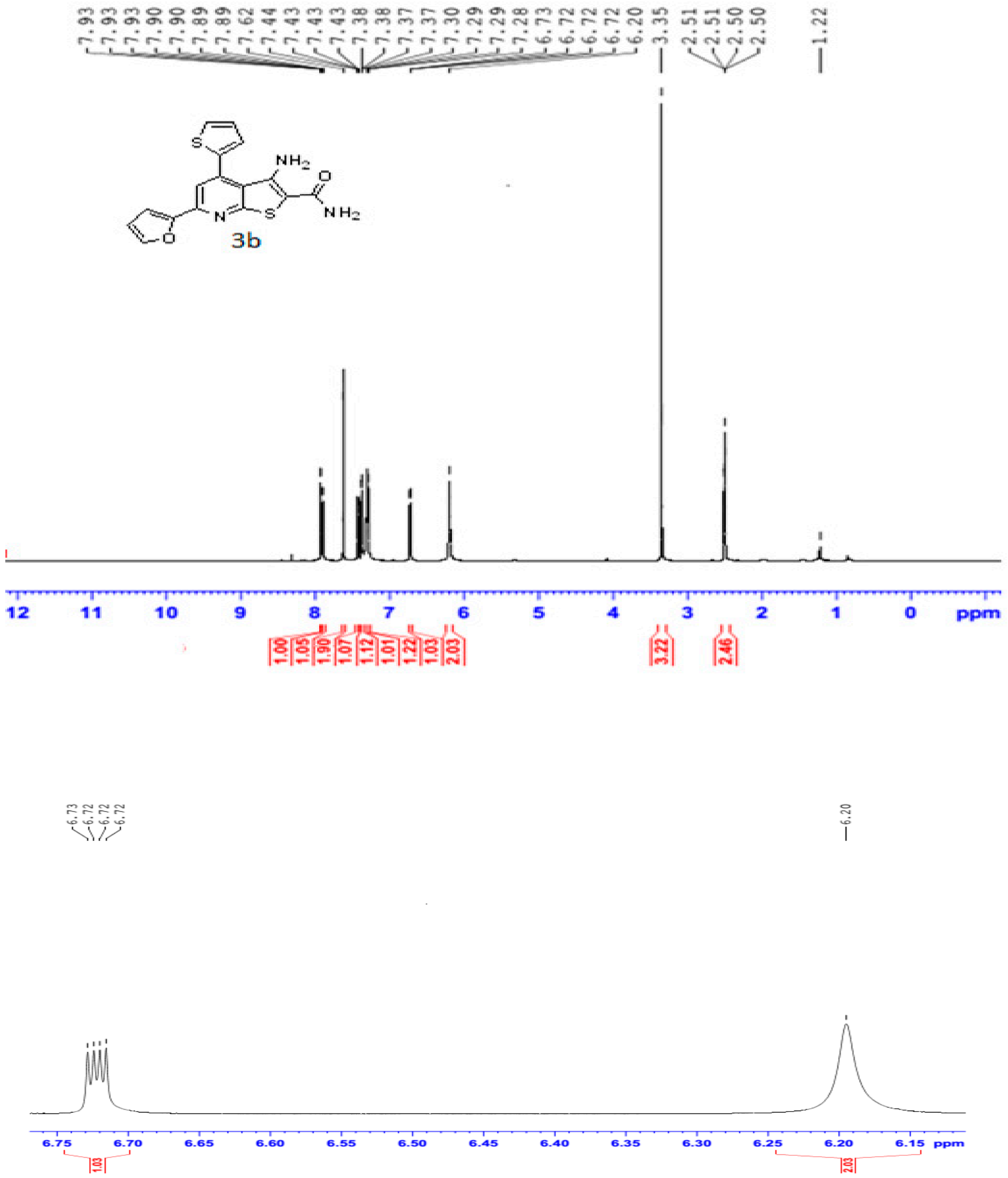

Fig. S8 ^{1}H NMR (400 MHz) in DMSO-d_6 of compound **3b**

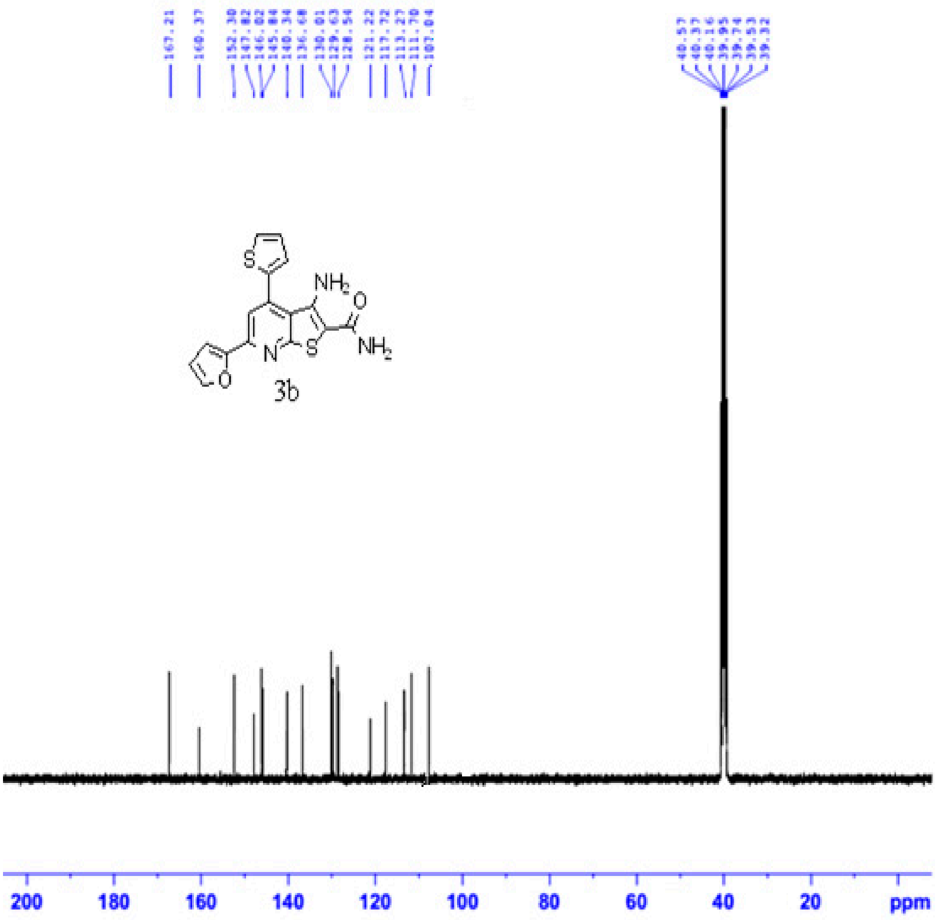

Fig. S9 ^{13}C NMR (100 MHz) in DMSO-d_6 of compound **3b**

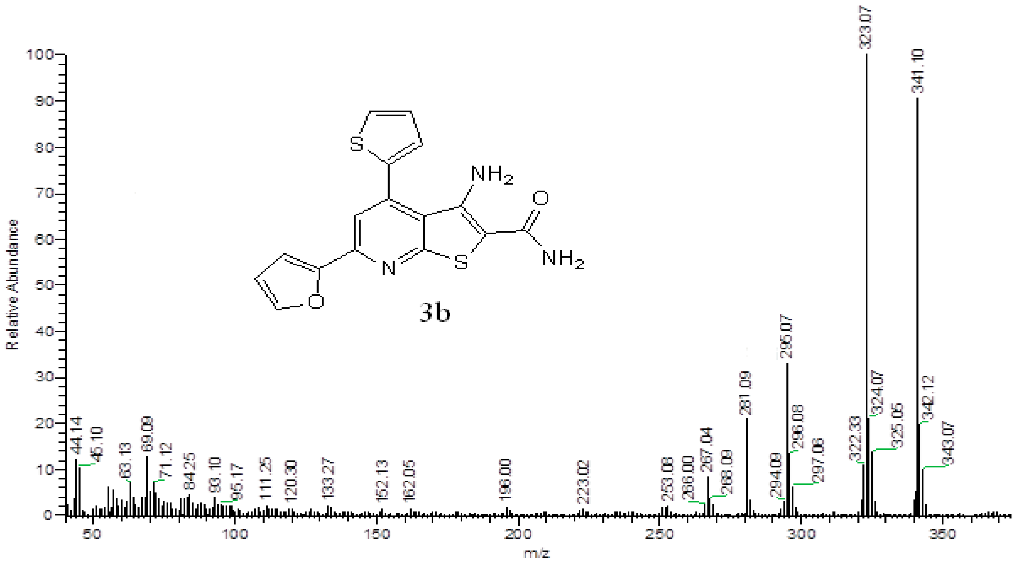

Fig. S10 Mass spectrum of compound **3b**

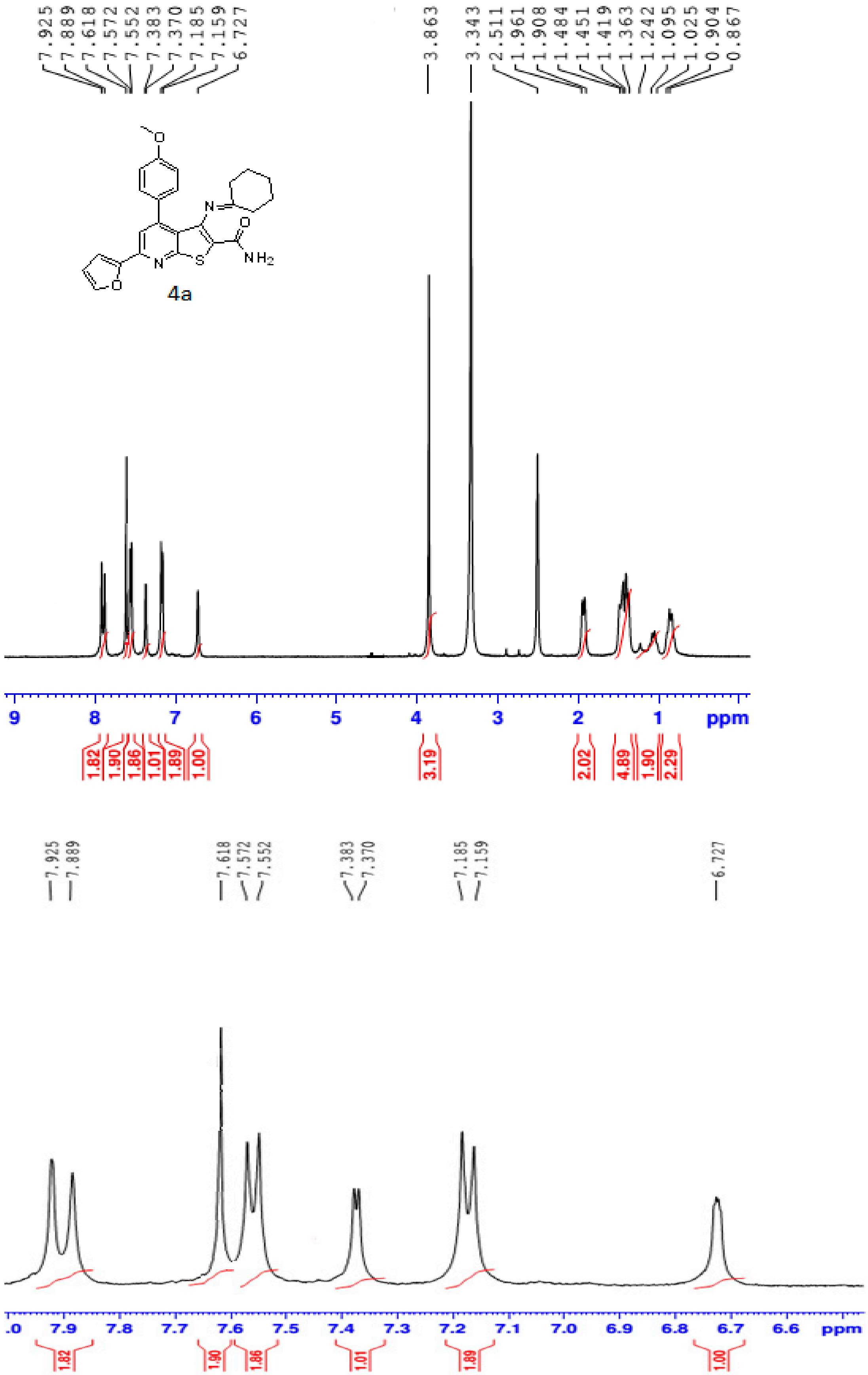

Fig. S11 ^{1}H NMR (400 MHz) in DMSO-d_6 of compound **4a**

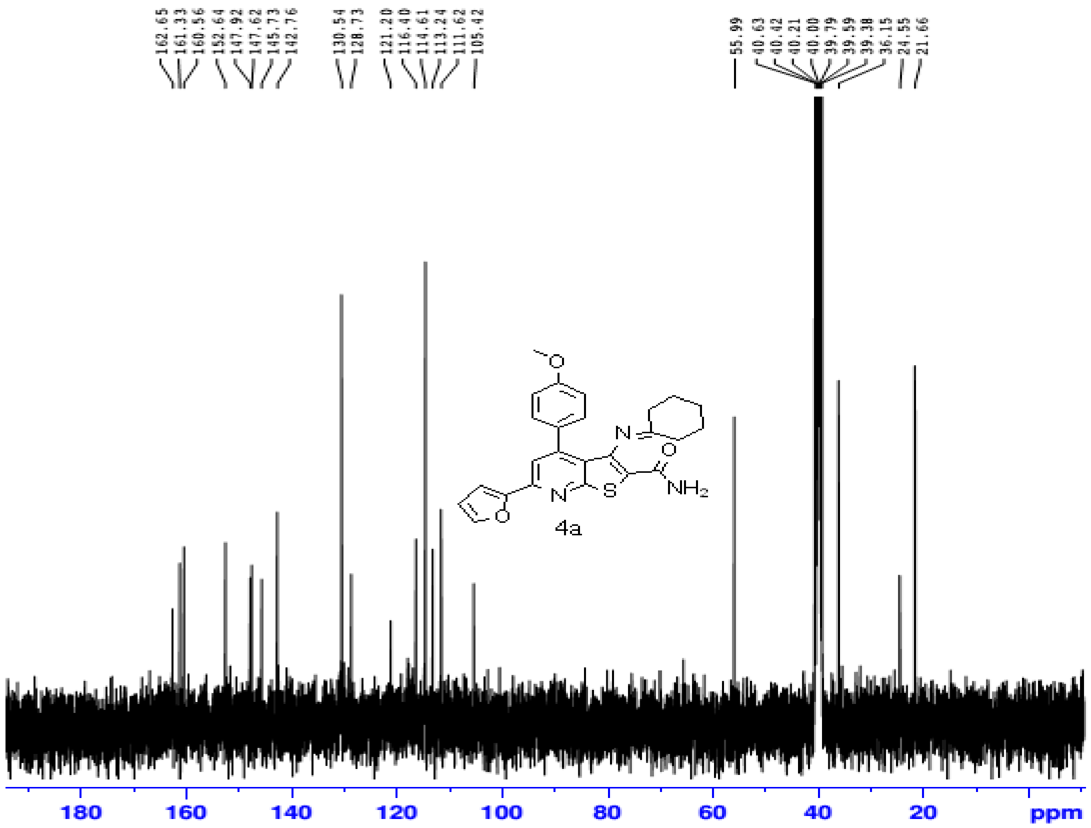

Fig. S12 ^{13}C NMR (100 MHz) in DMSO-d_6 of compound **4a**

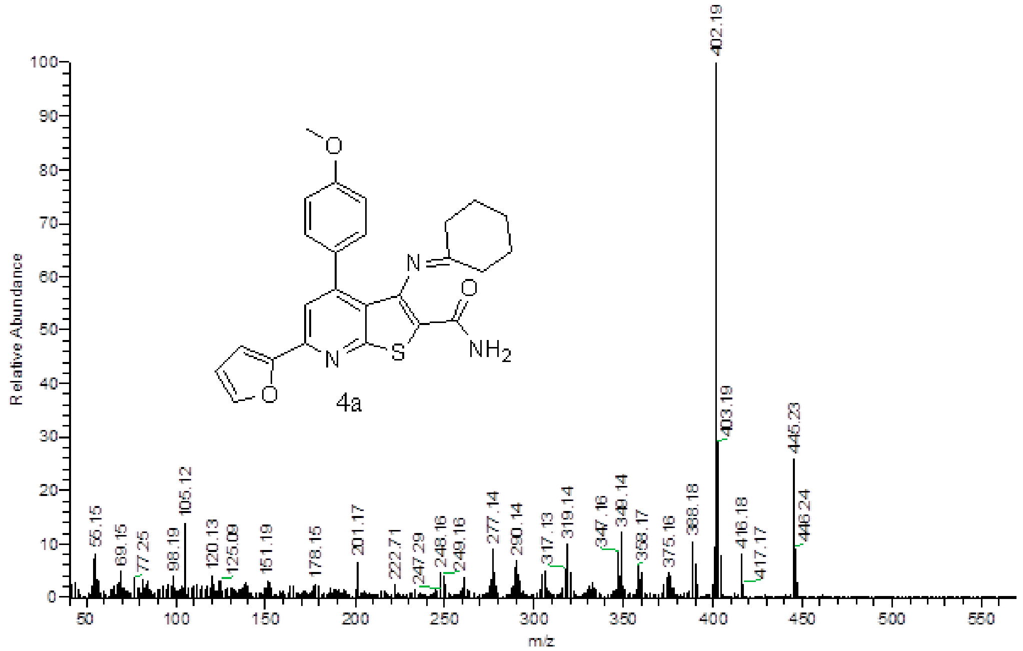

Fig. S13 Mass spectrum of compound **4a**

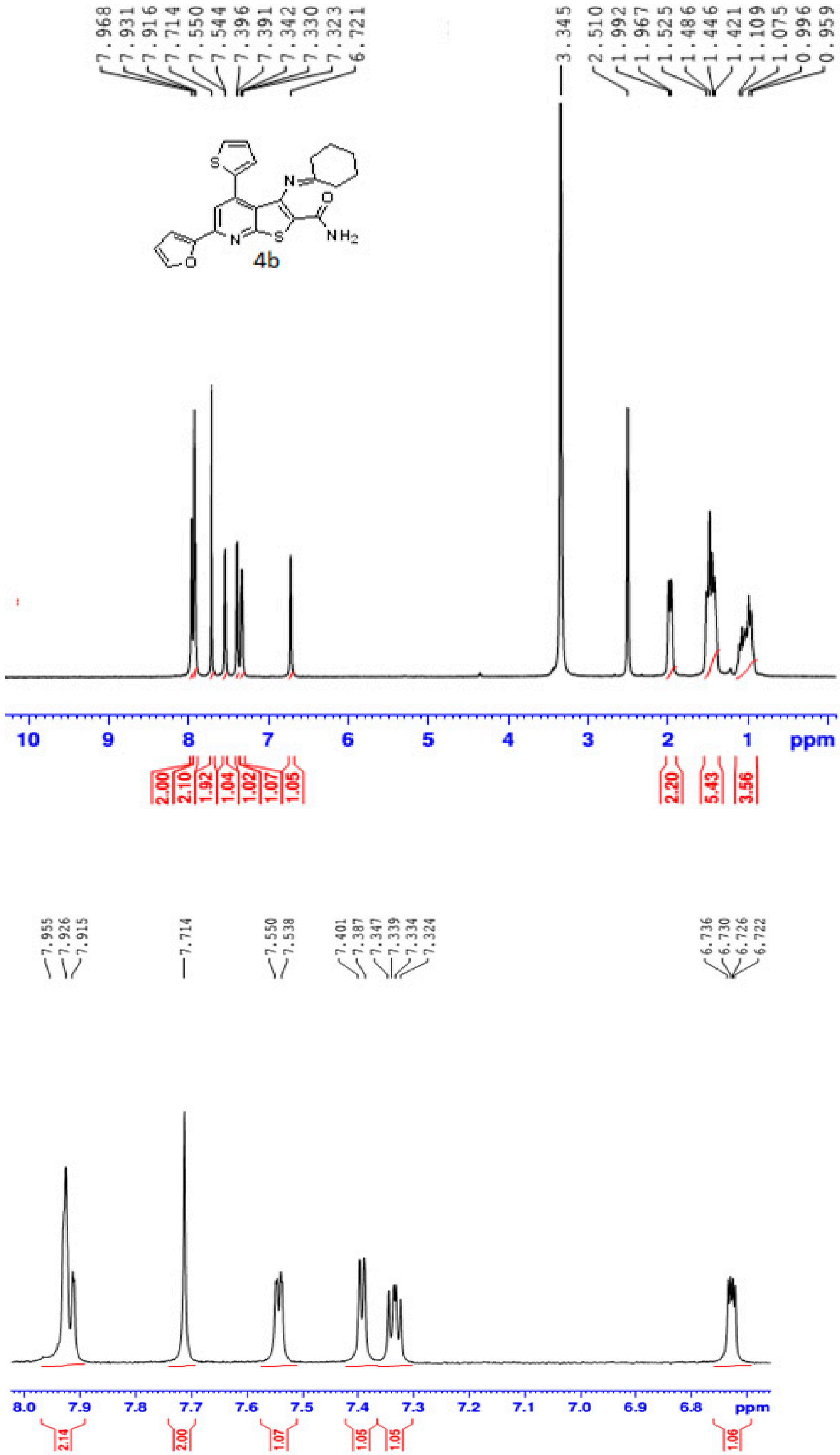

Fig. S14 ^{1}H NMR (400 MHz) in DMSO-d_6 of compound **4b**

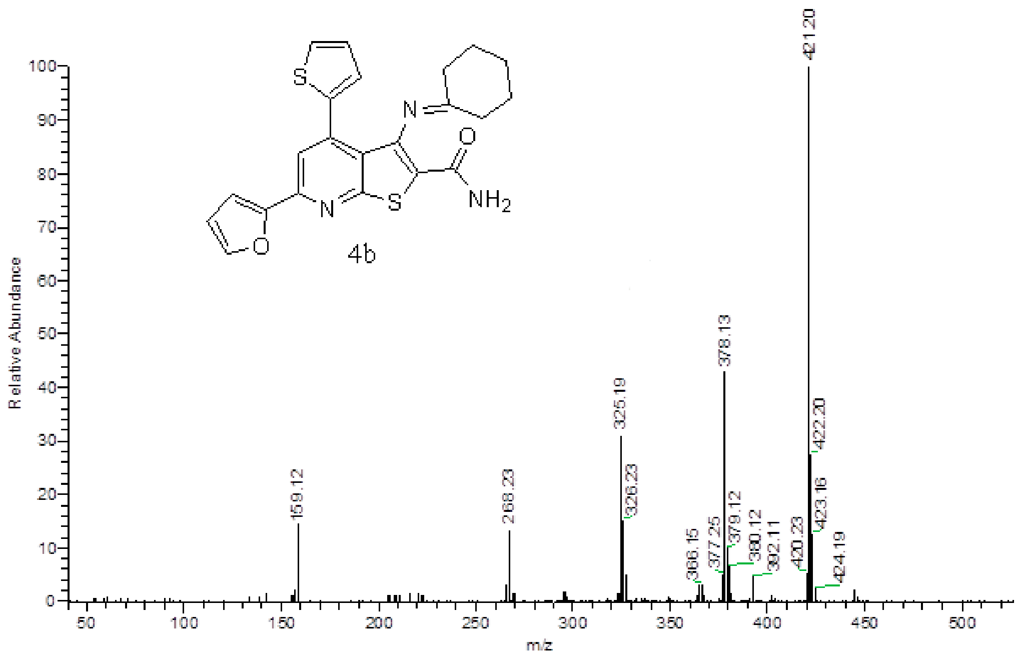

Fig. S15 Mass spectrum of compound **4b**

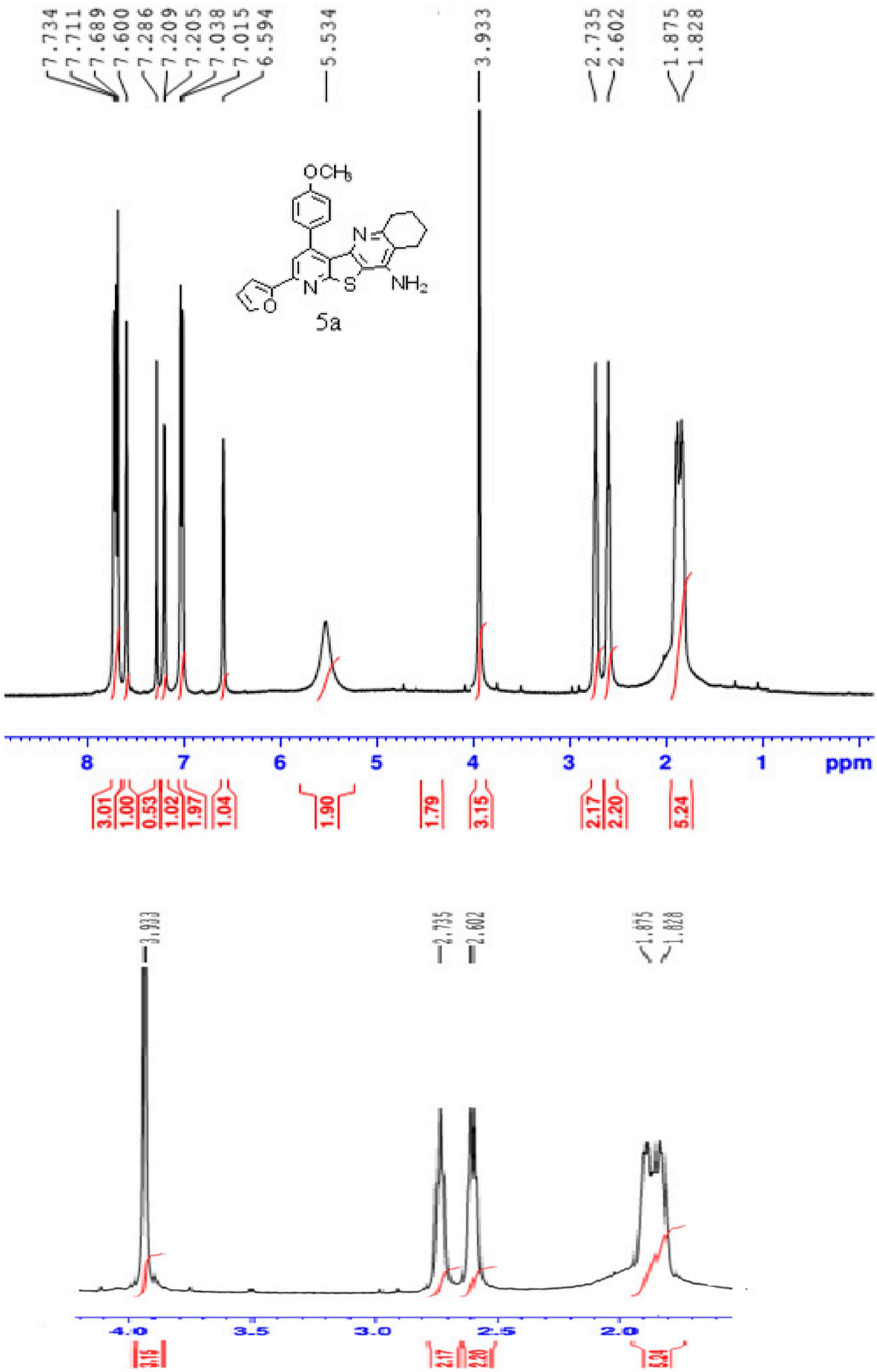

Fig. S16 ^{1}H NMR (400 MHz) spectrum in $CDCl_3$ of compound **5a**

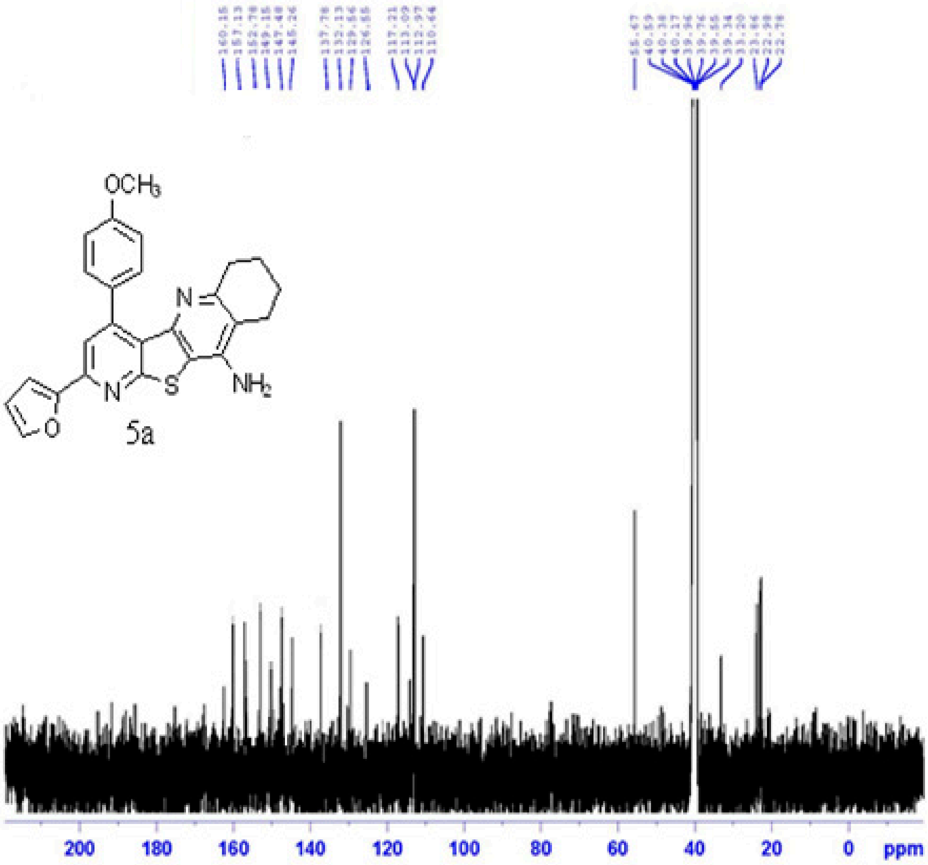

Fig. S17 ^{13}C NMR (100 MHz) in DMSO-d_6 of compound **5a**

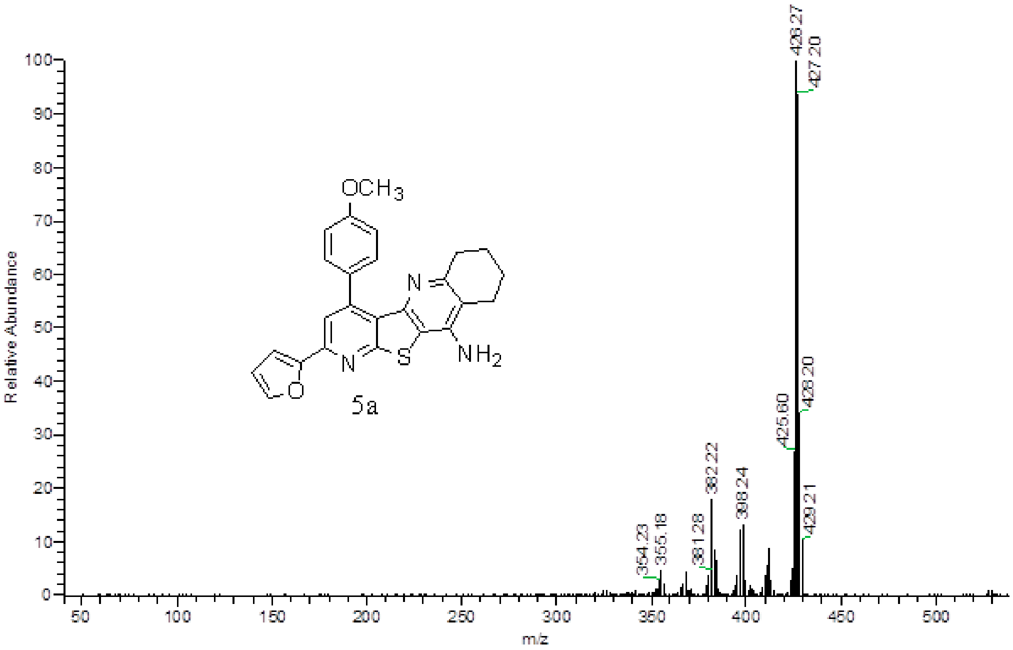

Fig. S18 Mass spectrum of compound **5a**

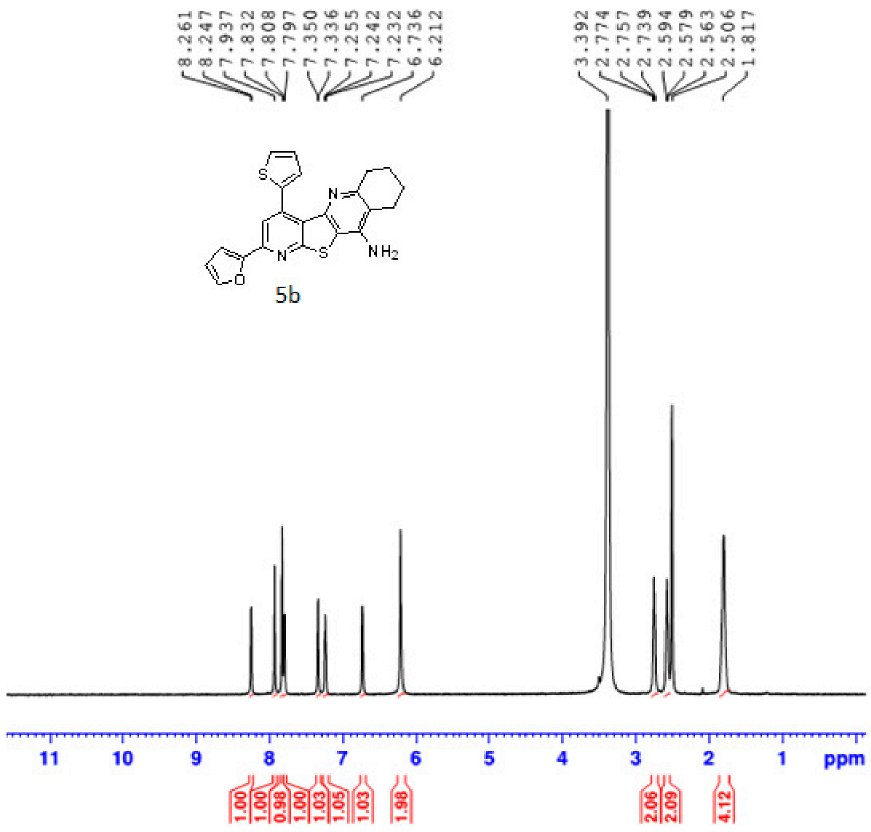

Fig. S19 ^{1}H NMR (400 MHz) in DMSO-d_6 of compound **5b**

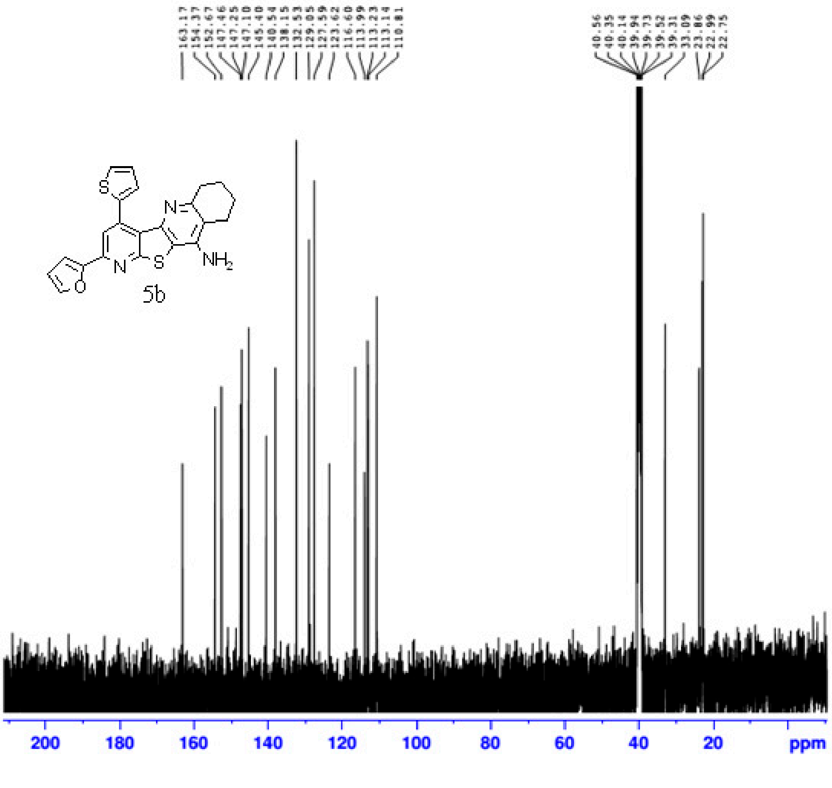

Fig. S20 ^{13}C NMR (100 MHz) in DMSO-d_6 of compound **5b**

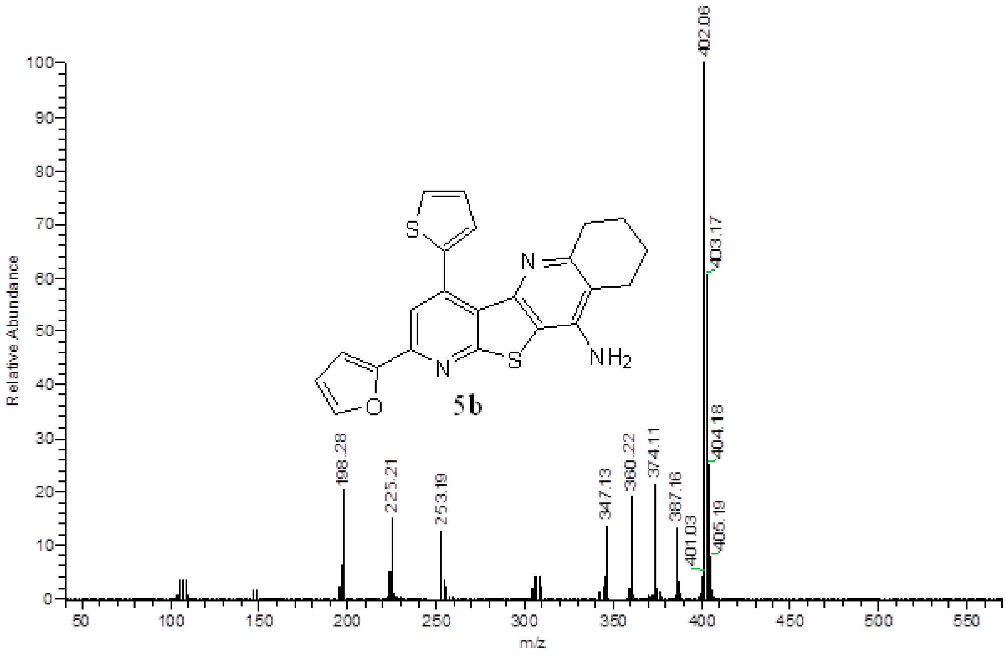

Fig. S21 Mass spectrum of compound **5b**

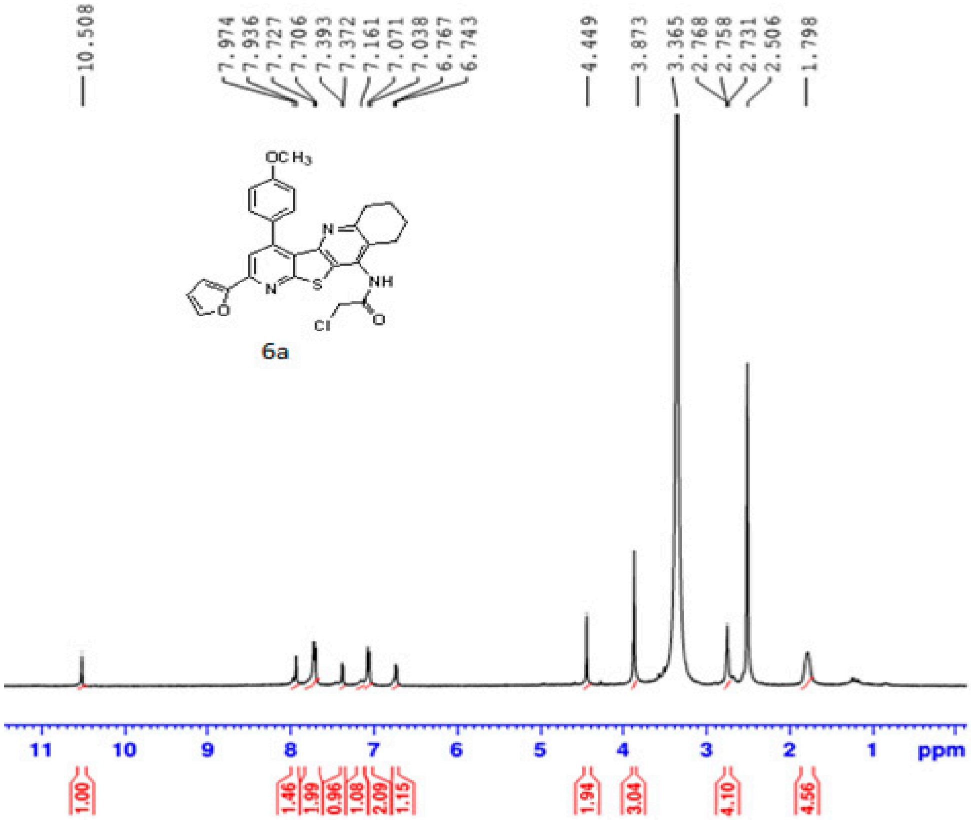

Fig. S22 ^{1}H NMR (400 MHz) in DMSO-d_6 of compound **6a**

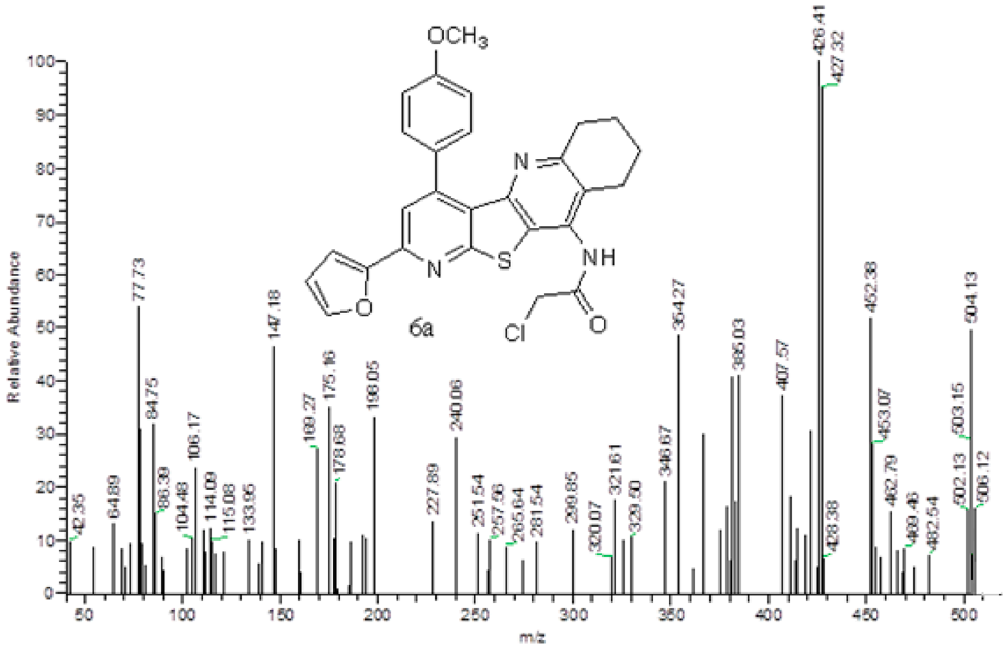

Fig. S23 Mass spectrum of compound **6a**

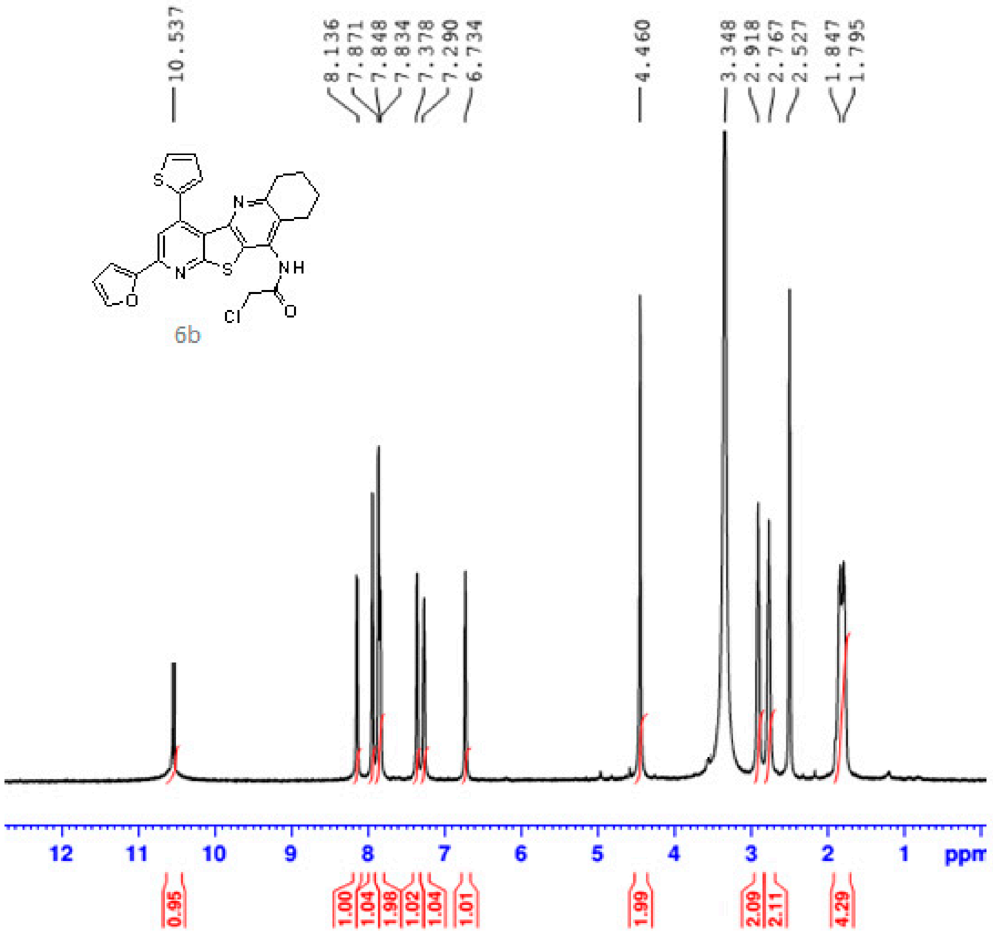

Fig. S24 ^{1}H NMR (400 MHz) in DMSO-d_6 of compound **6b**

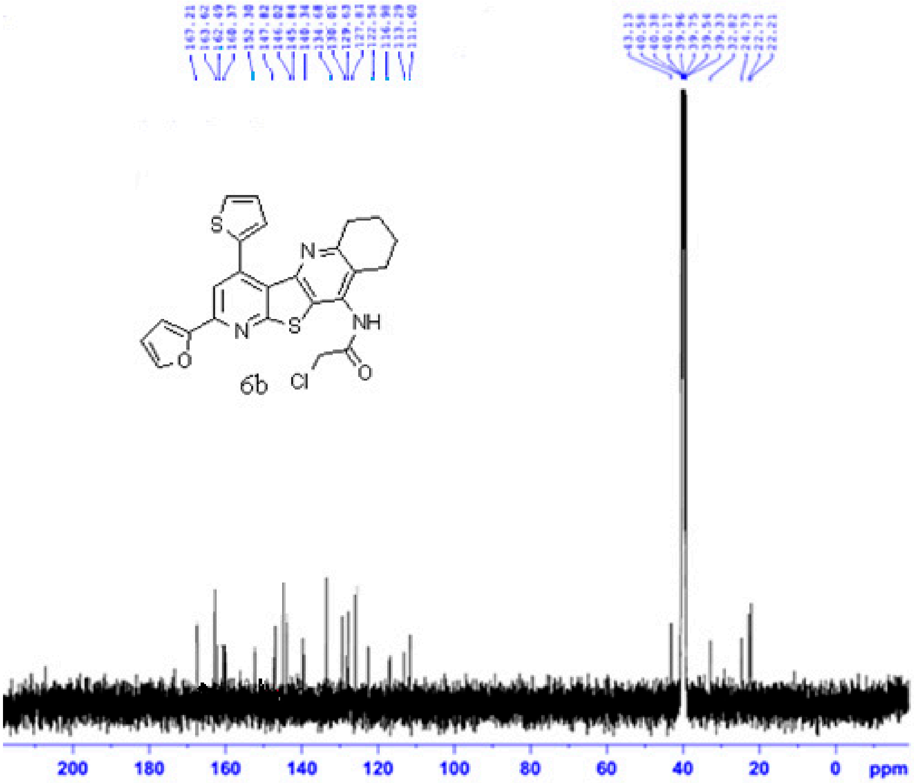

Fig. S25 ^{13}C NMR (100 MHz) in DMSO-d_6 of compound **6b**

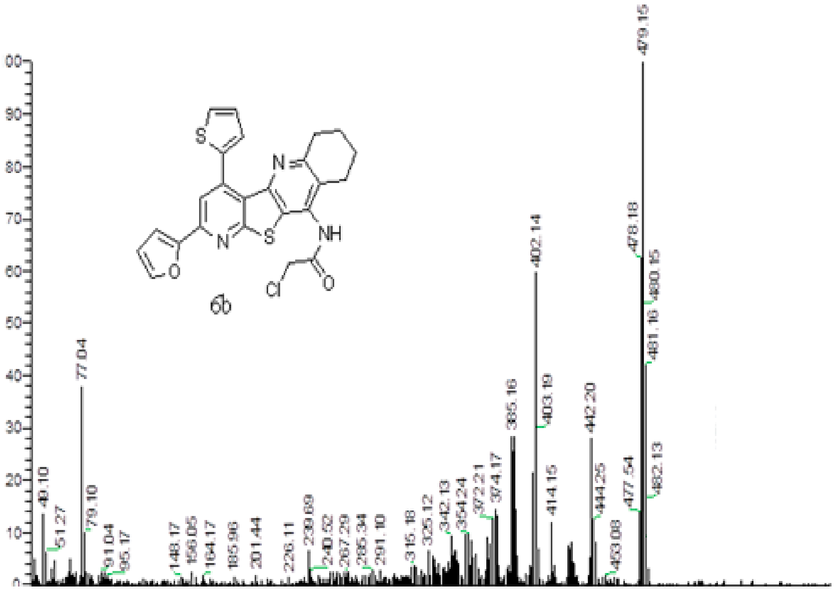

Fig. S26 Mass spectrum of compound **6b**

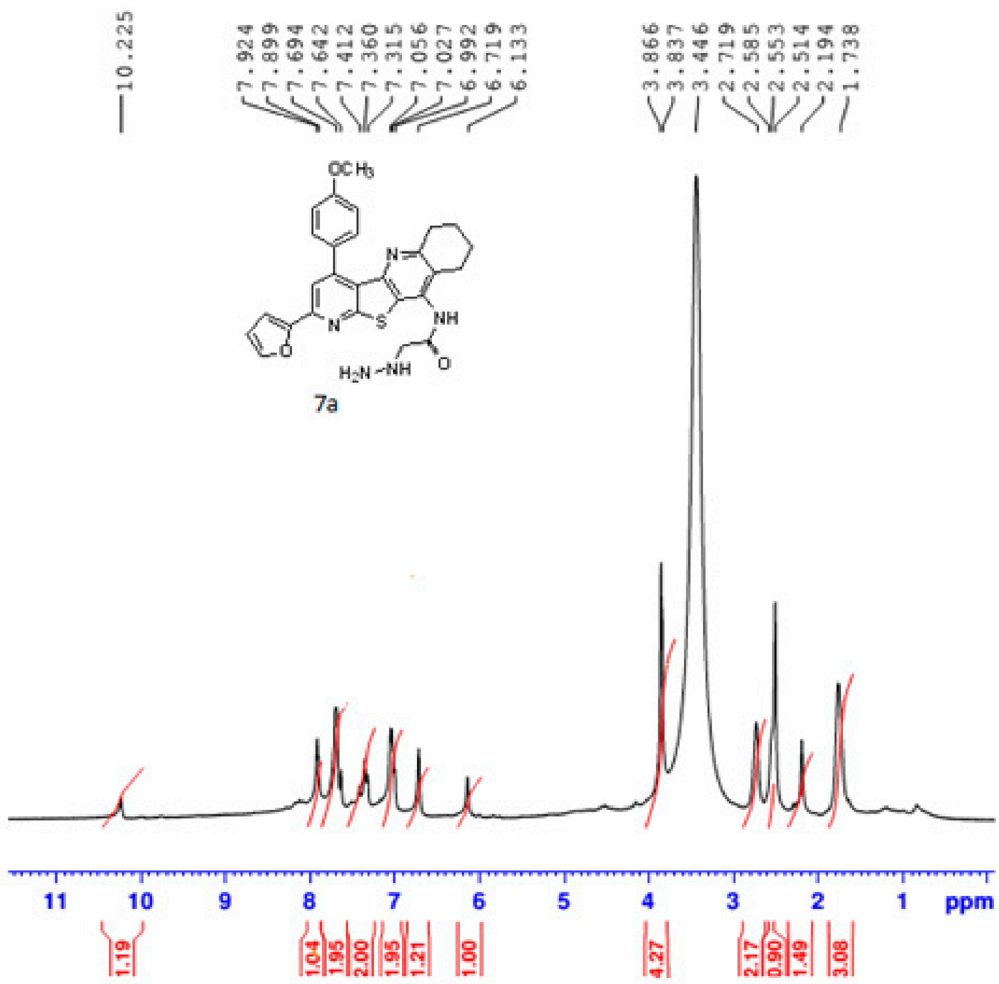

Fig. S27 ^{1}H NMR (400 MHz) in DMSO-d_6 of compound **7a**

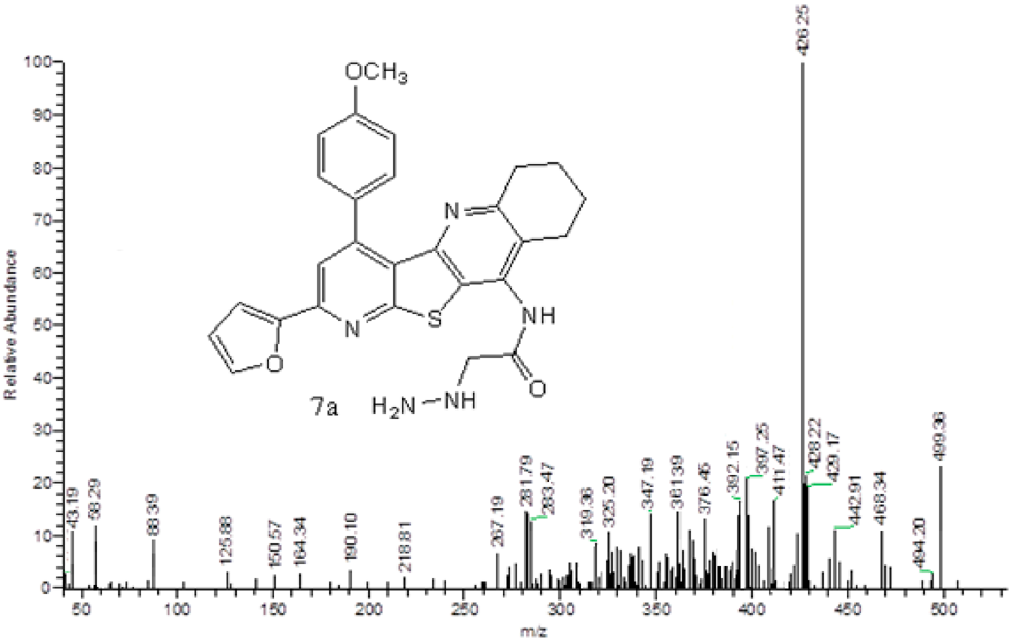

Fig. S28 Mass spectrum of compound **7a**

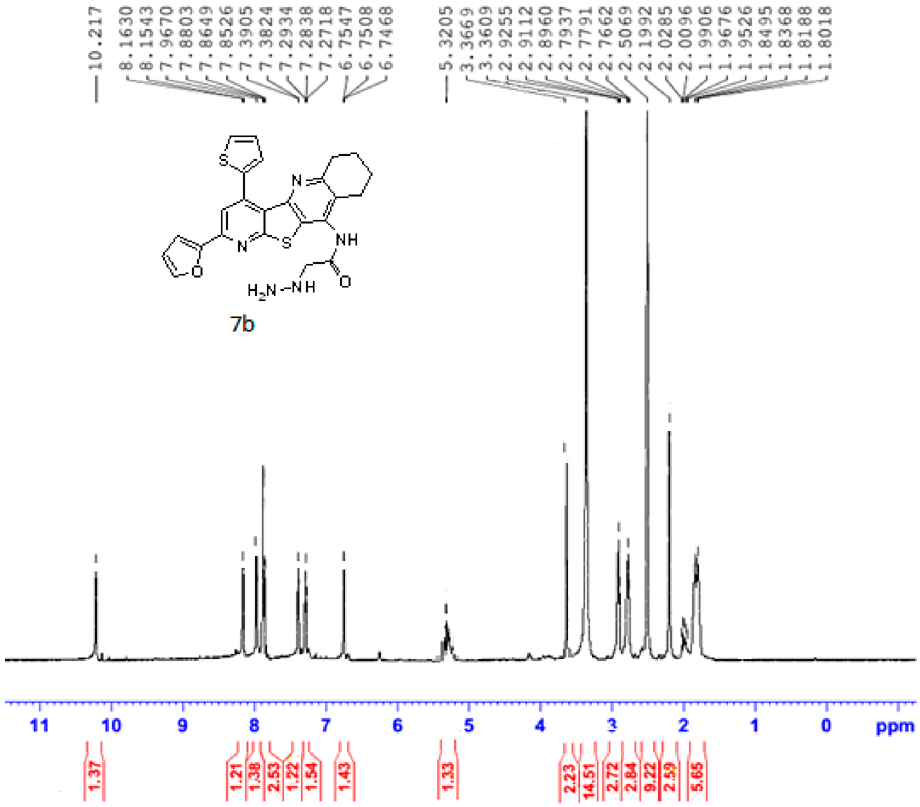

Fig. S29 ^{1}H NMR (400 MHz) in DMSO-d_6 of compound **7b**

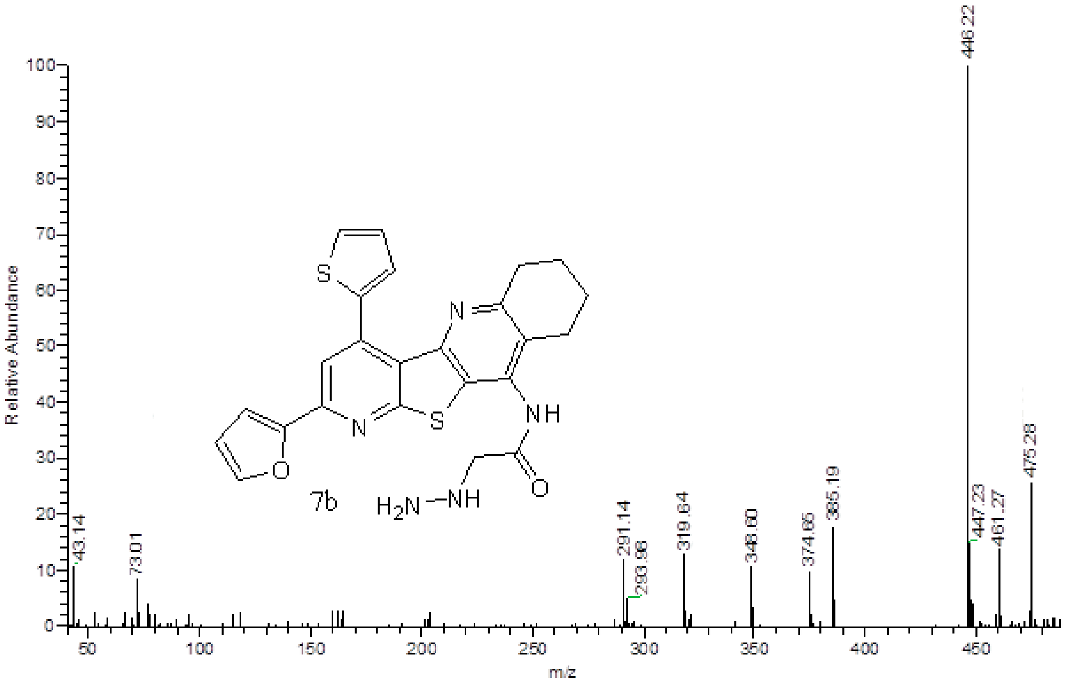

Fig. S30 Mass spectrum of compound **7b**

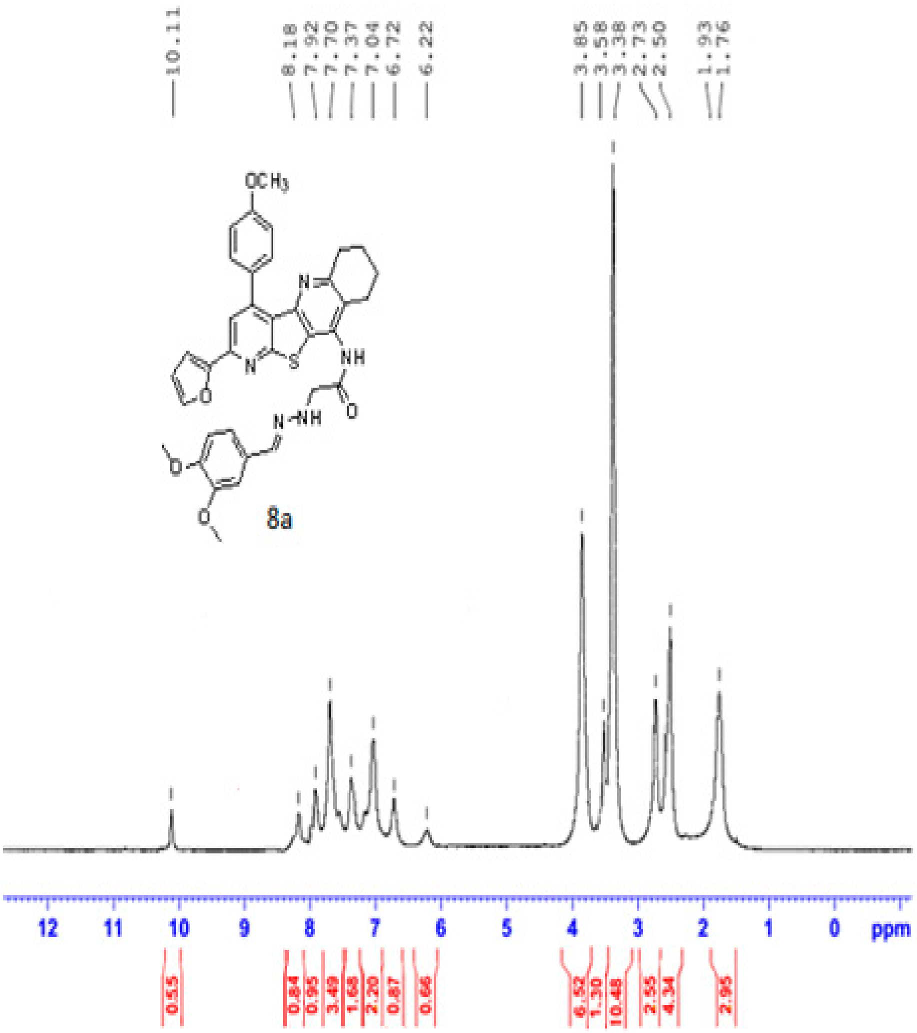

Fig. S31 ^{1}H NMR (400 MHz) in DMSO-d_6 of compound **8a**

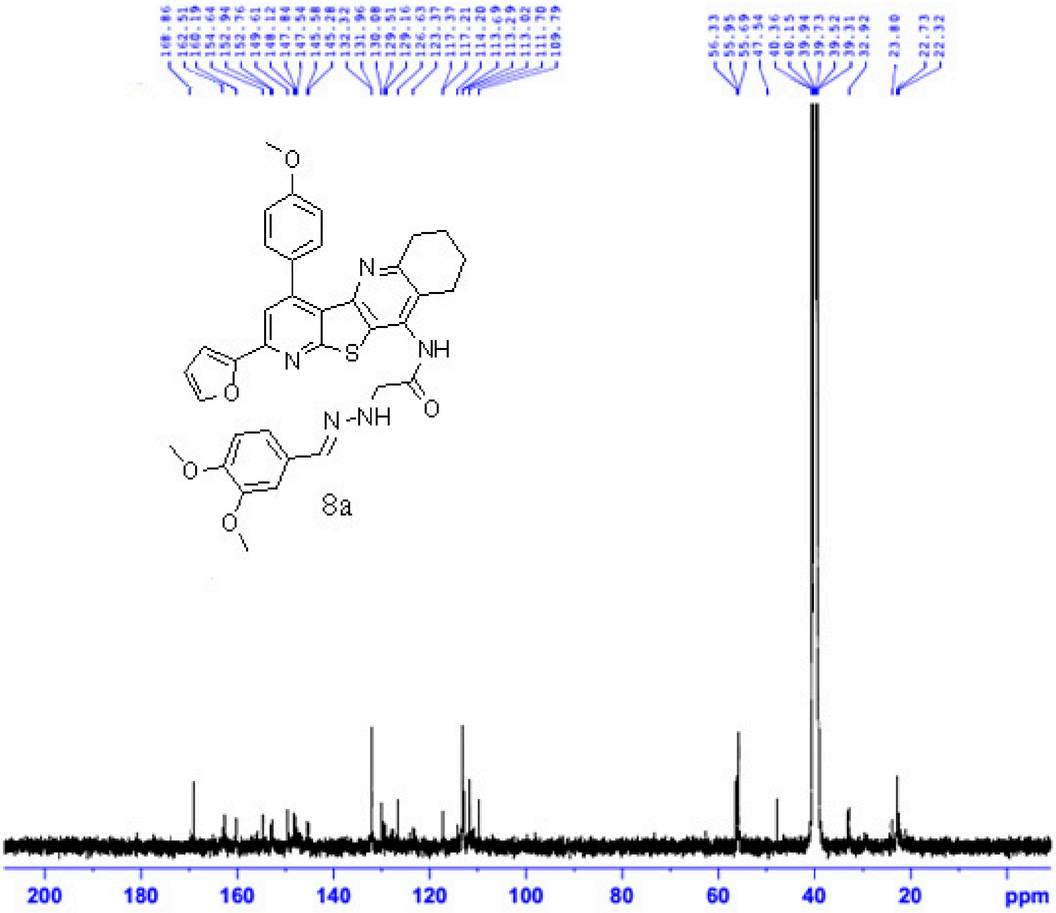

Fig. S32 ^{13}C NMR (100 MHz) in DMSO-d_6 of compound **8a**

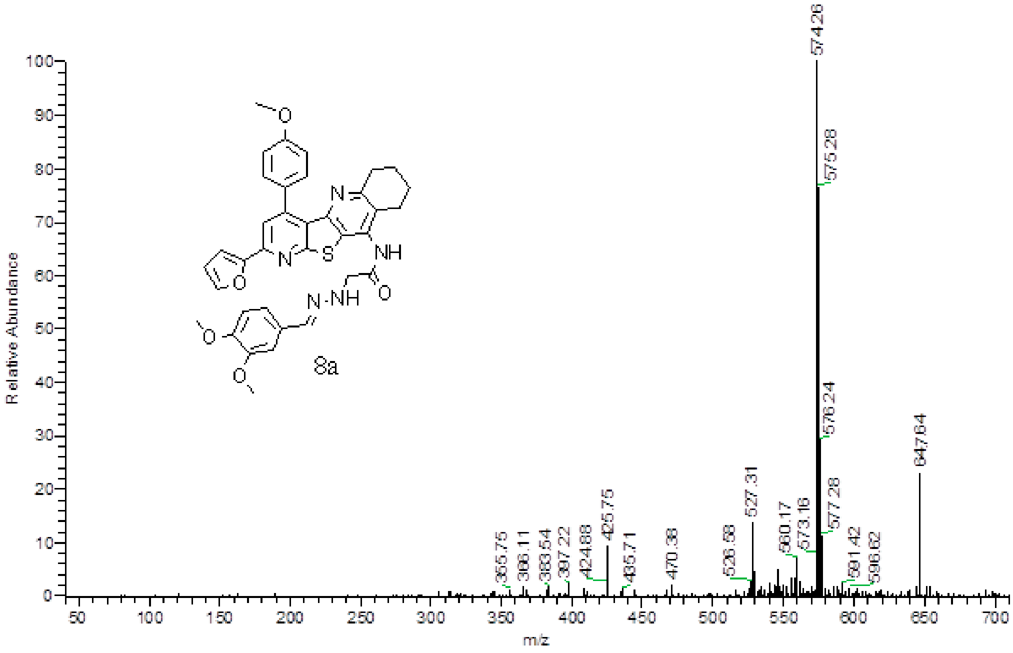

Fig. S33 Mass spectrum of compound **8a**

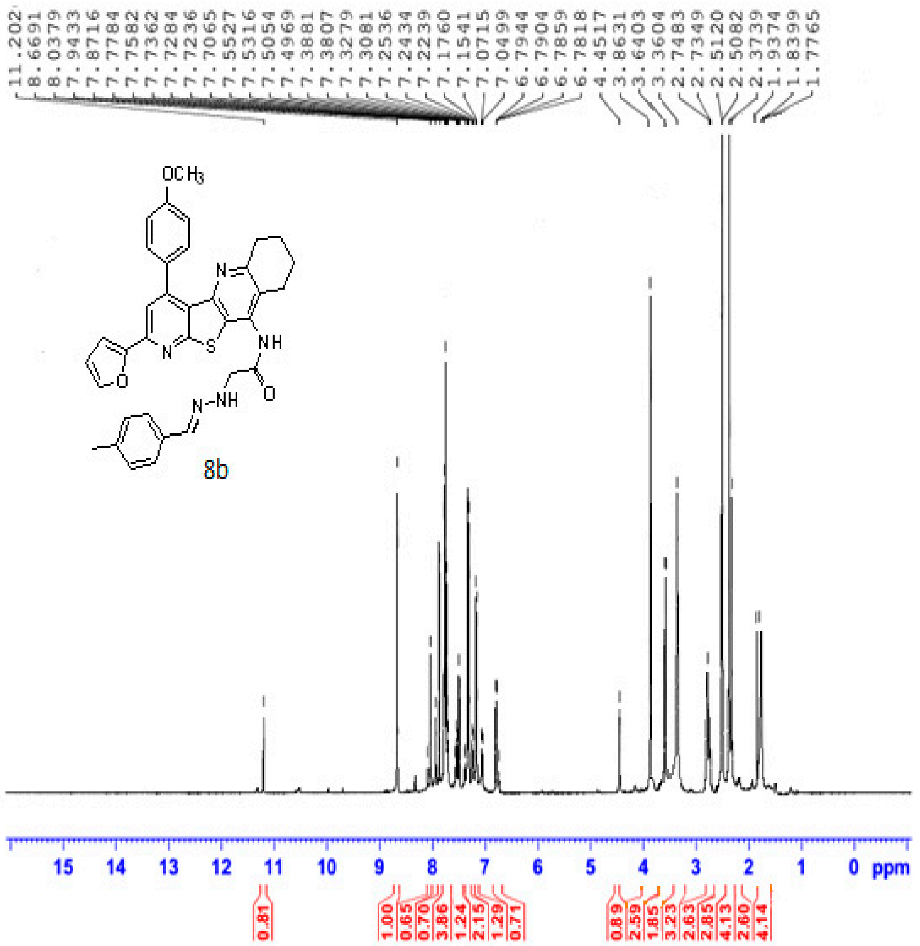

Fig. S34 ^{1}H NMR (400 MHz) in DMSO-d_6 of compound **8b**

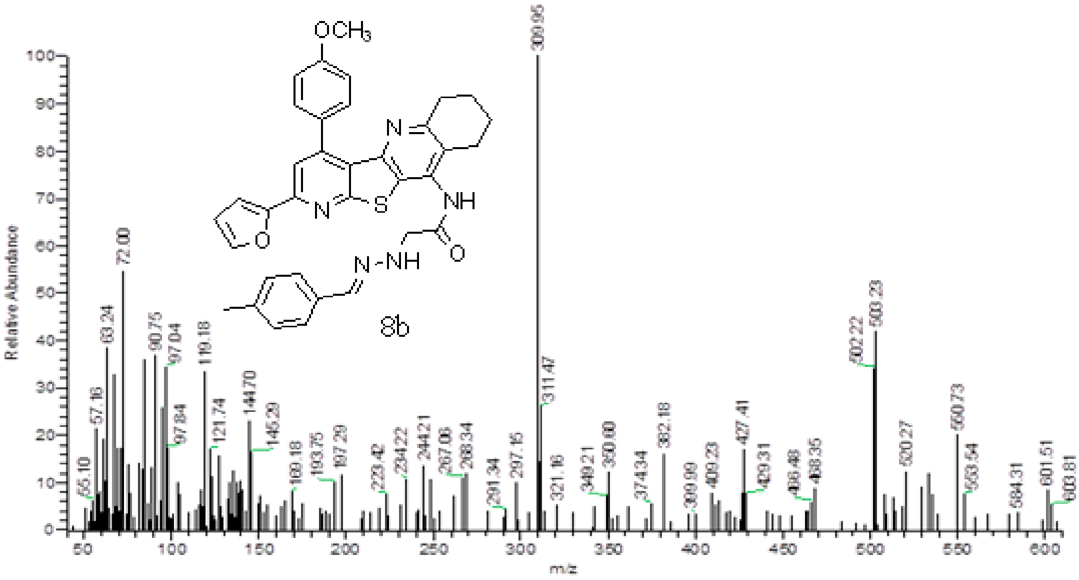

Fig. S35 Mass spectrum of compound **8b**

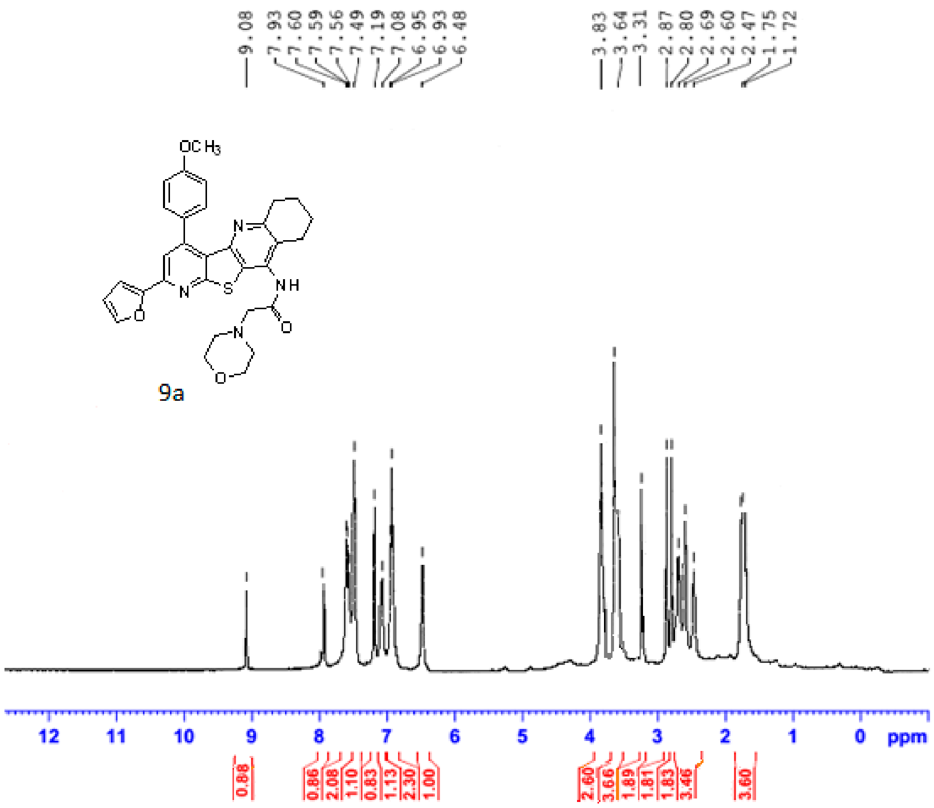

Fig. S36 ^{1}H NMR (400 MHz) in $CDCl_3$ of compound **9a**

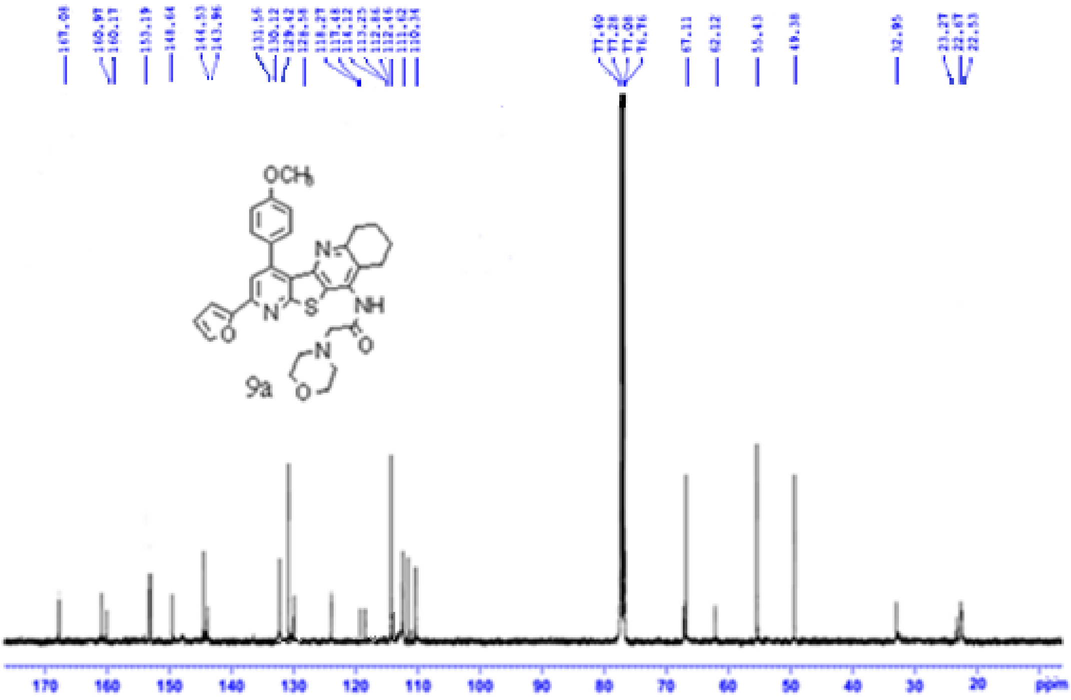

Fig. S37 ^{13}C NMR (100 MHz) in $CDCl_3$ of compound **9a**

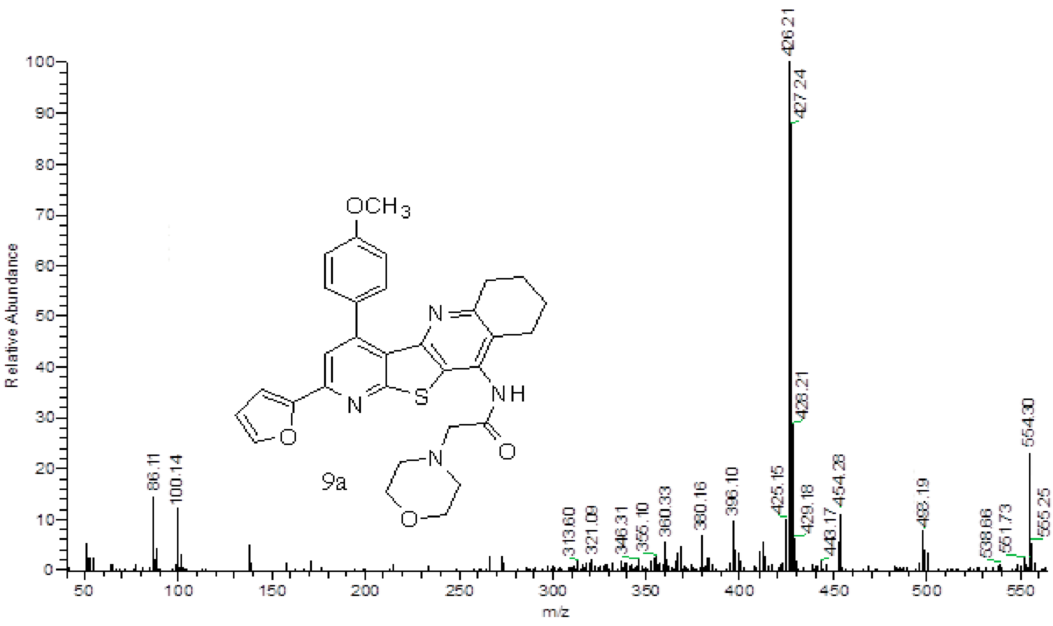

Fig. S38 Mass spectrum of compound **9a**

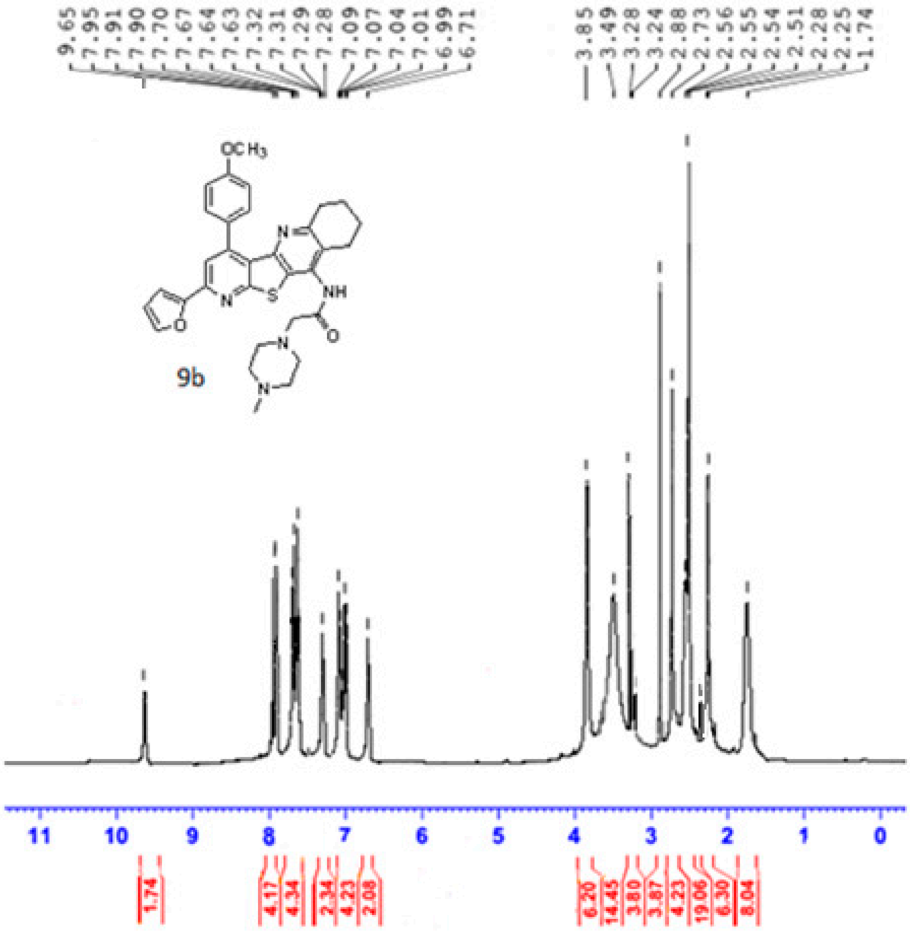

Fig. S39 ^{1}H NMR (400 MHz) in DMSO-d_6 of compound **9b**

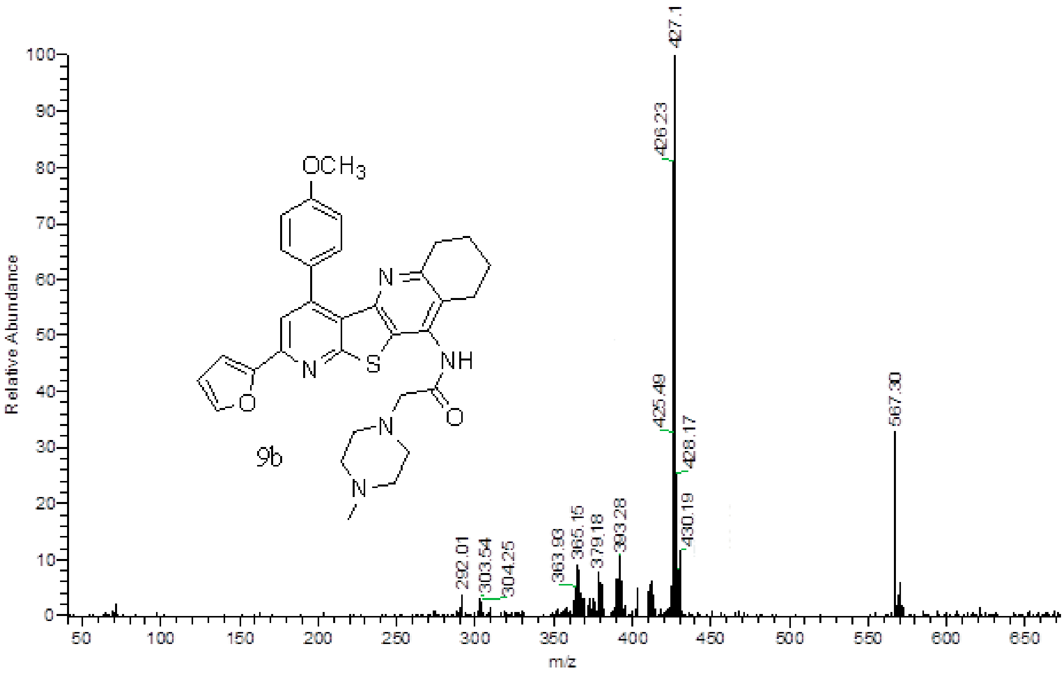

Fig. S40 Mass spectrum of compound **9b**

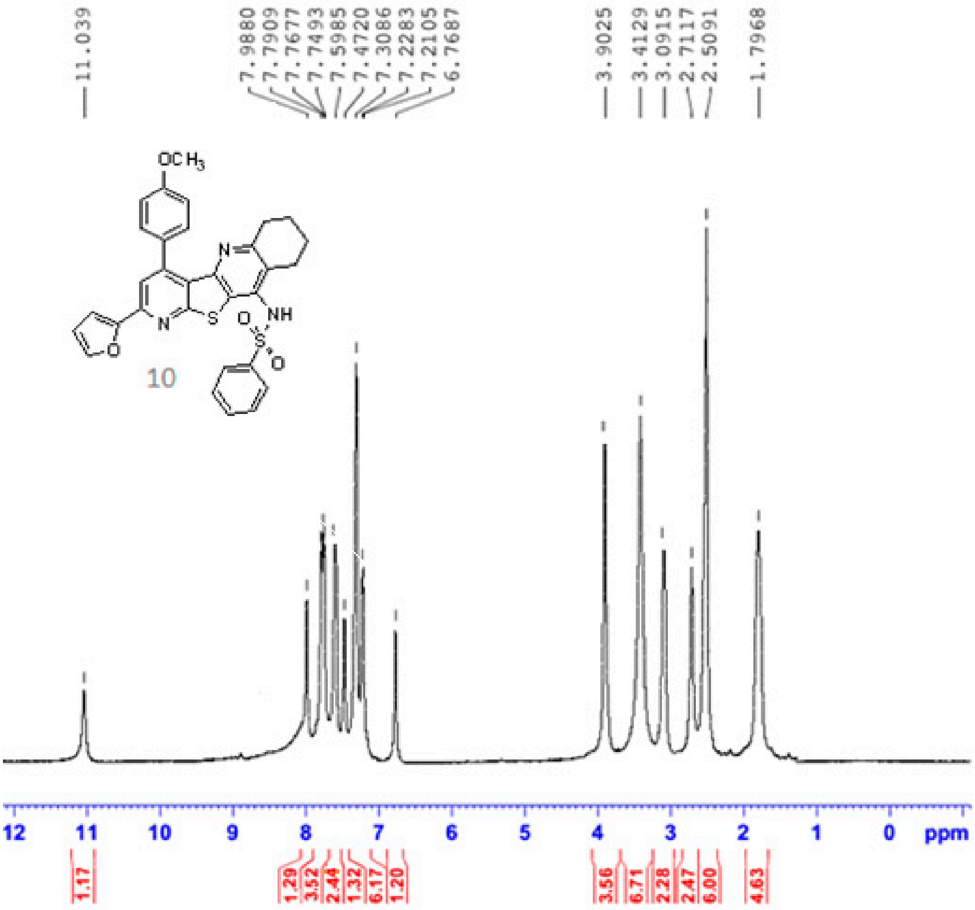

Fig. S41 ^{1}H NMR (400 MHz) in DMSO-*d*6 of compound **10**

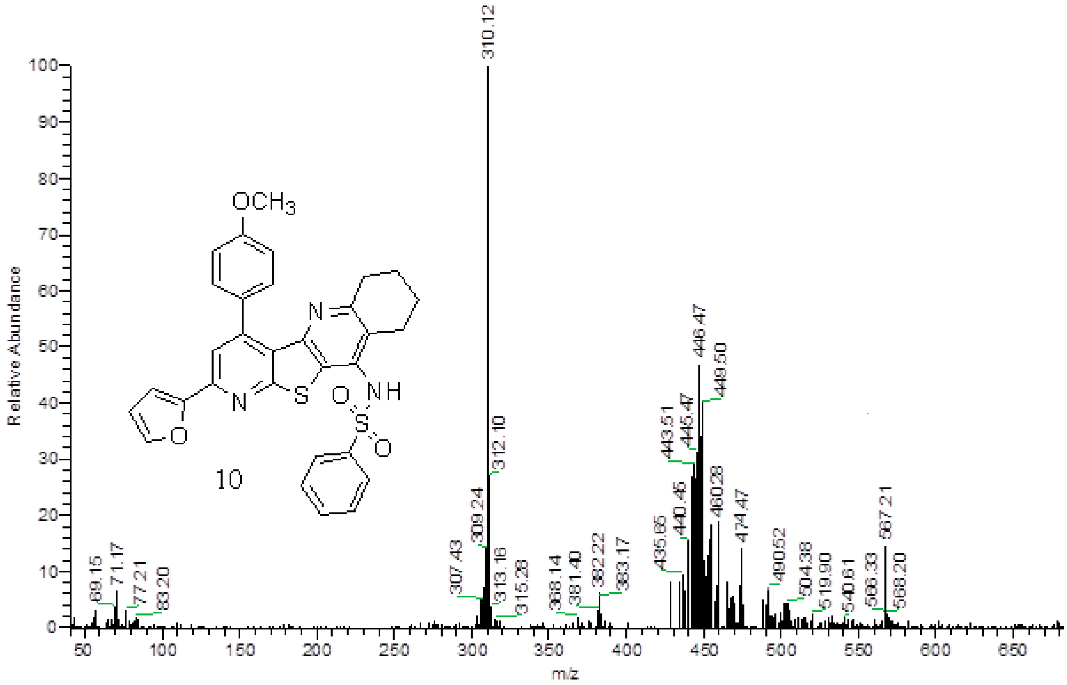

Fig. S42 Mass spectrum of compound **10**

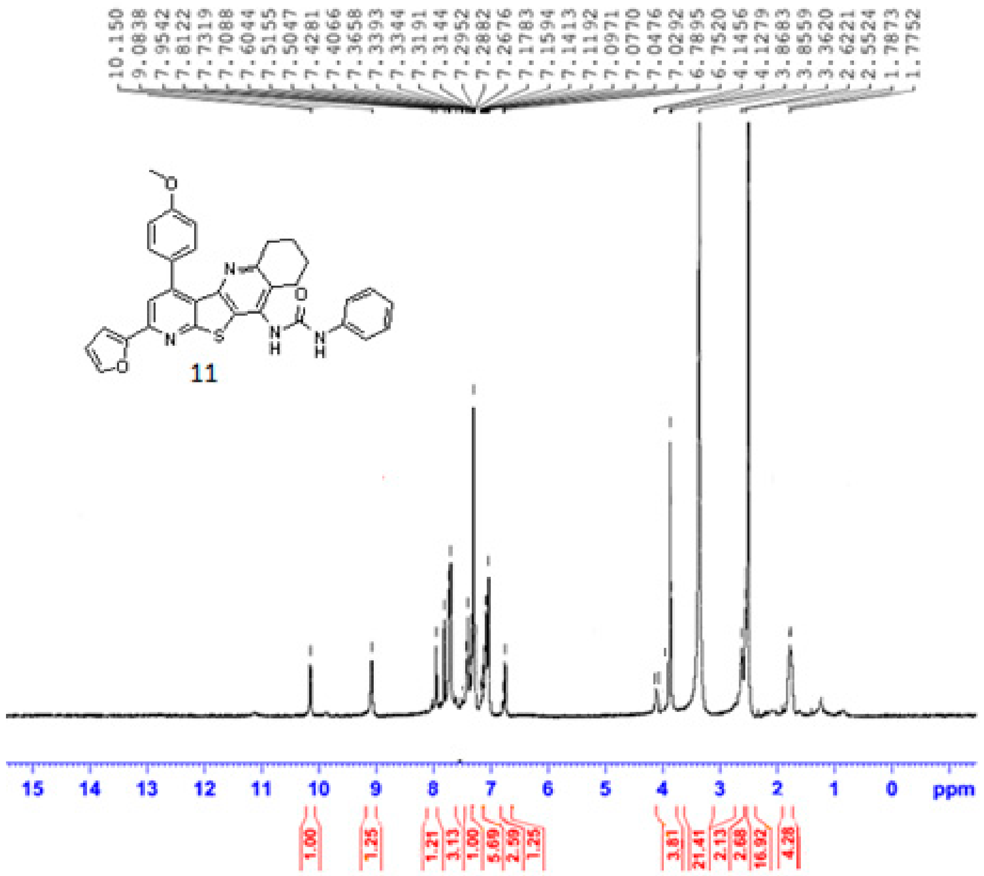

Fig. S43 ^{1}H NMR (400 MHz) in DMSO-d_6 of compound **11**

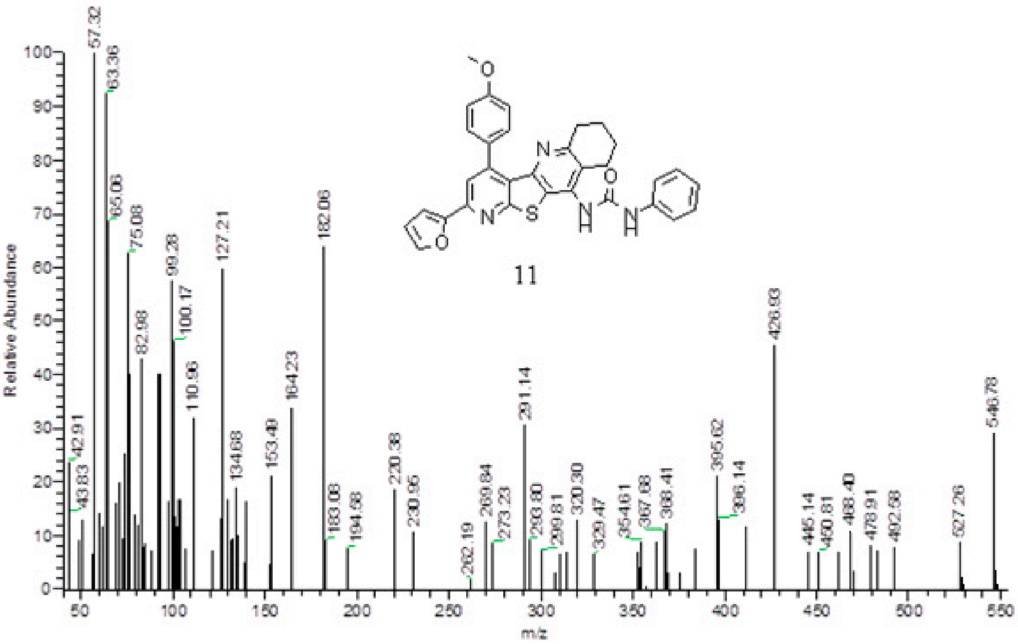

Fig. S44 Mass spectrum of compound **11**

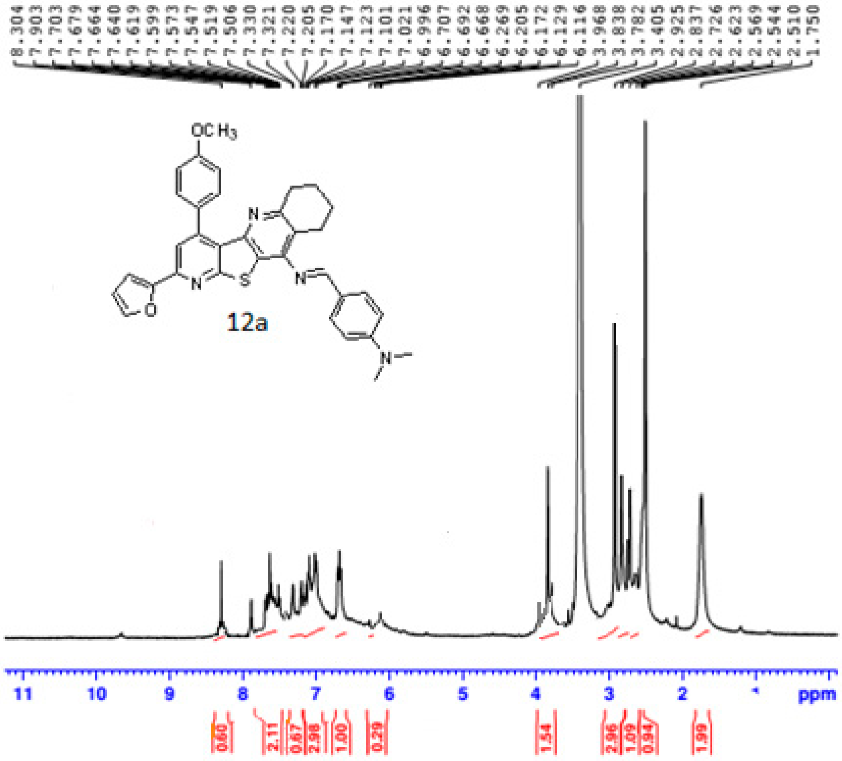

Fig. S45 ^{1}H NMR (400 MHz) in DMSO-d_6 of compound **12a**

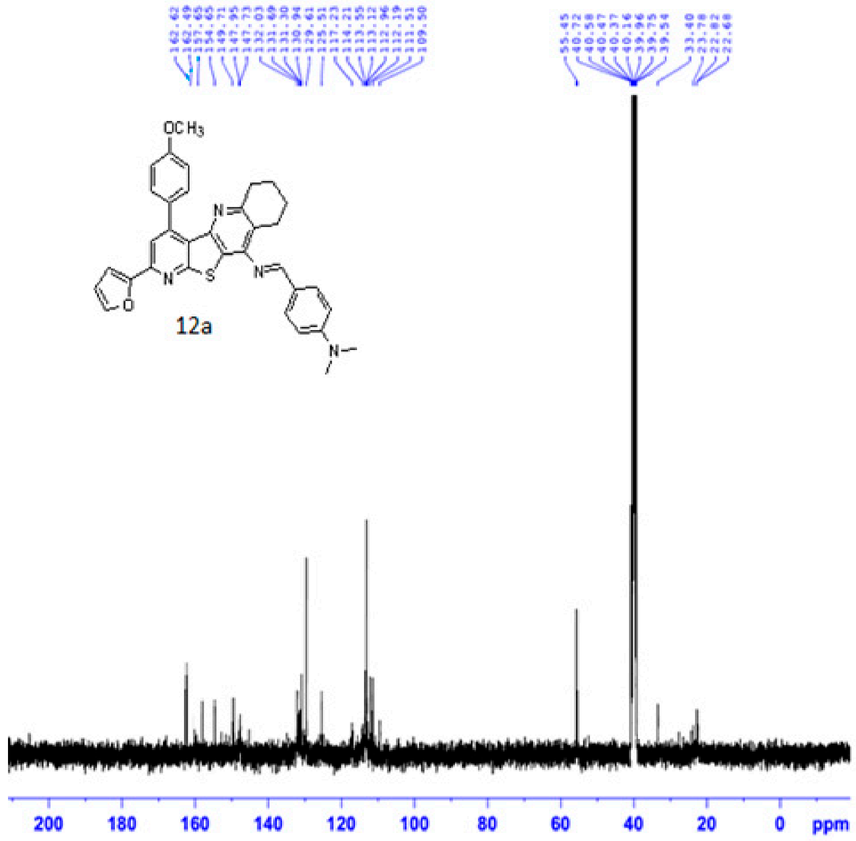

Fig. S46 ^{13}C NMR (100 MHz) in DMSO-d_6 of compound **12a**

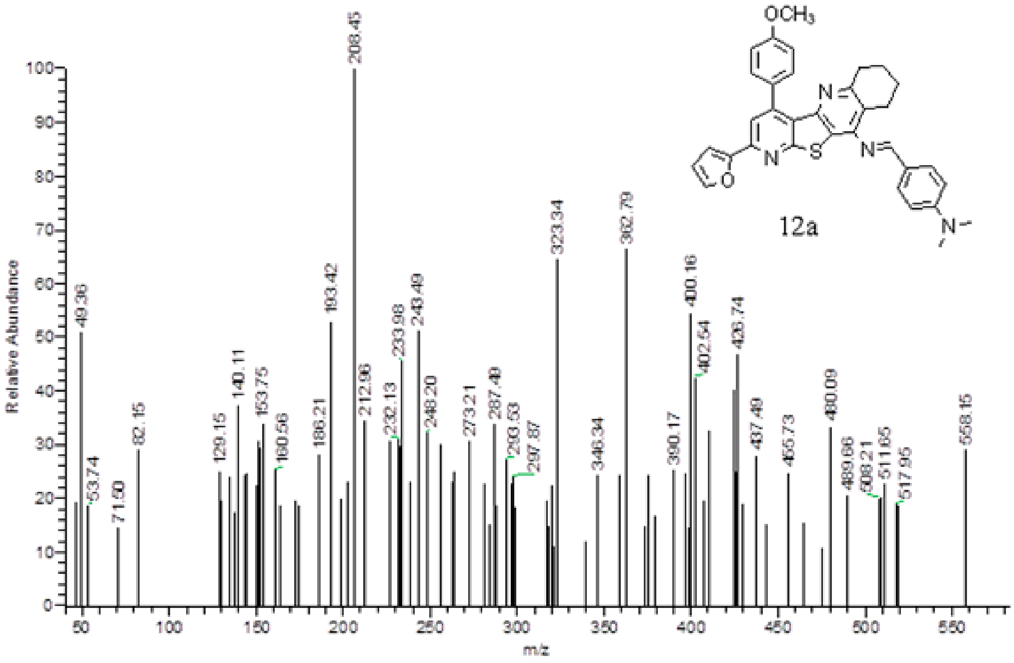

Fig. S47 Mass spectrum of compound **12a**

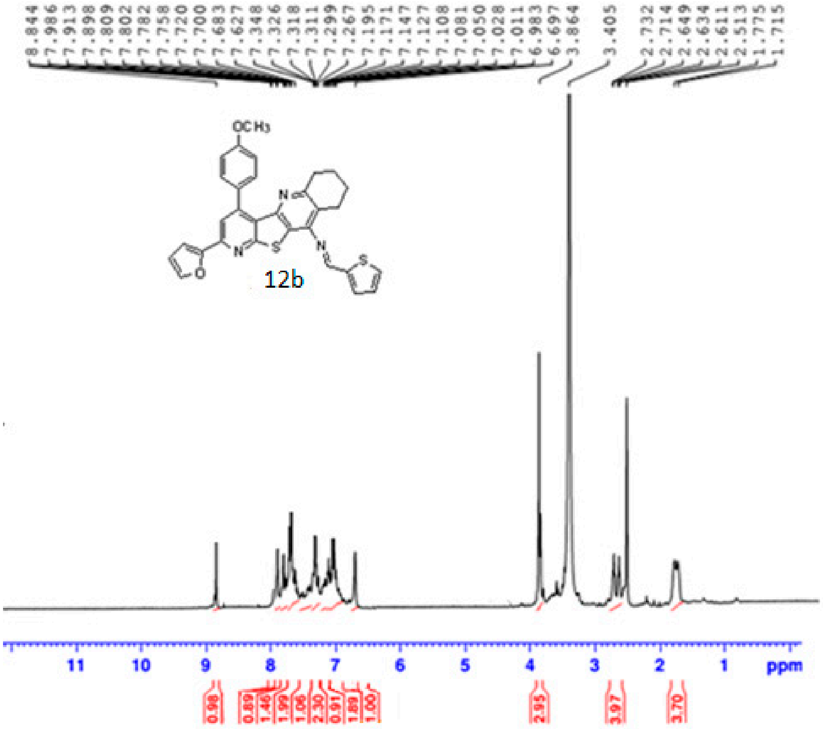

Fig. S48 ^{1}H NMR (400 MHz) in DMSO-d_6 of compound **12b**

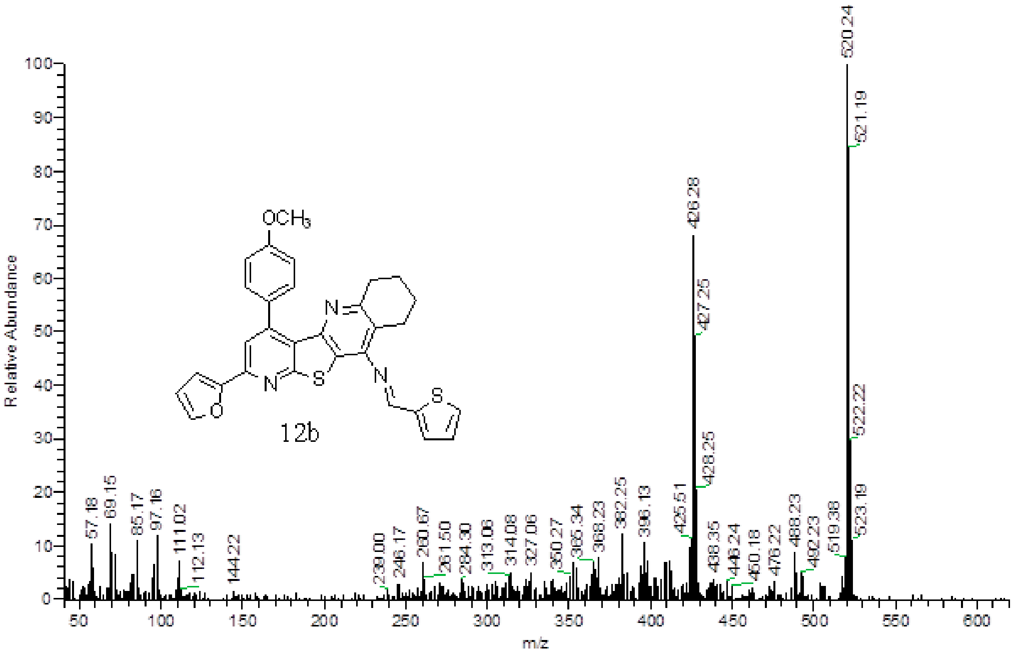

Fig. S49 Mass spectrum of compound **12b**

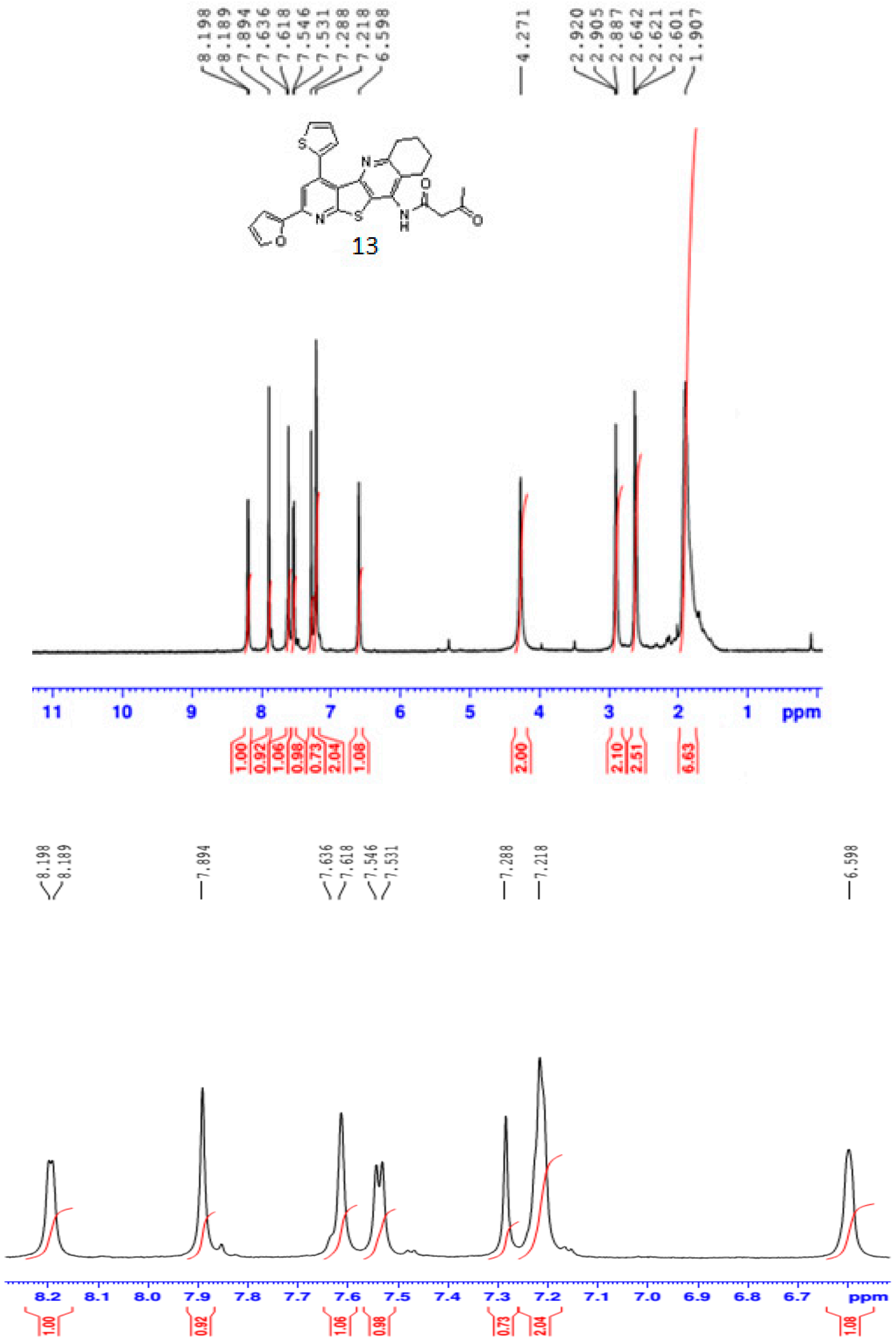

Fig. S50 ^{1}H NMR (400 MHz) in $CDCl_3$ of compound **13**

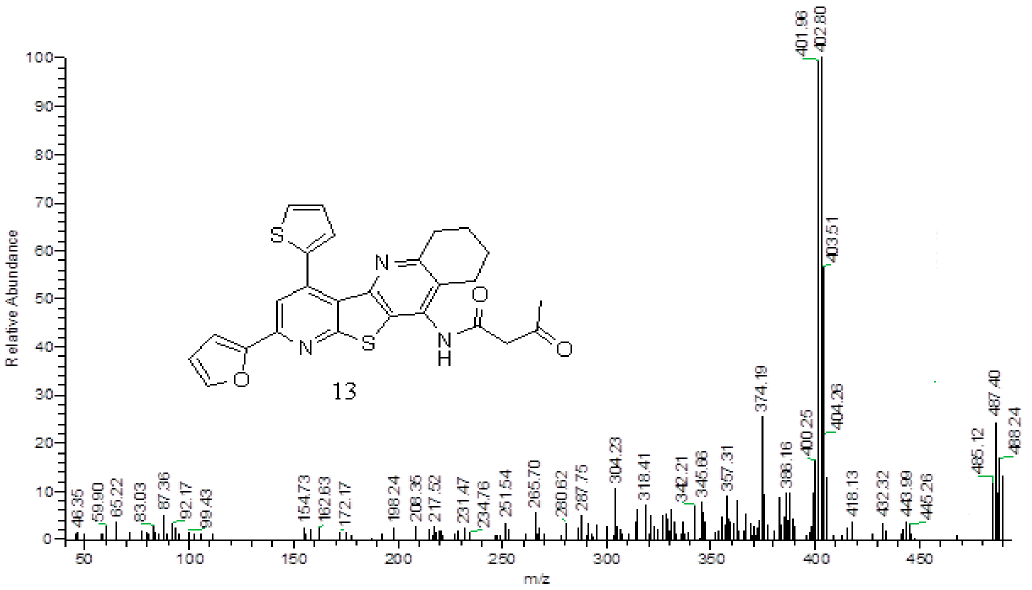

Fig. S51 Mass spectrum of compound **13**

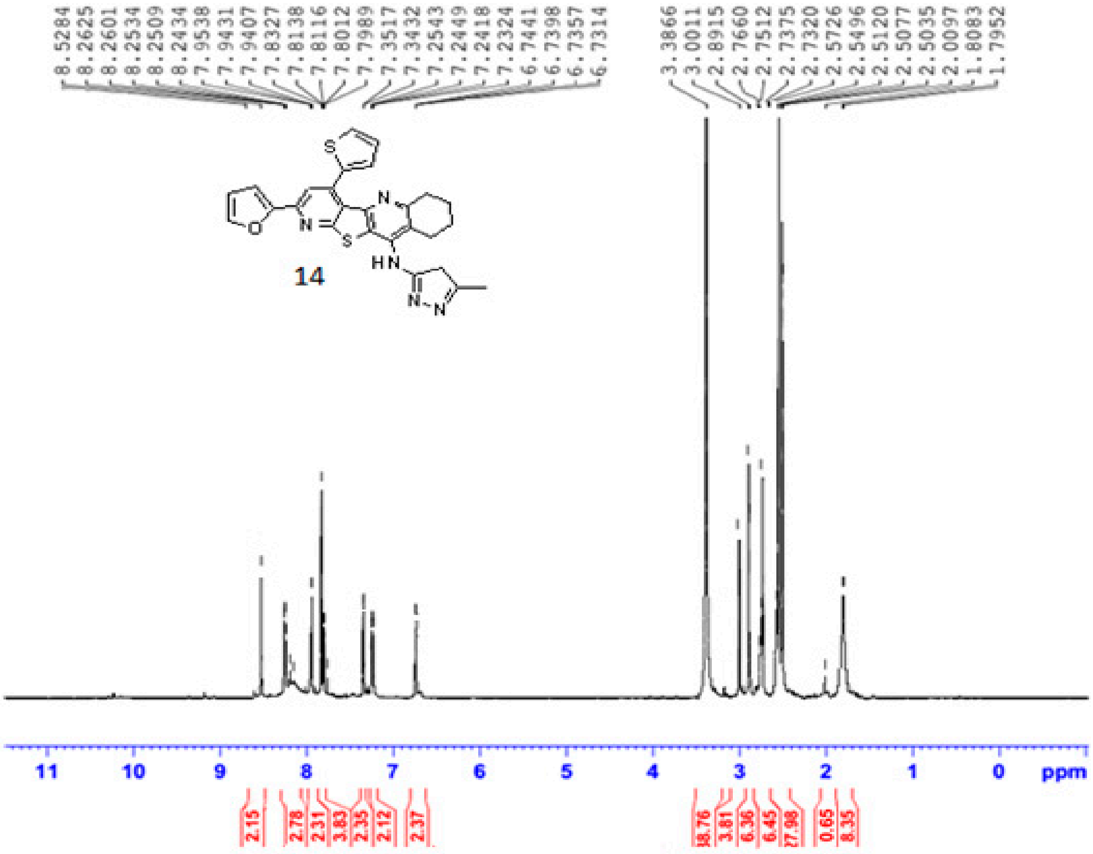

Fig. S52 ^{1}H NMR (400 MHz) in DMSO-d_6 of compound **14.**

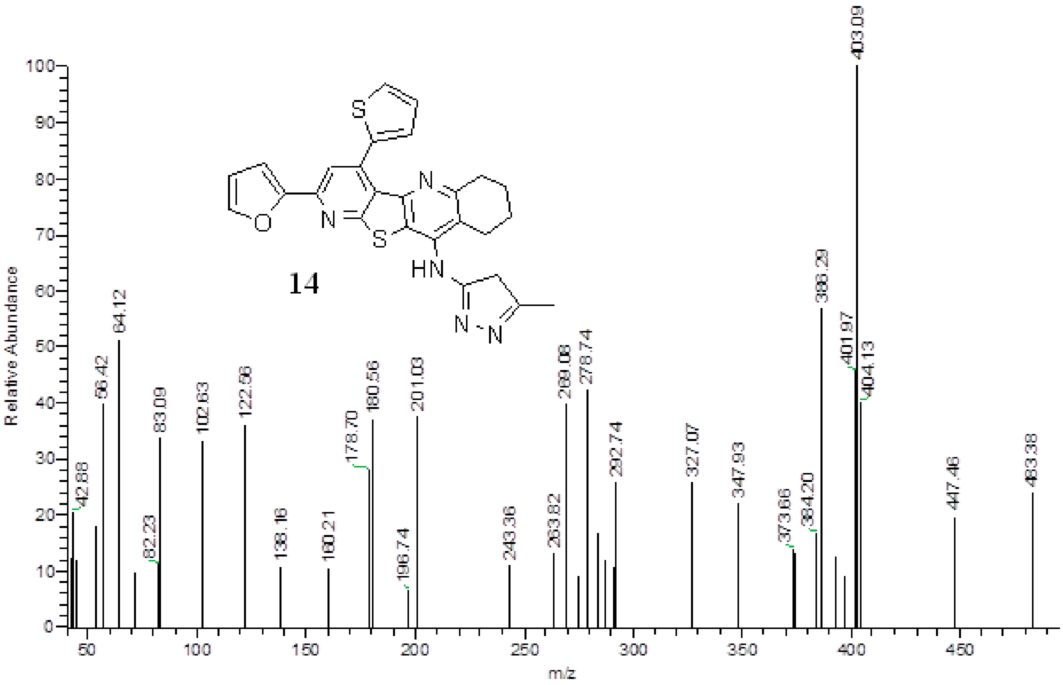

Fig. S53 Mass spectrum of compound **14**
